# Tracing metastatic spread in pediatric solid tumors using copy number and targeted deep sequencing

**DOI:** 10.1002/path.6472

**Published:** 2025-09-23

**Authors:** Natalie Andersson, Michele Ferro, Caroline Jansson, Subhayan Chattopadhyay, Jenny Karlsson, David Gisselsson

**Affiliations:** ^1^ Division of Clinical Genetics, Department of Laboratory Medicine Lund University Lund Sweden; ^2^ Division of Oncology‐Pathology, Department of Clinical Sciences Lund University Lund Sweden; ^3^ Clinical Genetics and Pathology, Laboratory Medicine Lund University Hospital Lund Sweden

**Keywords:** neuroblastoma, Wilms tumor, gonadal tumors, phylogenetics, cancer evolution, copy number alterations, mutations, SNP‐array, targeted deep sequencing

## Abstract

The most common cause of death in pediatric cancer patients is a treatment‐resistant tumor, compounded by metastatic spread, making surgery, radiation therapy, and chemotherapy unfeasible as curative treatment options. However, the mechanisms behind metastatic spread in pediatric tumors remain largely unexplored. We conducted whole‐genome copy number profiling on 171 primary tumor and metastases samples from 17 patients with neuroblastoma, Wilms tumor, or gonadal tumors, and performed targeted deep sequencing on a subset. Phylogenetic reconstruction enabled spatiotemporal tracking of subclones. In total, 11 of 17 patients displayed at least one metastasis arising earlier, defined as occurring before the most recent common ancestor in the primary tumor. In eight patients, metastatic spread was observed several times during tumor evolution, with different subclones from the same primary tumor having metastatic capability, even colonizing the same site. Strikingly, dissemination between metastases (intermetastatic spread) occurred in eight of nine patients with metastases in at least two different sites, indicating that this is a common phenomenon in pediatric malignancy. © 2025 The Author(s). *The Journal of Pathology* published by John Wiley & Sons Ltd on behalf of The Pathological Society of Great Britain and Ireland.

## Introduction

In pediatric cancer patients, the leading cause of death is treatment‐resistant relapse, often compounded by metastatic spread to several anatomical sites. Consequently, conventional therapeutic modalities, such as surgery and radiation therapy, often become unfeasible as curative treatment options. Therefore, there is a need for novel treatment strategies that consider the intricate subclonal dynamics of metastatic progression. Recent advances have focused on the timing of metastatic dissemination and how different metastases are related to each other and to the primary tumor in neuroblastoma [1]. There is, however, a lack of studies that have tracked subclones to systematically map metastatic patterns in Wilms tumor (WT) and gonadal tumors (GT).

In this study we sought to trace the evolutionary trajectories of metastatic disease in three pediatric malignancies: neuroblastoma (NB), WT, and GT. NB originates from the developing sympathetic nervous system, most often arising in the adrenal medulla [2]. The median age at diagnosis is 18 months, and the tumors often display gains of 17q, deletions of 1p and 11q, and *MYCN* amplifications, of which the latter two aberrations correlate with worse prognosis [3, 4, 5]. The most common locations for metastatic colonization are locoregional lymph nodes, the bone marrow, and the skeleton [6]. WT is a kidney tumor, most often occurring in children below the age of 5 years. In 5%–8% of patients it appears bilaterally or multifocally [7]. It commonly harbors loss of heterozygosity of 11p, including the *WT1* gene, as an early change. In addition, *TP53* mutations, gain of 1q, and *MYCN* alterations have been associated with more aggressive disease [8, 9, 10, 11, 12, 13]. GT comprise a heterogenous group of different tumor subtypes, which usually arise in the testes and ovaries but can appear at other anatomic locations, such as the neck, oral cavity, or brain [14].

Tumors are commonly believed to originate from a single cell that undergoes malignant transformation via genetic and phenotypic alterations. The accumulated genetic alterations are conveyed to each tumor cell's daughter cells. In this way, subpopulations (subclones) of cancer cells are formed in the primary tumor. Some alterations provide a fitness advantage, some are neutral, while others cause a disadvantage, resulting in some subclones that thrive, and others that diminish, mimicking ecological selection. Moreover, antitumoral therapies might exert selective pressures, resulting in varying drug responses across patients and a treatment‐induced selection of resistant cell populations. The latter is particularly significant in the context of molecularly targeted drugs, where the presence or absence of the target within different tumor regions can affect treatment efficacy [15]. This selection process is akin to Darwinian selection of cancer cell populations, as proposed by Peter Nowell already in 1976, and results in a genetic and phenotypic heterogeneity across space and time [16]. The presence of intratumoral and intertumoral heterogeneity is now widely acknowledged in almost all adult cancers [17, 18], and has also been demonstrated in pediatric tumors [19, 20]. This heterogeneity underscores the necessity of sampling several anatomical regions to comprehensively characterize the subclonal landscape of the tumors.

**Figure 1:**
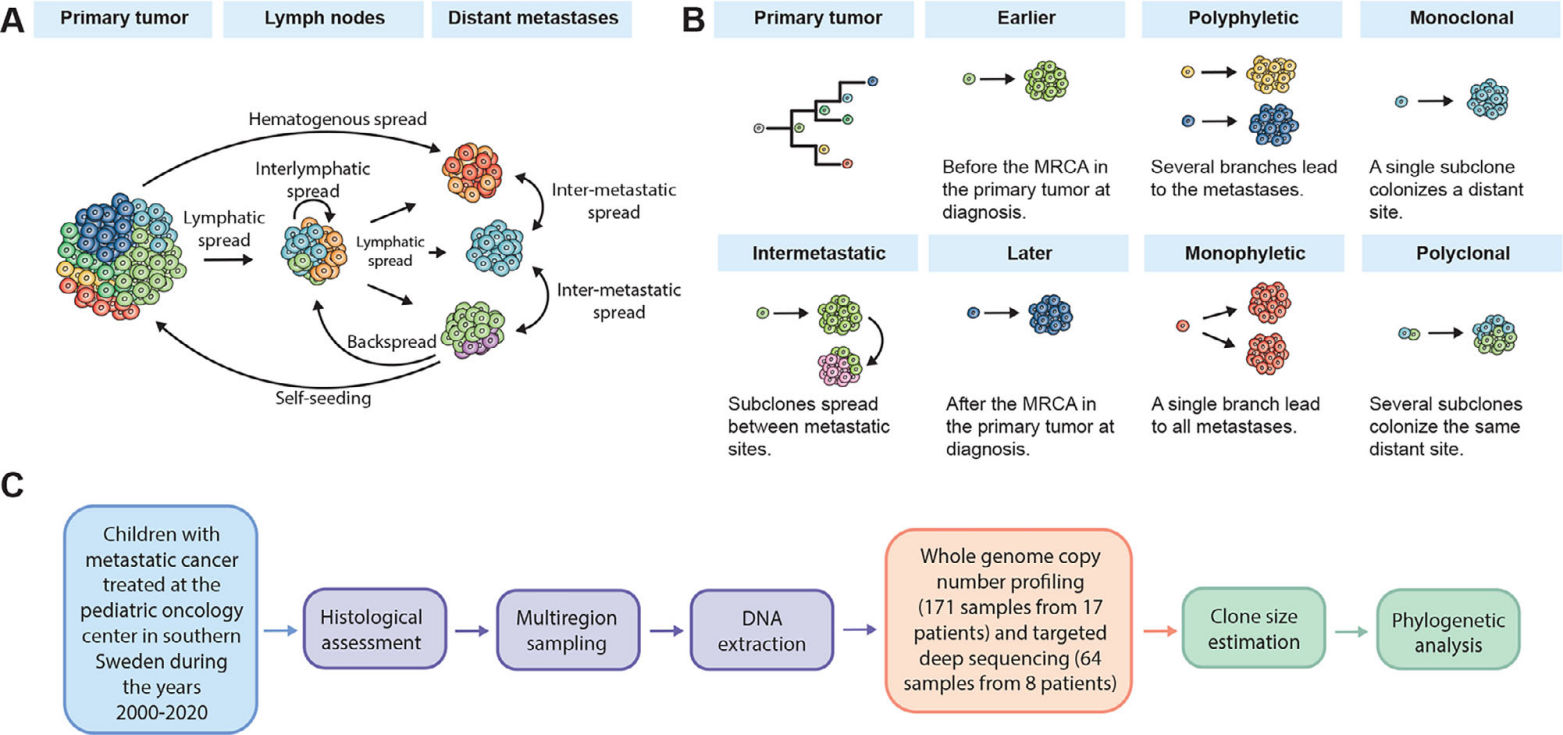
Overview of the research question and workflow. (A) Overview of different possible modes of metastatic spread. Usually, metastatic spread is depicted as occurring either via lymphatic or hematogenous spread. There may, however, be interlymphatic spread, intermetastatic spread (cancer cells spreading between metastatic sites), or even instances of back spread to the lymphatic system or self‐seeding, where cancer cells recolonize the primary tumor site. (B) In this study we defined earlier metastatic spread as occurring before, and later as occurring after the most recent common ancestor (MRCA) in the primary tumor. Polyphyletic spread means that several different phylogenetic branches are responsible for the metastases, while monophyletic refers to all metastases have arisen from the same phylogenetic branch. Monoclonal spread means that a single subclone colonizes a distant site. In polyclonal seeding, several different subclones colonize the same distant site. Intermetastatic spread is the spread of tumor cells between metastatic sites. (C) Simplified workflow of this project. Histological assessment was performed to select tumor areas that were sampled. DNA was extracted from each area. Whole‐genome copy number profiling was performed on all samples, and targeted deep sequencing on a subset from which there was enough DNA. Based on these data, clone sizes could be estimated and phylogenetic trees reconstructed.

Metastatic spread typically occurs via the lymphatic system or hematogenously (Figure 1A). Tumor cells spreading between different lymph nodes or distant metastatic sites within the same patient have, however, been observed, a phenomenon denoted intermetastatic spread [21, 22, 23]. Its prevalence across pediatric tumors does, however, still remain unclear. One study has found evidence of intermetastatic spread in NB, but it has never been investigated in the context of WT nor GT [1]. A profound implication of intermetastatic spread could be that a solitary metastasis spawns new metastatic lesions, even after removal of the primary tumor.

Furthermore, circulating tumor cells hold the potential to re‐enter the lymphatic system or recolonize the primary tumor site itself, referred to as self‐seeding [24]. In addition, the temporal dynamics of metastatic dissemination profoundly influence treatment decisions and patient outcomes. While the timing of metastasis onset (whether earlier or later during the course of the disease) and pathways of metastatic dissemination, such as monoclonal *versus* polyclonal seeding, and monophyletic *versus* polyphyletic dissemination, have been extensively explored in adult cancers [21, 22, 23, 25, 26, 27, 28, 29, 30], these processes remain largely understudied in pediatric tumors. Most knowledge has been confined to one study on NB, with no studies, to our knowledge, conducted on WT and GT. Despite the impressive number of patients analyzed in the NB study, only 10 out of the 283 patients had metastases at several sites [1, 6]. Nevertheless, this study provided evidence for both intermetastatic spread and polyclonal seeding in NB. Insights into the prevalence and dynamics of these evolutionary routes across pediatric solid malignancies could wield significant influence on metastatic disease management of metastatic disease in pediatric oncology.

The present study consequently aims to address these gaps in our understanding of metastatic dissemination in NB, WT, and GT by conducting whole‐genome copy number profiling of 171 samples from 17 pediatric cancer patients, including samples from primary tumors, metastases, and samples obtained before and after treatment. Moreover, targeted deep sequencing was performed on 64 samples from 8 patients. Leveraging phylogenetic analysis, we unravel the spatiotemporal evolution of cancer cell subclones, shedding light on metastatic trajectories, across several pediatric solid tumor types (Figure 1B,C).

## Materials and methods

### Ethics approval

The study was conducted in accordance with the Declaration of Helsinki and was approved by the regional Ethics Committee (L289‐11) updated for further data‐ and material‐collection in 2017, and then renewed and approved in 2023 by the Swedish Ethics Review Authority (2023‐01550‐01).

### Experimental design

The patient cohort consists of children with solid tumors who progressed and/or relapsed during the years 2000–2020, treated at the regional pediatric oncology center in southern Sweden during primary, progression, and relapsed disease. In the supplementary material, File S1A, information regarding the age, genetic sex, and tumor site is enclosed. Tumor material from the primary tumor, relapses, and several different metastatic sites have continuously been stored at room temperature as formalin‐fixed paraffin‐embedded (FFPE) samples at the pathology department at the Skåne University Healthcare System, Lund, Sweden. An overview of the analysis workflow is provided in Figure 1C and supplementary material, Figures S1 and S2.

Hundreds of histological slides were assessed microscopically and areas with a sufficient amount of tumor cells (>30% tumor cell content), were chosen for further analyses. Considering the spatiotemporal genetic heterogeneity of tumors, several tumor areas from different regions of the primary tumor and metastases were selected for each patient. In total, 253 tumor regions were initially selected from 22 patients. Tissue was excised with standard techniques used in the clinic and DNA was extracted from each area using the Qiagen AllPrep DNA/RNA FFPE kit (Qiagen, Hilden, Germany) according to the manufacturer's instructions. Aggregated DNA was avoided using a thermoshaker at 37 °C, 1,200 rpm for a minimum of 4 h and vortexing each tube every hour for a minimum of 20 s. DNA quantification was performed using the Qubit fluorometer. A total of 184 samples from 20 patients had a sufficient DNA quantity to be included for whole‐genome copy number profiling.

### Copy number profiling

The extracted DNA was subjected to whole‐genome copy‐number profiling using the SNP‐array Oncoscan CNV assay (Affymetrix, Santa Clara, CA, USA) according to standard protocols conducted at Eurofins, Galten, Denmark. No matched normal sample was used, since the normal cell karyotype was assumed to be diploid. The obtained channel‐specific CEL‐files, corresponding to the AT and GC channels, were used with the software Chromosome Analysis Suite (ChAS) to generate both OSCHP‐files, using the OncoScan_CNV.na36.r2.annot.db (reference genome build hg38), and to generate probe‐level and segment‐level data. Of the 184 samples from 20 patients who underwent whole‐genome copy number analysis, three patients were excluded due to noisy data or no data from metastases, resulting in 171 samples from 17 patients encompassing a median of eight (range 3–22) samples available per patient, with a median of three (range 1–10) samples from the primary tumor and three (range 1–19) samples from metastases per patient (supplementary material, File S1A and Figure S39).

The OSCHP‐files were visualized using ChAS. Only clearly visible genetic alterations, ≥30 kBp in size and with a marker count ≥30 were included in further analyses. Constitutional copy number variants were omitted based on manual comparison against the Database of Genomic Variants with the reference genome hg38. All detected CNAs were validated through manual inspection, and when mutations were absent in some samples, their absence was carefully verified through a careful review process.

Probe‐ and segment‐level data from ChAS was used to generate Tumor Aberration Prediction Suite plots using TAPS (v.2.0, RRID:SCR_000356), showing the relative probe intensity ratios in log2‐scale (log2R) on the x‐axis and the allelic imbalance on the y‐axis for each chromosomal segment [31]. This generated a pattern that allowed the determination of the allelic composition for each detected genetic alteration. It also made it possible to identify copy number neutral imbalances (CNNIs), which cannot be discerned via $\log_{2}R$ alone but are only detectable in the TAPS‐ and B‐allele frequency (BAF)‐plots in ChAS.

The mutated sample fraction (MSF), representing the proportion of cells in the sample harboring each genetic alteration, was calculated for each chromosomal segment using the following equation, as previously described [32]:

(1) $$\mathrm{MSF}=\frac{N_{p}\cdot 2^{\log_{2}R_{\text{ChAS}}}-N_{p}\;}{N_{t}-N_{p}}$$

where $\log_{2}R_{\text{ChAS}}$ is the log2‐ratio as indicated by ChAS for the chromosomal segment, $N_{p}$ is the number of alleles of the background cells, i.e. the ploidy level to which the $\log_{2}R_{\text{ChAS}}$ has been normalized against, and $N_{t}$ is the number of alleles of the genetic alteration in question. For CNNIs, the allelic composition of each alteration together with their BAFs were used for MSF computation according to the following equation:

(2) $$\begin{array}{c} \mathrm{MSF}=\frac{1-2\cdot \text{mBAF}}{\text{mBAF}\left(N_{A}+N_{B}-2\right)-N_{B}+1\;} \end{array}$$

where mBAF is the mirrored B‐allele frequency, $N_{A}$ is the number of A‐alleles, and $N_{B}$ is the number of B‐alleles and $N_{B}>N_{A}$.

The mutated clone fraction (MCF) i.e. the proportion of nonnormal cells harboring a specific genetic alteration was calculated by dividing the MSF by the tumor cell fraction (TCF):

(3) $$\begin{array}{c} \mathrm{MCF}=\frac{\mathrm{MSF}}{\mathrm{TCF}} \end{array}$$

The TCF was obtained for each sample by calculating the mean MSF‐value of the clearly clonal genetic alterations in TAPS using log2R. All genetic alterations, in that specific sample, were normalized against this value. The interval of clonal events was computed as:

(4) $$\begin{array}{c} \text{Interval of clonal events}=\frac{\mathrm{TCF}\pm 2\cdot {\mathrm{SD}}_{\mathrm{MSF}}}{\mathrm{TCF}} \end{array}$$

where $SD_{\mathrm{MSF}}$ is the standard deviation of the MSF‐values used to compute the TCF in that sample. Genetic alterations with an MCF within this interval were categorized as clonal and their MCF was set to 100%. The remaining genetic alterations were hence categorized to be subclonal, and their MCFs remained unchanged. Supplementary material, Figure S1 illustrates the workflow for CNA data analysis. All segment files used in this study, including the genetic alterations identified in each sample, for each patient along with their MCFs can be seen in the supplementary material, File S1.

### Targeted deep sequencing

A total of 151 samples (including matched normal samples) from 16 patients, with sufficient DNA concentration, as measured using the Qubit Flex Fluorometer, were selected for targeted deep sequencing (TDS). Of these, tumors from eight patients had DNA of sufficient quality to be included for library preparation and sequencing, encompassing 63 tumor samples and 8 matched normal samples for further analysis.

DNA library preparation was performed using the protocol KAPA HyperPlus kit. Since the samples also contained EDTA‐buffer, which might interfere with the sequencing, a conditioning solution was added, as part of the standard protocol, to each sample. TDS was performed using the NovaSeq 6,000 sequencing system using paired‐end sequencing up to 10,000 × sequencing depth. The GMCK solid tumor pancancer gene panel (Lindberg panel), encompassing 300 genes, was used (supplementary material, File S5). Demultiplexed fastq‐files were provided. Quality control, library preparation, sequencing, and demultiplexing were performed by the Center for Translational Genomics in Lund (CTG).

The Burrows‐Wheeler Aligner method, implemented in bwamem2 (v.2.2.1, https://github.com/bwa-mem2/bwa-mem2) was used to align the tumor samples' genomes to the reference genome hg38 [33]. Since each sample was paired‐end sequenced, two fastq‐files were generated for each sample, and both were used as input to bwamem2. This resulted in the generation of one BAM‐file for each sample, which was sorted and indexed using SAMtools (v.1.15.1, RRID:SCR_002105) [34]. Polymerase chain reaction (PCR)‐duplicates were marked using MarkDuplicates from Picard tools (v.2.22.1, RRID:SCR_006525). Base quality score recalibration was performed with GATK (v.4.1.3.0) [35, 36]. This was followed by variant calling. Both Mutect2 [37] (RRID:SCR_000559, used according to standard practice, including the flags: ‐‐L, ‐‐interval padding 70, ‐‐panel‐of‐normals, ‐‐germline‐resource, ‐‐flr2‐tar‐gz), Manta [38] (v.1.2.2, RRID:SCR_022997, with the flags: ‐normalBam, ‐‐tumorBam, ‐‐callRegions, ‐‐exome, ‐‐referenceFasta), Strelka2 [39] (v.2.8.4, RRID:SCR_005109, with the flags: normalBam, ‐‐tumorBam, referenceFasta, ‐‐exome, ‐‐indelCandidates (obtained from Manta)) and VarScan2 [40] (v.2.4.1, RRID: SCR_006849 in the somatic mode) were used for variant calling, generating vcf‐files. All variant calling was performed together with a paired normal sample from the same patient. Germline alterations were not included in the analysis. They will be present in all cells in the tumor and will merely be confined to the stem, thus not providing information for phylogenetic inference and tracking of subclones.

Variants were filtered using the recommended filtering software for each variant caller and only variants passing all internal filters were included for further analysis. Subsequently, variants were removed if they were found in smallexac, gnomAD, and a panel of normals as well as all variants having a mapping quality <50. This was followed by annotation using Annovar (v.2017Jul16‐Perl‐5.26.0, RRID:SCR_012821) [41].

To reduce the number of false‐positive variants, additional filtering was implemented using a customized R script. Variants were excluded if:

1. VAF < 0.05 in the tumor sample
2. VAF > 0.05 in the normal sample (normal samples were collected from the periphery of the tumor, and there may be some minor contamination of migrating cells)
3. Read depth (DP) < 20 in either the normal sample or the tumor sample

Of the remaining variants, all were kept that fulfilled any of these criteria:

1. Detected in at least two different samples and by at least two different variant callers in total.
2. If the variant was only detected in one sample, two different variant callers must have called it for it to be kept.

In addition, only variants covered by the BED‐file obtained from the manufacturer were kept for analysis.

Mutated clone fractions (MCFs), representing the proportion of cancer cells having each variant, were calculated from the VAFs using the equation:

(5) $$\mathrm{MCF}=\frac{\mathrm{VAF}\cdot \left({\mathrm{CN}}_{1}\cdot \mathrm{TCF}\cdot f_{1}+CN_{2}\cdot \left(\mathrm{TCF}-\mathrm{TCF}\cdot f_{2}\right)+2\cdot \left(1-\mathrm{TCF}\right)\right)}{M\cdot \mathrm{TCF}}$$

where VAF is the variant allele frequency, $CN_{1}$ is the copy number at the location of the identified mutation, and f1 is the MCF of the tumor cell population having the CNA, $CN_{2}$ is the copy number, and f2 is the MCF of the tumor population not having the variant, TCF is the tumor cell fraction, the 2 represents the number of alleles in the normal cell population, and M is the number of alleles in the sample having the variant. M was chosen to be 1 as a default. If the MCF became >1.1 it was increased to 2.

### Phylogenetic tree reconstruction

There are several published software tools that aim to perform clustering of mutations and/or subclonal deconvolution. Popular algorithms such as PyClone, SciClone, PhyloWGS, and LiCHeE, require sequencing data such as WES or WGS to operate [42, 43, 44, 45], making them unfeasible to apply using formalin‐fixed paraffin‐embedded samples. MEDICC2 is a software specifically designed for CNA evolution, but it does not allow integration of both CNAs and point mutations, and it assumes that there is only one subclone in each sample, i.e. that each copy number is clonal [46]. Hence, most softwares are either limited to sequencing data, or do not allow the integration of copy number alterations and point mutations in the subclonal deconvolution procedure for multiregional sampling data from the same patient [47]. We therefore chose to use the DEVOLUTION algorithm, which allows integration of multiregional copy number data with or without the addition of point mutations detected by TDS. It also performs subclonal deconvolution to identify several subclones, if present, in each sample [48].

Information about each CNA and SNV were curated into a segment file displaying each genetic alteration in each sample along with its genomic position, type of alteration (gain, loss, CNNI, SNV), the proportion of cancer cells having the alteration in that sample (the mutated clone fraction), and in the case of SNVs, the affected gene and the base pair alteration. Supplementary material, File S1 includes all data used as input to the DEVOLUTION algorithm. For each patient, these data resulted in an event matrix (supplementary material, File S2) that was used for phylogenetic reconstruction using the well‐established methods, maximum parsimony, and maximum likelihood methods separately through the R package phangorn v.2.12.1 [49]. The phylogenies were visualized using the ggplot2 package in R (RRID:SCR_014601) [50]. Phylogenetic trees were reconstructed for CNAs alone for all patients. For patients where TDS data existed, trees were also reconstructed using SNVs alone, as well as with both CNAs and SNVs in the same phylogeny. Supplementary material, Figures [Link], [Link] illustrates all phylogenetic trees together with annotations and interpretations.

### Calculation of the index of genomic diversity

The index of genomic diversity (IGD) was calculated for each sample type in a patient as:

(6) $$\begin{array}{c} \mathrm{IGD}=\frac{\mathrm{dS}1+\mathrm{dS}2+..+\mathrm{dSn}}{\mathrm{dS}1+\mathrm{dS}2+..+\mathrm{dSn}+l\;\times \;N} \end{array}$$

where $dS_{n}$ is the distance from the closest common node for the specific sample type to be investigated, to the subclone n, l is the length of the stem, and *N* is the number of subclones. This has previously been described [32]. The IGD calculations are provided in the supplementary material, File S1D,E.

### Enrichment analysis

For patient GT1, all CNAs with a size <5 Mbp were extracted along with which genes they encompassed. ClusterProfiler (v.4.0.5, RRID:SCR_016884) was used to perform gene set enrichment analysis of the selected genes [51]. As organism database, org.Hg,sgd.db was used. The enrichGO and enrichKEGG functions were used to ascertain whether the affected genes were enriched for certain GO terms or KEGG pathways. A *p*‐value below 0.05 was considered a significant enrichment. The reported *p*‐value for the gseaGO was adjusted using the Benjamini–Hochberg procedure to correct for multiple testing (supplementary material, Figure S7B; File S4).

### COSMIC database

The Catalogue of Somatic Mutations in Cancer (COSMIC) (https://cancer.sanger.ac.uk/cosmic/download/cosmic/v101/completecna) was used to compare small CNAs (<5 Mbp) identified across all samples from patient GT1. This database provides information on somatic mutations associated with human cancers. The CNAs detected in this patient were compared to the COSMIC database, revealing that several of the small CNAs corresponded to known entries in COSMIC.

### Statistical analyses

The Mann–Whitney two‐sided *U*‐test was used for generating the *p*‐values in supplementary material, Figure S3. For paired testing of the IGD between the primary tumor and distant metastases and lymph node metastases, a paired Student's *t*‐test was used. All input data used to perform the statistical analyses can be found in the supplementary material, File S1C–E. A *p* value of <0.05 was considered statistically significant.

## Results

### Extensive genetic heterogeneity between and within primary tumors and metastases

**Figure 2:**
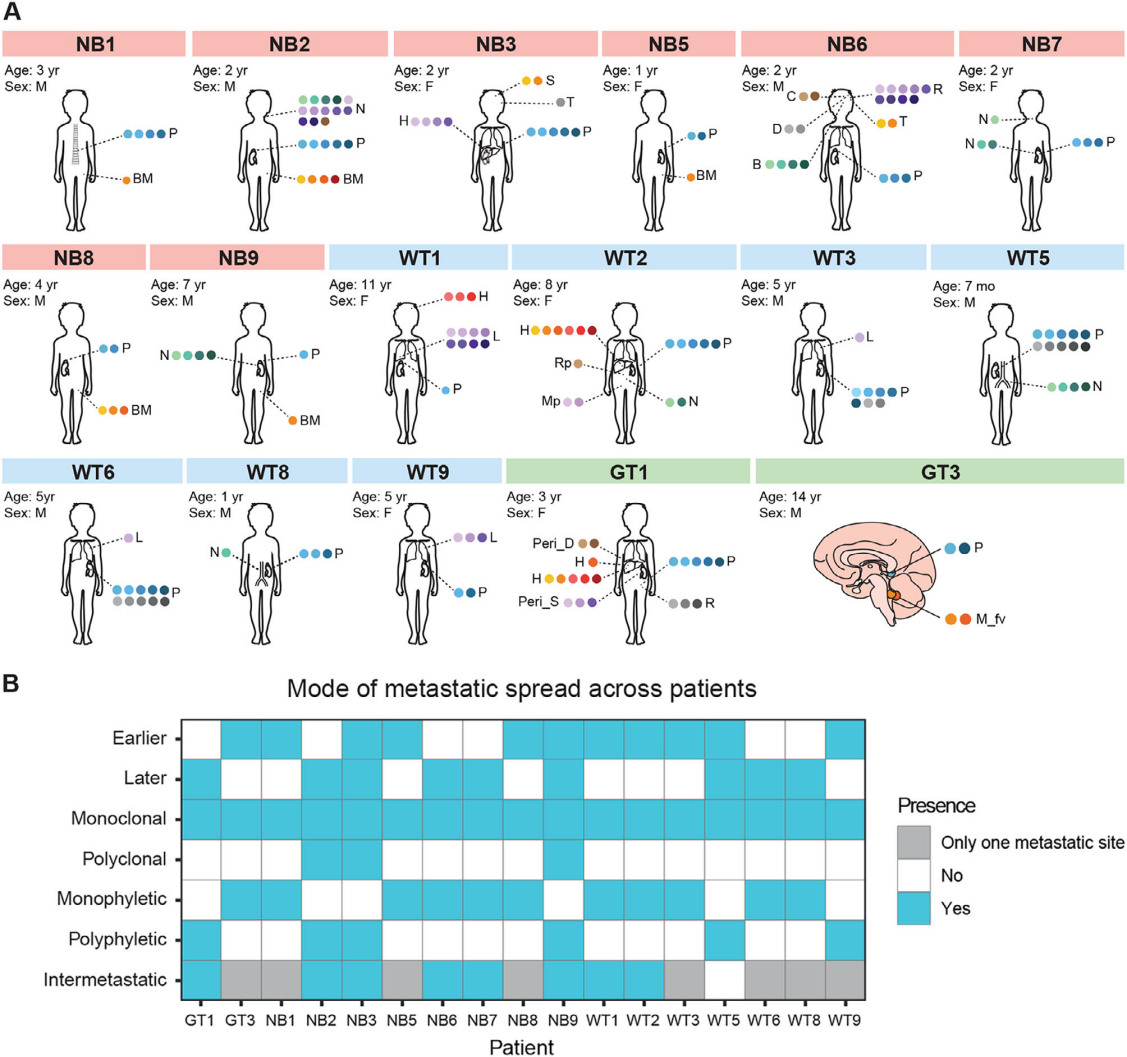
Overview of the patient cohort included in this study. (A) There were eight patients with neuroblastoma (NB), seven with Wilms tumor (WT), and two with gonadal tumors (GTs). Each colored circle represents a sample. The sample types are the primary tumor (P), bone marrow (BM), lung (L), lymph nodes (N), subdural metastasis (S), temporal metastasis (T), the cortex (C), the dura mater (D), the brain (B), relapse (R), pituitary gland (Pi), liver (H), peritoneum close to the diaphragm (peri_D), peritoneum close to the spleen (peri_S), the retroperitoneum (Rp), minor pelvis (Mp), and fourth ventricle (M_fv). For each patient there is also information about the age in years and the genetic sex, where F is female and M is male. (B) Mode of metastatic spread. On the *x*‐axis all included patients can be seen and, on the *y*‐axis, the different modes of metastatic spread investigated as part of this study. A blue box indicates the presence, while a white box indicates the absence of that mode of spread in the corresponding patient. A gray box indicates that there were merely metastases in a single anatomical location and intermetastatic spread can therefore, by definition, not be evaluated.

Patients were selected from a single center consecutive cohort of pediatric solid tumors diagnosed 2000–2020. All patients with NB, WT, or GT were screened for the availability of multiple formalin‐fixed paraffin‐embedded tumor sections from both primary tumor and metastatic sites. The study encompassed 171 samples, consisting of 70 samples from primary tumors and 101 samples from metastases, obtained from 17 patients, with sufficient DNA for genomic analyses. This included a median of eight samples (range 3–22) per patient, with a median of three samples (range 1–10) from the primary tumor and three samples (range 1–19) from metastases per patient (supplementary material, File S1A). Figure 2A provides an overview of the patient cohort.

The two GTs exhibited significantly higher numbers of total, clonal, and subclonal CNAs compared to NB and WT samples (supplementary material, Figure S3A,B). While the NB and WT samples displayed CNA levels of similar magnitudes, some WT samples were identified as outliers (outside 1.5 interquartile ranges below the 1st quartile or above the 3rd quartile) with a high number of genetic alterations (supplementary material, Figure S3A–C). These samples encompassed patients WT3 and WT6, both having diffuse anaplastic WT, a subtype known to often harbor a high number of genetic alterations [52]. There was no statistically significant difference between the total number of CNAs between the distant, lymph node, primary tumor, and relapse samples (supplementary material, Figure S3D). Distant metastases did, however, have a significantly higher number of clonal genetic alterations compared to the primary tumor samples, but not the lymph node metastases (supplementary material, Figure S3E). There was no statistically significant difference for subclonal alterations (supplementary material, Figure S3F).

The index of genomic diversity (IGD) was computed, as described in the Methods section, for each primary tumor and metastatic site individually, revealing considerable variability both within and between primary tumors and metastases (supplementary material, Figure S3G, supplementary material, Files S1 and S2). Strikingly, distant metastases displayed a significantly lower IGD compared to primary tumors (*p* value 0.01). This observation, together with the finding that distant metastases had a significantly higher number of clonal CNAs compared to the primary tumor, implies a strict bottleneck for distant colonization. Conversely, lymph node metastases exhibited a trend toward higher IGD and more subclonal CNAs compared to distant metastases and primary tumors, suggesting a weaker bottleneck for lymph node colonization. Moreover, metastatic samples harbored additional private genetic alterations not detected in the primary tumor in 15 out of 17 patients (supplementary material, Figure S4 and Files S1A–AL, S2A–Q).

Gain of 17q segments and *MYCN* amplification are two of the most common genetic alterations in high‐risk NB [5, 53]. As anticipated, all eight NB patients in this study, only encompassing patients with metastatic disease, displayed at least one variant of 17q gain (supplementary material, Figures S5–S12). In total, seven out of eight NB patients displayed a 17q gain in the stem, four out of eight patients there was one in the branches, and in six out of eight patients all metastases had at least one gain of 17q. Two NB patients had a truncal *MYCN* amplification (supplementary material, Figures S7 and S9: patients NB3 and NB6). Both patients had widespread metastases at several different anatomical sites. Across the WT patients, an 11q copy number neutral imbalance (CNNI) was seen in three patients (supplementary material, Figures S13, S14, S17: patients WT1, WT2, WT6), all of whom were subject to metastatic relapses. Finally, studies have shown that all juvenile granulosa cell tumors either have an activating mutation or a duplication of the *AKT1* gene on 14q [54]. Patient GT1 in this study displayed a gain of the 14q segment, thus confirming this observation (supplementary material, Figure S20). No recurrent genetic alterations across patients directly linked to metastatic spread could be identified, consistent with previous studies (supplementary material, Files S1A‐AL, S2A–Q) [55, 56]. This implies that the acquisition of metastatic capability likely occurs through different pathways, possibly involving convergent evolution and alterations at the level of transcriptional regulation or via microenvironmental alterations.

To conclude, considerable genetic heterogeneity was observed within primary tumors, between primary tumors, and metastases, and among different metastases within the same patient.

### Earlier and later dissemination

In this study we defined earlier spread as metastatic dissemination preceding the emergence of the most recent common ancestor (MRCA) in the primary tumor. Conversely, later metastatic spread was defined as spread after the appearance of the MRCA in the primary tumor. Metastases may harbor subclones disseminated at both earlier and later stages, with some originating as early evolutionary events within the primary tumor, yet disseminating late.

Employing phylogenetic analysis, we unraveled the temporal origins of metastases in each patient. By mapping the position of subclones within phylogenetic trees, we dichotomized metastatic subclones as either earlier or later colonizers of the metastatic site (Figure 2B; supplementary material, File S1B, and Figures S4–S20 illustrate fully annotated trees).

**Figure 3:**
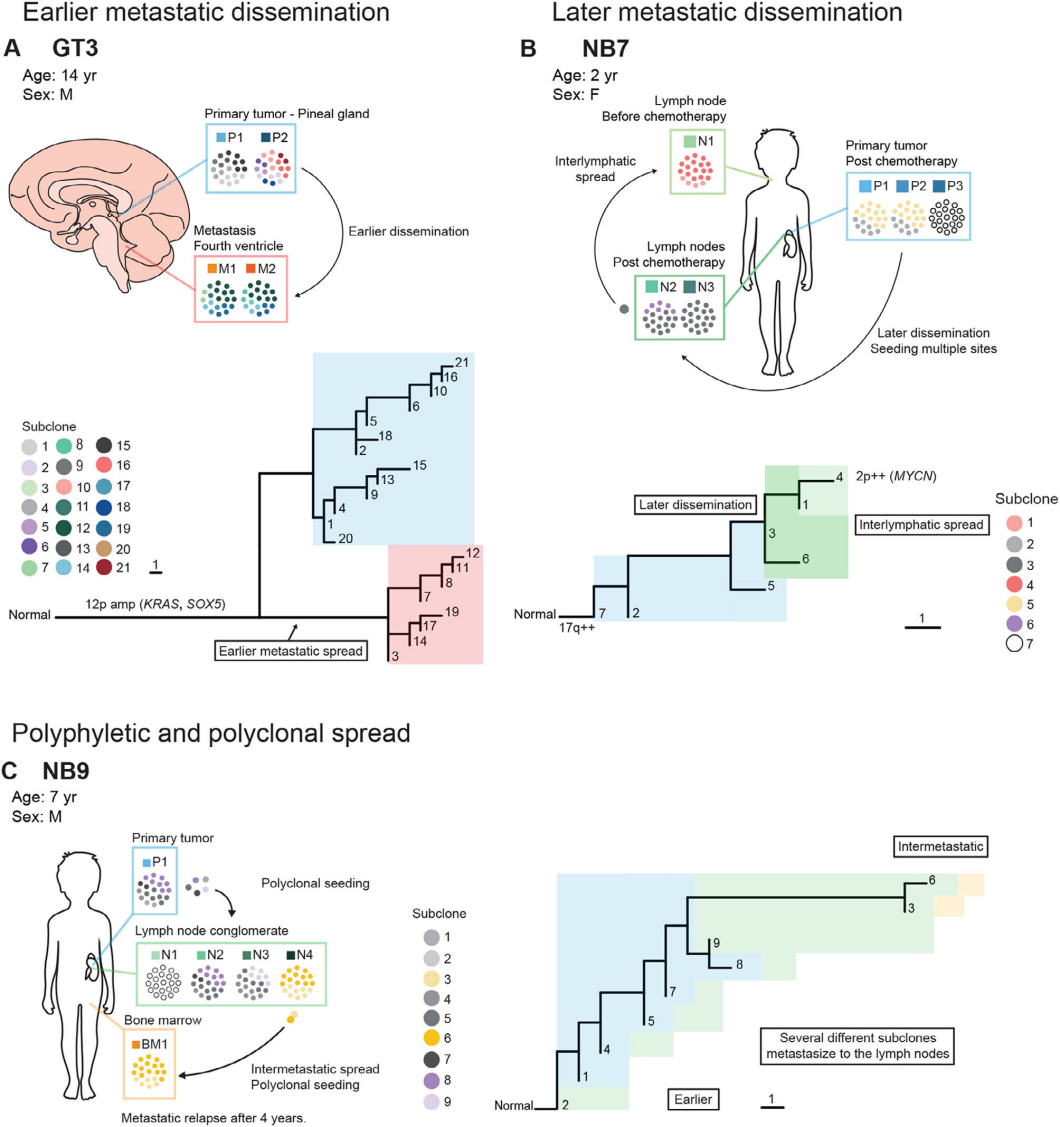
Examples of earlier, later, polyphyletic, and polyclonal spread. (A) Example of earlier metastatic spread: A patient having a GT occurring in the brain. There are two samples from the primary tumor displaying a vast number of subclones. The patient later had a metastatic relapse in the fourth ventricle in the brain. There are two samples from the metastasis (M1 and M2). The subclonal landscape in the metastases are vastly different from the one in the primary tumor. The metastasizing subclone is no longer detectable in the primary tumor, suggesting earlier metastatic spread, possibly already before diagnosis. (B) Example of later metastatic spread: An NB patient presenting with a lymph node metastasis (N1) at diagnosis and a primary tumor located in the left adrenal gland (P1–P3). The patient also presented with metastases in the locoregional lymph nodes after chemotherapy. The lymph node metastases arose later in the evolutionary history of the tumor. (C) Example of polyphyletic and polyclonal spread: An NB patient presenting with a primary tumor in the left adrenal gland (P1), locoregional lymph node metastases (N1–4), as well as a bone marrow metastasis (BM1). Several different subclones, also found in the primary tumor, are found in the lymph nodes as well. In N4, there are two subclones having a vast number of additional genetic aberrations. These subclones are also found in the bone marrow, suggesting both polyclonal spread as well as intermetastatic spread.

In 11 out of 17 patients, earlier metastatic spread was identified (Figure 2B; supplementary material, File S1B; supplementary material, Figures S5, S6, S8, S11–16, S19, S21: patients NB1, NB3, NB5, NB8, NB9, WT1, WT2, WT3, WT5, WT9, and GT3, respectively). In three out of nine patients presenting with metastases in at least two different sites, there were instances of both earlier and later metastatic spread in the same patient (supplementary material, Figures S6, S12, and S16: patients NB3, NB9, WT5). An example of earlier metastatic spread can be seen in patient GT3 (Figure 3A, supplementary material, Figure S21 and supplementary material, Files S1 and S2). This illustrates a patient with a germ cell tumor in the brain. The primary tumor samples (P1 and P2) displayed several different subclones. The patient was treated and later had a metastatic relapse (M1 and M2). At relapse, the subclonal landscape was different compared to diagnosis, a pattern previously coined collateral clonal replacement [32, 57]. No subclone identified at diagnosis was found in the metastasis, indicating that it arose earlier in the evolutionary history of the tumor. We suspect the metastasis may even have been present at diagnosis, but too small to be detectable with radiological methods.

Collectively, 9 out of 17 patients presented evidence of later metastatic spread (supplementary material, File S1B; supplementary material, Figures S6, S7, S9, S10, S12, S16, S17, S18, S20: patients NB2, NB3, NB6, NB7, NB9, WT5, WT6, WT8, and GT1, respectively). Figure 3B shows a neuroblastoma patient (NB7) with a primary tumor located in the left adrenal gland (P1–P3), with a lymph node metastasis in the neck at diagnosis (N1) and locoregional lymph node metastases (N2, N3) after chemotherapy. The lymph node metastases arose later in the evolutionary history of the tumor. Interestingly, the lymph node metastasis sampled at diagnosis harbored an *MYCN* gain of four copies, not detected in the primary tumor or locoregional lymph nodes after chemotherapy. This suggests that the *MYCN* gained cells in the primary tumor, either were depleted by the chemotherapy, or that the *MYCN* gain occurred locally in the distant metastasis. More details can be seen in the supplementary material, Figure S10 and supplementary material, Files S1 and S2.

### Different subclones in the same tumor possess metastatic capability and can colonize the same distant site

Metastases may arise from either a single subclone (monoclonal spread) or if several subclones colonize the same site (polyclonal spread) either arriving simultaneously or sequentially. Monophyletic spread entails a single branch leading to all metastatic subclones, whereas for polyphyletic spread different branches in the phylogeny contribute to the metastases.

Polyclonal seeding was seen in 3 out of 17 patients (supplementary material, Figures S6, S7, S12: patients NB2, NB3, and NB9, respectively) and occurred exclusively in tumors having polyphyletic seeding. Conversely, monoclonal seeding was seen in all 17 patients (Figure 2B; supplementary material, File S1B, and Figures [Link], [Link]), as expected. We found that 6 out of 17 patients displayed polyphyletic seeding (supplementary material, Figures S6, S7, S12, S16, S19, S20: patients NB2, NB3, NB9, WT5, WT9, and GT1, respectively) and 11 out of 17 displayed monophyletic seeding (supplementary material, Figures S5, S8, S9, S10, S11, S13, S14, S15, S17, S18, S21: patients NB1, NB5, NB6, NB7, NB8, WT1, WT2, WT3, WT6, WT8, and GT3, respectively). Polyphyletic seeding was seen in five out of nine patients having metastases in at least two different sites (supplementary material, Figures S6, S7, S12, S16, S20: patients NB2, NB3, NB9, WT5, and GT1, respectively).

Figure 3C illustrates an example of an NB patient (NB9) displaying both polyphyletic and polyclonal seeding (supplementary material, Figure S12 and supplementary material, Files S1 and S2). Several subclones from the primary tumor (P1) spread repeatedly to the lymph nodes (N1–N4), again suggesting a weak bottleneck for lymphatic spread. Notably, in one of the lymph nodes (N4), two subclones with several additional genetic alterations were developed. This patient had a metastatic relapse in the bone marrow 4 years after diagnosis. Strikingly, both subclones found in the bone marrow were present in lymph node sample N4 at diagnosis, suggesting polyclonal seeding from that lymph node to the bone marrow, followed by the cells staying dormant for 4 years before causing the relapse. The precise travel mode of the two subclones could not be determined, but their similar relative prevalence in both the lymph node and the bone marrow suggests that they arrived simultaneously at the bone marrow.

To conclude, several different subclones within a primary tumor can possess metastatic capability and can colonize the same distant site, either sequentially or as tumor cell clusters.

### Intermetastatic spread is a common feature in pediatric cancers

We sought to investigate the presence of intermetastatic spread, defined as subclones spreading between different metastatic sites. Strikingly, dissemination of cancer cells from one metastatic site to another could be identified in eight out of nine patients having metastases in at least two distinct distant sites (Figure 2B, supplementary material, Figures S6, S7, S9, S10, S12, S13, S14, S20, and Files S1 and S2: patients NB2, NB3, NB6, NB7, NB9, WT1, WT2, and GT1, respectively).

**Figure 4:**
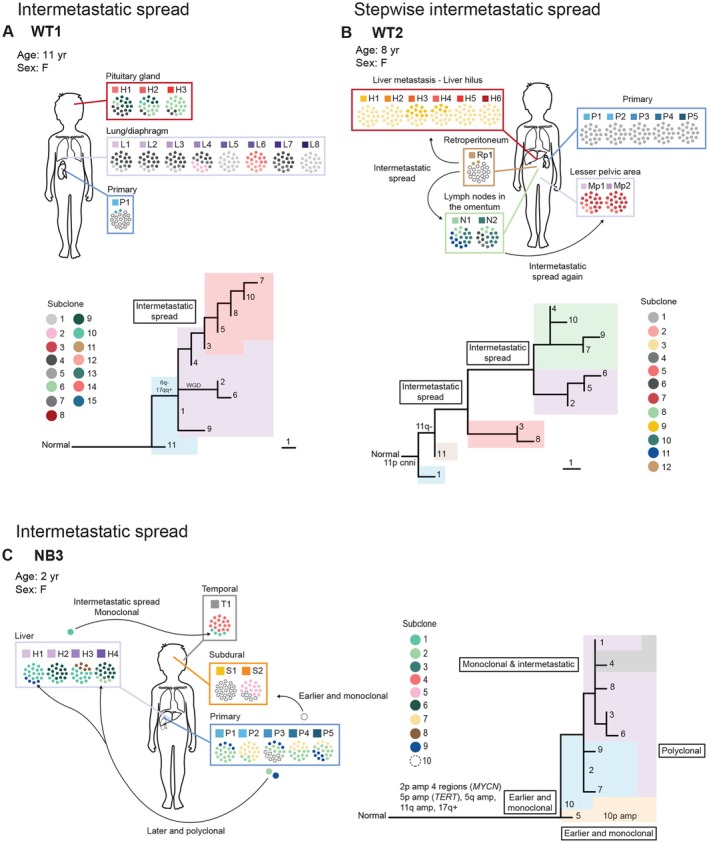
Examples of intermetastatic spread. (A) WT patient presenting with a primary tumor in the right kidney (P1) and an early metastasis in the lung (P1–8). The lung metastasis harbors several different subclones. One of the subclones is also identified in a metastasis in the pituitary gland (H1–3) in the brain (subclone 3), suggesting intermetastatic spread from the lung to the brain. (B) Another WT patient presenting with a primary tumor in the left kidney (P1–5). The patient had an early metastatic spread to the retroperitoneum (Rp1). A minor subclone in Rp1 (subclone 11) is the mother subclone of all subsequent metastases in the liver (H1–6), lymph nodes in the omentum (N1–2), and the minor pelvic area (Mp1–2). There is also an intermetastatic spread between the lymph nodes in the omentum and the minor pelvic area, suggesting that intermetastatic spread can occur stepwise. (C) NB patient presenting with a primary tumor in the right adrenal gland (P1–5). The patient had an earlier spread to the subdural space in the brain (S1–2). There is also a later and polyclonal spread to the liver (H1–4). One of the subclones in the liver metastasis (subclone 1) is also found temporally (extracranially), implying intermetastatic spread.

Figure 4 provides two illustrative examples. In patient WT1 (Figure 4A, supplementary material, Figure S13, and Files S1 and S2), the primary Wilms tumor in the kidney was rather homogenous, with two different subclones, one having the alterations encompassed by the stem only, and the other having an additional deletion, encompassing chromosome 18. The patient presented with an earlier spread to the lung, where a genetically complex metastasis was formed with a total of six different subclones, and where one region also had gone through a whole‐genome doubling event. The patient later developed a brain (pituitary gland) metastasis, which was a descendant of subclone 3, detected in several areas in the lung metastasis, indicating intermetastatic spread from the lung to the brain.

Another example of intermetastatic spread is seen in patient WT2 (Figure 4B, supplementary material, Figure S14, and Files S1 and S2), with an earlier metastatic spread from the primary WT in the kidney to the retroperitoneum. Interestingly, all other metastases found in the patient originated from subclone 11 in this earlier metastasis. Both intermetastatic spread and monophyletic spread were detected. Strikingly, the metastasis in the minor pelvic region seemed to originate from the lymph nodes in the omentum. In this patient, intermetastatic spread thus probably occurred twice: from the retroperitoneum to the lymph nodes in the omentum and from the lymph nodes in the omentum to the minor pelvic region. The metastasis in the retroperitoneum thus acted as a hub for further spread.

Finally, another example of intermetastatic spread was detected in the NB patient NB3 presenting with a primary tumor (P1–P5) in the right adrenal gland. The patient displayed an earlier monoclonal spread to the subdural cavity in the brain as well as a later polyclonal spread to the liver. From the liver there was an intermetastatic spread to the left temporal area (extracranially). In this patient there was hence both earlier and later spread, monoclonal and polyclonal spread, as well as intermetastatic spread (Figure 4C, supplementary material, Figure S7, and Files S1 and S2). In summary, intermetastatic spread emerges as a recurrent phenomenon across pediatric tumors.

### Targeted deep sequencing confirms the observed metastatic trajectories

**Figure 5:**
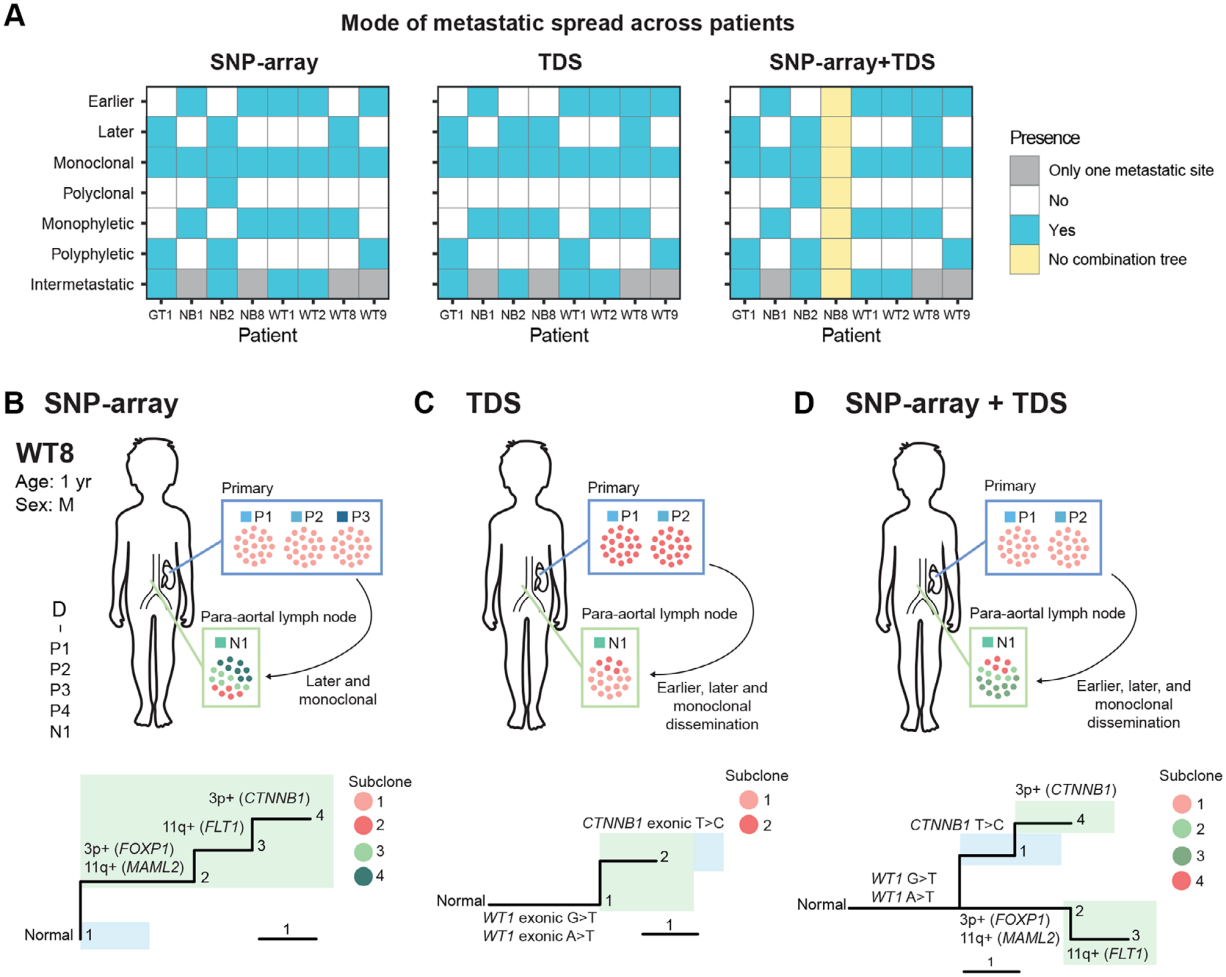
Analysis of the TDS data. (A) Mode of metastatic spread. On the *x*‐axis all included patients can be seen and, on the y‐axis, the different modes of metastatic spread investigated as part of this study. A blue box indicates the presence, while a white box indicates the absence of that mode of spread in the corresponding patient. A gray box indicates that there were only metastases in a single anatomical location and intermetastatic spread can therefore, by definition, not be evaluated. A yellow box indicates that the combination phylogeny could not be generated. (B) The patient presented with a primary tumor in the left kidney (P1–3). The patient also had a lymph node metastasis para‐aortally (N1). The primary tumor displayed no CNAs, while the lymph node metastasis displayed four CNAs. (C) TDS analysis revealed two clonal exonic nonsynonomous SNVs of the *WT1* gene in all samples, and a subclonal exonic nonsynonomous SNV of the *CTNNB1* gene in all samples. (D) By combining the SNP‐array and TDS data, a more complete picture could be obtained of this patient's tumor.

We performed TDS on 64 samples from eight patients (NB1, NB2, NB8, WT1, WT2, WT8, WT9, GT1) with a median of eight samples per patient, of which there was a median of two samples from the primary tumor (range 1–8) and five samples from the metastases (range 1–10) (supplementary material, File S1A). Phylogenetic trees were reconstructed based on the TDS data alone, as well as by combining the CNA data and TDS data. The phylogenetic trees based on the TDS data alone were less branched compared to the CNA trees due to the lower number of SNVs compared to CNAs (supplementary material, Figures S22–S40, and File S1). The phylogenetic trees confirmed the metastatic trajectories, as ascertained using whole‐genome copy number data (Figure 5A, supplementary material, Figures S22–S40, and File S1B). Notably, all four patients having metastases in at least two different sites showed evidence of intermetastatic spread also by TDS (Figure 5A, supplementary material, File S1B, supplementary material, Figures S6, S13, S14, S20: patients NB2, WT1, WT2, and GT1, respectively).

The TDS analysis did, however, in some cases, provide additional information important for a deepened understanding of the pathogenesis of each patient's tumor. One example is patient WT8, for whom there were three samples from the primary tumor (P1–P3) and one from a para‐aortal lymph node (N1), all sampled at diagnosis. The primary tumor did not harbor any CNAs, while the lymph node metastasis harbored four CNAs (Figure 5B). Interestingly, TDS analysis revealed that all samples, including the diploid primary tumor samples, exhibited two different clonal exonic nonsynonomous mutations of the *WT1* gene, and a subclonal exonic nonsynonomous mutation of the *CTNNB1* gene, both known to be important for WT pathogenesis (Figure 5C) [58]. By integrating the CNA and TDS data, a more comprehensive description of the patient's tumor could thus be obtained (Figure 5D).

Another example is the Wilms tumor patient WT1, harboring a primary tumor in the right kidney and who later had intermetastatic spread from a lung metastasis to the brain. There were two distinct exonic nonsynonomous mutations of the *ATM* gene in the stem, which is one of the most frequently altered genes in breast cancer brain metastases [59]. It does, however, remain to be investigated whether *ATM* gene mutations are a recurrent feature in brain metastases in WT as well.

Wilms tumor patient WT2 (supplementary material, Figures S14, S31, and S33) displayed five different exonic synonomous mutations of the *ESR1* gene across various parts of the primary tumor, none of which were detectable in the metastases. Interestingly, this patient only had a single CNA in the stem, a 11p cnni (copy number neutral imbalance), encompassing the *WT1* locus, but no *WT1* mutation could be detected. Two metastases, one in the lesser pelvic area and one in the lymph nodes of the omentum, displayed an intronic androgen receptor (*AR*) mutation (supplementary material, Figure S31). There are several putative WT1 protein binding sites in the *AR* promoter [60] to where WT1 can bind and alter the promoter activity and expression of the *AR* gene [61]. Possibly these alterations collectively contributed to the pathogenesis of the disease in this patient.

In patient WT9 (supplementary material, Figure S35), mutations of *RB1* (intronic), *IGF1R* (intronic), *BLM* (exonic nonsynonomous) and *WT1* (exonic stopgain), all genes with importance for WT pathogenesis, were identified in the primary tumor, but not in any of the samples from the lung metastasis. Interestingly, *BLM* has been identified as a WT predisposition gene and may thus play a role in driving tumor evolution [62]. One area in the primary tumor as well as the lung metastasis had a mutation of the *DICER1* gene, implicated in familial WT [63]. The patient also had a WGD in these two tumor areas followed by an additional gain of the *DICER1* gene segment in the lung metastasis (supplementary material, Figure S36).

Patient NB1 displayed several gains of genes related to the core regulatory circuit in NB [64, 65], possibly driving the disease. This patient had at least one extra copy of *TWIST*, *TRIM21*, *LMO1*, *FOSL1*, *TBX2*, *ISL1*, and *HAND1* [65]. The bone marrow metastasis had no point mutations, but an *FAT1* deletion, shown to promote stemness, metastasis, and induction of a hybrid EMT state in other cancer types [66]. Patient NB2 (supplementary material, Figure S24) exhibited mutations in *NTRK3* (intronic), *FANCA* (exonic nonsynonomous) and in two different sites of the *BRCA1* (intronic) gene. Similarly, patient NB8 (supplementary material, Figure S27) presented with mutations in *NTRK1* (intronic), *NTRK3* (exonic synonomous), *FANCA* (exonic nonsynonymous) and *BRCA1* (exonic nonsynonymous). *NTRK1* and *NTRK3* are important for neural crest and sympathoadrenal development as well as in neuroblastoma cells, where they induce differentiation [67]. Additionally, *FANCA* and *BRCA1* have been shown to increase the risk of developing NB [68].

Lastly, patient GT1 displayed a complex and heterogenous CNA landscape, while merely displaying two mutations in two different metastases, encompassing the genes *RBM10* (exonic nonsynonomous) and *AR* (exonic nonsynonomous) (supplementary material, Figures S37 and S38).

In conclusion, the TDS data confirmed the metastatic trajectories as ascertained using CNA data. By combining the two datasets, a more comprehensive understanding of the patients' diseases could be obtained.

### Genetic alterations across metastases

We sought to identify the distinguishing characteristics in the copy number and TDS data across the three tumor types. Specifically, we analyzed copy number profiles for individual samples from each patient (supplementary material, Figure S42).

We generated aggregated heatmaps for all primary tumor and metastasis samples, both with and without background ploidy correction (supplementary material, Figure S43A–D). These heatmaps reveal recurrent alterations of chromosomal segments, such as 2p and 17q, which are frequently seen in neuroblastomas; otherwise, no clear shared genetic aberrations emerged [5, 53].

In addition, we identified both CNAs and SNVs unique to metastases, defined as alterations absent in the corresponding primary tumors (supplementary material, Figures S44A,B, and S45). Yet again, no shared genetic alterations were observed across patients. Using open cravat, we also analyzed all point mutations across all sample and found that several ones, identified both in the primary tumor and metastases, have clinical relevance (supplementary material, File S6) [69].

In summary, no common alterations were identified that could explain metastatic spread across patients (supplementary material, Figures S42–S45 and Files S1A–AL, S2A–Q, S6).

### Multiregional sampling unravels amplicon spatiotemporal heterogeneity

Amplification of oncogenes is well documented in cancer pathogenesis [70, 71]. Through comprehensive multiregional sampling encompassing primary tumors and metastases, we shed light on the spatiotemporal dynamics of amplified chromosomal segments.

Our analysis identified amplified regions, characterized by the presence of more than eight copies, in five out of 17 patients (supplementary material, Figures S7, S9, S10, S20, S21: patients NB3, NB6, NB7, GT1, and GT3, respectively) (supplementary material, Files S1–S3). Notably, four out of these five patients displayed at least one truncal amplification (NB2, NB6, GT1, GT3), signifying a potential role in tumor initiation. Patient GT3 had a truncal amplification on 12p including *KRAS* and *SOX5* (supplementary material, Figure S21 and File S2). Amplification of the *KRAS* oncogene is a recurrent feature of intracranial germ cell tumors, detected in 15% of patients [72]. Moreover, two NB patients displayed a truncal amplification of the *MYCN* oncogene (supplementary material, Figures S7 and S9: patients NB3 and NB6, respectively) [64].

Interestingly, multiregional and temporal sampling revealed subclonal amplifications in patients NB3, NB7, and GT1. Patient NB7 (supplementary material, Figure S10: patient NB7) had a subclonal *MYCN*‐amplification, identified in a single lymph node metastasis in the neck at diagnosis. Notably, despite extensive sampling, this aberration was absent in locoregional lymph nodes and the primary tumor. In patient NB3, an amplicon on 10p14 was only detected in the subdural metastasis, and encompasses the gene *GATA3*, known to be part of the NB core regulatory circuit [65]. Hence, amplifications can occur both earlier and later in the evolutionary history of the tumor.

Moreover, Patient NB3 presented with a primary tumor in the right adrenal gland, followed by metastatic spread both to the subdural space around the brain, and to the liver separately. The patient later had intermetastatic spread from the liver to the subcutaneous tissue of the temporal area. Strikingly, this patient displayed nine different amplified areas, eight of which were truncal, including *SOX11* [73], *MYCN*, *SDC1* [64], and *TERT* [74] (supplementary material, File S2 sheet E): patient NB3 (supplementary material, Figure S7B and File S3). The truncal amplification on 5q22.2 did not encompass any known oncogene, but it did affect the gene *MCC*, which has been shown to be a tumor suppressor in several other cancer forms [75]. The amplicon splits this gene in half, making the protein product nonfunctional. In addition, there is a loss of the other allele. Moreover, the 11q13.3–13.4 segment encompasses *SHANK2*, shown to act as a tumor suppressor in NB cells [76]. Interestingly, also this amplicon splits this tumor suppressor gene in half, making it nonfunctional. There may, however, still be a functional copy in the other chromosome. In this case, there was a loss covering *SHANK2*, possibly resulting in a complete loss‐of‐function of this gene.

In conclusion, by utilizing multiregional sampling encompassing both primary tumors and metastases, we were able to dichotomize amplifications into two distinct categories: those occurring early, possibly also during tumor initiation, and those emerging later, influencing tumor progression. Our analysis revealed that while some amplifications led to additional copies of complete oncogenes, we also uncovered a novel phenomenon: amplicon‐mediated cleavage of tumor suppressor genes, a previously undocumented mechanism for tumor suppressor gene inactivation.

### Unraveling the genomic complexity of metastatic juvenile granulosa cell tumors: insights from patient GT1

Patient GT1 presents a rare case of juvenile granulosa cell tumor. It is typically associated with a favorable prognosis [77], yet this patient exhibited an atypical course marked by relapse and widespread metastatic dissemination. Employing a multifaceted approach, we scrutinized the evolutionary history of this patient's tumor, leveraging a comprehensive dataset comprising 19 samples spanning primary and metastatic sites to unravel the evolutionary trajectories that underly this atypical clinical presentation.

Targeted deep sequencing did not reveal any truncal somatic mutations in this patient. There were merely two different point mutations detected in the peritoneal metastasis by the diaphragm (supplementary material, Figure S37). In contrast, analysis of copy number alterations revealed a complex landscape with 135 unique CNAs identified across samples. Intriguingly, tracking subclonal dynamics through copy number alterations delineated instances of later metastatic spread, occurring several times during tumor evolution, and being caused by several different subclones. There were also instances of polyclonal spread and intermetastatic spread from the liver metastases to two different parts of the peritoneum.

**Figure 6:**
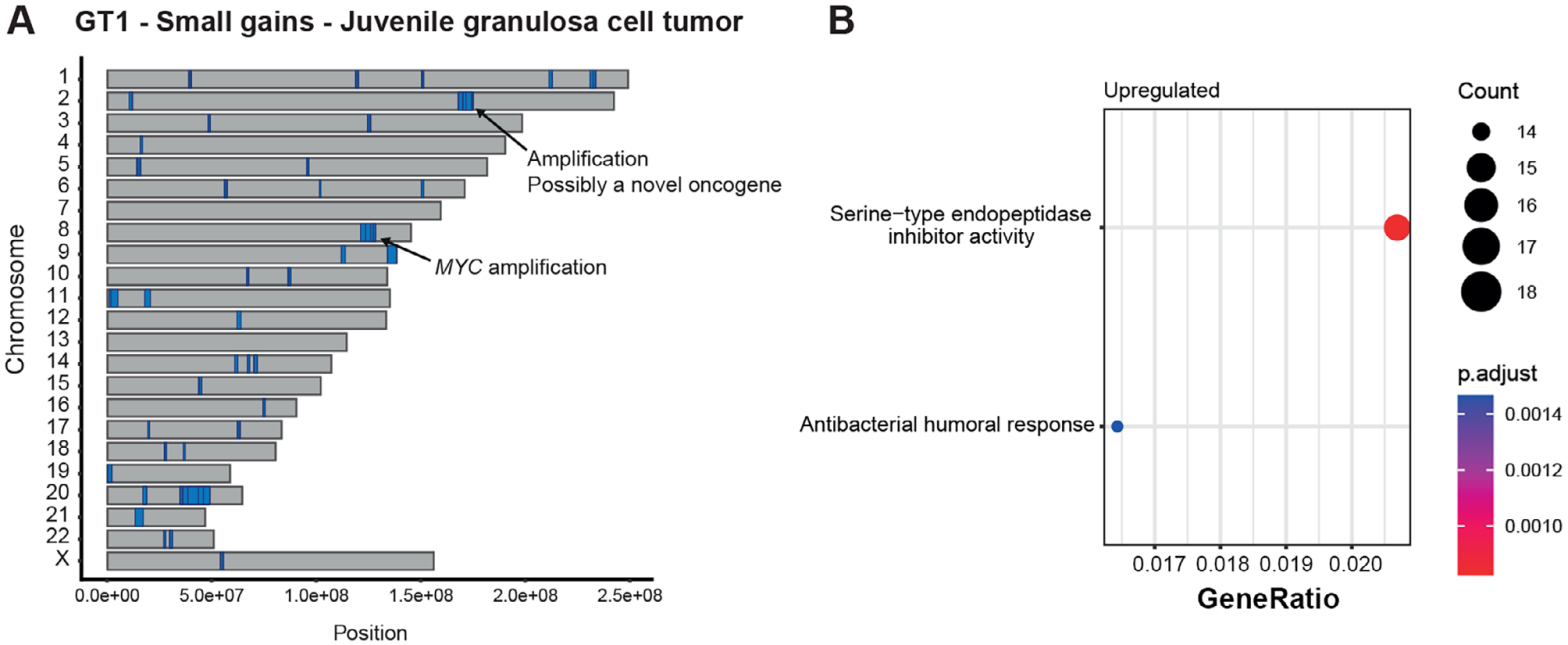
Duplication signature in gonadal tumor patient GT1. (A) On the *y*‐axis each chromosome is denoted and, on the *x*‐axis, the genomic position. Leftmost indicates the p‐arm and the rightmost the q‐arm. Each blue segment represents a gain <5 Mbp observed across samples. Arrows point at an MYC amplification and an amplification of 2q, not containing any known oncogene. (B) These small duplications were significantly enriched for serine‐type endopeptidase inhibitor activity.

Upon closer examination this patient's tumor was found to have several small gains <5 Mbp in size, detected across all samples, including both the primary tumor and metastases (Figure 6A). These were found to be associated with known copy number variations (CNVs) implicated in various cancer types (supplementary material, Figure S46, and File S7). To decipher the functional implications of these duplications, we conducted gene set enrichment analysis focusing on the affected genes. We observed a significant association with upregulation of serine‐type endopeptidase inhibitor activity, encompassing a total of 18 of the genes within the duplication signature (Figure 6B, supplementary material, File S4A). Moreover, a total of 233 genes on lost segments were linked to serine metabolism (supplementary material, File S4B,C). Given the well‐established role of serine metabolism in cancer progression and poor prognosis [78], particularly in the context of metastasis formation, this enrichment pattern suggests a potential mechanism driving tumor aggressiveness in this patient.

Previous studies have demonstrated that *AKT1* duplications or mutations are present in all juvenile granulosa cell tumors [54]. In our investigation, the tumor exhibited a segmental gain of the *AKT1* gene in the stem, corroborating these findings and the diagnosis (supplementary material, Figure S20 and File S1F). Notably, 16 of the genes in the duplication signature were related to the PI3K‐AKT1 signaling pathway, known to be pivotal in juvenile granula cell tumor pathogenesis (supplementary material, File S4D) [54]. Expanding our analysis to encompass all gains across samples, a total of 74 affected genes were associated with the PI3K‐AKT1 signaling pathway (supplementary material, File S4E,F), while losses impacted 80 genes (supplementary material, File S4A).

Moreover, there were two amplified genomic regions (supplementary material, Figure S20). One amplification was located on 8q.24, containing the *MYC* oncogene. The other amplification was seen on 2q.31, not containing any known oncogene in juvenile granulosa cell tumors. There is, however, a lack of studies of the CNA landscape of these tumors. It is also now known whether amplifications in this region is a recurrent feature.

Notably, the 2q31.1 amplification preceded the 8q24.21 amplification, suggesting a potential role in tumor initiation. Conversely, the 8q24.21 amplification emerged later in the evolutionary history of the tumor, swept in the primary tumor and were present in all analyzed cells in both the relapse and all metastases. This chronology suggests that while the 2q31.1 amplification may have contributed to tumor initiation, the 8q.24 (*MYC*) amplification likely drove tumor progression rather than initiation. Notably, *MYC* is a known oncogene in other cancer forms [79], implicating its role in the aggressive behavior in this patient.

In conclusion, our study of patient GT1 sheds light on a rare and challenging manifestation of juvenile granulosa cell tumor, characterized by unexpected relapse and widespread metastasis. Owing to the extensive multiregional and temporal sampling, we could show that somatic point mutations were rare, while examination of CNAs revealed a complex and dynamic genomic landscape across different metastatic sites. Notably, we reveal, for the first time, instances of intermetastatic spread in these tumor forms, expanding our understanding of their metastatic behavior. Moreover, our study confirms the presence of *AKT1* duplications in these tumors, while also identifying small duplications impacting genes associated with serine metabolism and the PI3K‐AKT1 pathway, potentially driving tumor aggressiveness. Furthermore, an *MYC* amplification presented in the primary tumor, swept, and was identified in the relapse and all metastases, possibly playing a pivotal role in promoting tumor progression in this patient. Collectively, these insights advance our understanding of tumor evolution and metastasis in juvenile granulosa cell tumors (Figure 6).

## Discussion

Metastatic disease poses a major clinical challenge, with limited curative options, particularly in pediatric tumors where research on metastatic mechanisms has been scarce. Understanding tumor spread is crucial for developing effective treatments. This study uncovers the evolutionary trajectories of metastasis across the pediatric cancers: neuroblastoma, Wilms tumor, and gonadal tumors, revealing key dynamics such as the timing of metastasis, monoclonal *versus* polyclonal spread, and intermetastatic dissemination.

Given the significant intratumoral heterogeneity observed in these tumors [19, 20, 80], there is a risk that subclones believed to be metastasis‐specific, are present in the primary tumor, but elude detection due to inadequate sampling. In this study we tried to minimize this risk by extracting DNA from multiple sites within the primary tumor, as well as from various metastatic sites for each patient. Despite these efforts, metastatic lesions frequently exhibited additional genetic alterations not identified in the primary tumor. Notably, some of these alterations were subclonal and ancestral to metastasis‐specific alterations, further supporting their metastatic origin. Collectively, this underscores the importance of sampling metastases, if feasible, before initiating molecularly targeted therapies, since the metastases may lack the drug target [81]. Such variations could lead to disease progression at distant sites, despite a positive response to treatment in the primary tumor. Thus, integrating multiregional genotyping into clinical practice is essential for optimizing treatment strategies and improving patient outcomes.

Metastases can emerge at various points along the evolutionary timeline of the tumor, with instances of both earlier and later metastatic events observed in this study. Sometimes, earlier and later metastatic spread was seen in the same patient. Interestingly, monoclonal dissemination emerged as the predominant mode of spread, detected across all patients. A subset of patients exhibited evidence of both polyclonal and polyphyletic seeding. This suggests that metastatic dissemination may occur multiple times during tumor evolution, emphasizing the remarkable diversity of subclones possessing the capability to metastasize. Furthermore, the observation that distinct subclones can independently colonize the same metastatic site adds another layer of complexity to the metastatic process.

A higher number of subclones from the primary tumor metastasized to the lymph nodes compared to distant sites, suggesting a weaker evolutionary bottleneck for locoregional lymphatic spread and a stricter one for colonizing distant sites. A similar pattern has been observed in colorectal cancer [82]. This begs the question if lymph nodes serve as facilitators of dissemination or merely as indicators of metastatic potential, by simply catching various subclones entering their interior. Interestingly, a few studies in some adult cancer suggest that removing the locoregional lymph nodes have no effect on long‐term survival, but it is likely context‐dependent, since lymph node removal is associated with improved survival in other cancer types [83, 84]. The continuous drainage of lymph from the tumor interior may possibly create a natural conduit for loosely attached tumor cells, leading to their dissemination to the lymph nodes. Hematogenous distant metastatic spread may, instead, require a more active migratory process. Further investigation of these mechanisms using immunohistochemistry or RNA sequencing could allow exploration of the phenotypical differences between subclones found in the locoregional lymph nodes to those in distant sites, possibly offering valuable insights into the metastatic process.

Intermetastatic spread emerged as a prevalent feature across pediatric tumors, being identified in almost all patients having metastases at several metastatic sites. Additionally, intermetastatic spread was observed several times within the same patient. This suggests that new metastases can develop even after removal of the primary tumor. Future studies should explore potential differences in transcriptional states between subclones that participate in intermetastatic spread and those that do not. Furthermore, exploration of targetable mechanisms in these cells may offer insights into how to hinder further metastatic development.

We found no recurrent genetic alteration that could explain metastatic spread, in line with previous studies [55]. To elucidate the distinctive features of metastasizing subclones compared to their nonmetastasizing counterparts within the primary tumor, forthcoming studies could integrate RNA sequencing or immunohistochemistry analyses on matched primary tumor samples and multiple metastases. RNA sequencing data holds promise in unraveling potential transcriptional cell states linked to metastatic spread. Metastasizing cells are thought to undergo an epithelial to mesenchymal transition, implying a mesenchymal‐like state akin to mesenchymal cells implicated in treatment resistance [85, 86]. Additionally, the subclones may undergo mesenchymal‐to‐epithelial transition at the metastatic site [87]. Enhanced metastatic capacity has been observed in mesenchymal cells across various adult cancer [86, 88].

We discovered amplifications in regions devoid of known oncogenes in both patients NB3 and GT1. Moreover, patient NB3 exhibited two instances of amplification in an area with no known oncogene, but where the edge of the amplicon truncated a tumor suppressor gene and there was a loss of the other allele. It is possible that amplification of part of a tumor suppressor gene could lead to its inactivation, through a form of ‘amplification inactivation.’ Gaining one copy of a gene segment could thus mimic the effect of deleting one copy of the gene. However, the prevalence of this phenomenon remains unexplored, and it will require further investigation into whether the amplifications are recurrent features in these tumors.

Patient GT1 presented a compelling clinical conundrum with widespread metastatic disease, despite an initial diagnosis of juvenile granulosa cell tumor. Leveraging a comprehensive collection of 19 samples from multiple regions of the primary tumor and multiple metastatic lesions, we investigated the underlying disease dynamics. Here the metastatic spread occurred at several timepoints after the local relapse. In addition, there were polyclonal spread as well as intermetastatic spread from the liver to the peritoneum. Moreover, this patient displayed a large number of small <5 Mbp CNAs, found in almost all samples in both the primary tumor and all metastatic sites. Noteworthy among these alterations was an amplification of 8q24.2, housing the *MYC* oncogene, alongside an amplification of 2q31 devoid of known oncogenes. Furthermore, these small gains were enriched in genes associated with upregulated serine metabolism. Our findings emphasize the need for multisample analyses to unravel complex cases like Patient GT1, while also highlighting the recurrent CNA signatures in this tumor subtype that merit further molecular investigation.

The size of the patient cohort in this study presents a challenge. NB, WT, and GT are rare malignancies, and most patients affected with WT and GT do not present with metastatic disease. Having a small patient cohort did, however, allow us to adopt a comprehensive approach, extracting DNA from multiple regions of the primary tumor and various metastatic sites from each patient, having a median of eight samples available from each patient. This strategy provided a detailed characterization of the mutational landscape and evolutionary dynamics within each patient. By doing so, we were able to address our primary research questions, focusing on tracking metastatic subclones spatially and temporally, elucidating the timing of metastatic onset, assessing the metastatic potential of various subclones, and investigating whether subclones in metastases can seed new metastatic lesions.

Moreover, our sample cohort exclusively relied on FFPE material, presenting inherent limitations. Extracting high‐quality and quantity DNA from FFPE can be challenging, restricting the scope of genetic analyses feasible. We opted to whole‐genome copy number profiling, enabling high‐resolution copy number identification in each sample. Pediatric tumors typically have very few pathogenic somatic mutations and mainly harbor larger chromosomal aberrations [19]. Thus, this approach offers valuable insights into the subclonal landscape and facilitates tracking of subclonal evolution across space and time, effectively addressing our research objectives. Additionally, TDS up to 10,000× sequencing depth was performed to detect SNVs, further enhancing the resolution of metastatic spread delineation.

Additionally, determining the exact mode of metastasis is inherently challenging due to the complex and dynamic nature of tumor evolution. Given the variability in metastatic patterns and the potential for both clonal and subclonal dissemination, we opted to use phylogenetic methods, which offer a robust and systematic approach to track the evolutionary trajectories of tumor subclones. By constructing phylogenetic trees for each patient, the order of divergence of subclones across the primary tumor and metastatic sites can be determined. This methodology reduces the risk of bias or subjectivity that may arise from less rigorous approaches, ensuring that our findings are based on a comprehensive and objective analysis of the tumor's evolutionary history. Such an approach provides valuable insights into the timing and mechanisms of metastatic spread, facilitating a clearer understanding of how these tumors evolve and metastasize over time.

To conclude, by comprehensively tracking metastatic subclones across space we found that metastatic spread can occur earlier, later, or several times during tumor evolution. Numerous distinct subclones in the primary tumor can have metastatic capability. Metastases often had additional genetic alterations, not detected in the primary tumor, underscoring the importance of sampling metastatic lesions to identify therapeutic targets. Intermetastatic spread is in fact identified as a common feature across these pediatric tumors. This indicates that a metastasis alone may act as a hub for further metastatic spread despite the primary tumor being removed, having important clinical implications.

## Author contributions statement

NA and DG undertook study planning and patient selection. NA and DG performed histological assessment, and MF and CJ performed DNA extraction. NA was responsible for analysis of whole‐genome copy number analysis data, targeted sequencing data, phylogenetic analysis, and creation of all figures. SC performed the theoretical input to bioinformatical TDS analyses. NA, JK and DG interpreted the results. NA wrote the article. All authors actively participated in article revision.

## Supporting information


**Data S1.** Supporting Information Cover page

**Figure S1.** Workflow for somatic mutational analysis
**Figure S2**. Workflow for copy number analysis
**Figure S3**. Dotplots of the number of copy number alterations and the index of genomic diversity across samples and patients
**Figure S4**. Private and shared genetic alterations in each anatomical location
**Figure S5**. Neuroblastoma patient 1: Single nucleotide polymorphism (SNP)‐array data
**Figure S6**. Neuroblastoma patient 2: Single nucleotide polymorphism (SNP)‐array data
**Figure S7**. Neuroblastoma patient 3: Single nucleotide polymorphism (SNP)‐array data
**Figure S8**. Neuroblastoma patient 5: Single nucleotide polymorphism (SNP)‐array data
**Figure S9**. Neuroblastoma patient 6: Single nucleotide polymorphism (SNP)‐array data
**Figure S10**. Neuroblastoma patient 7: Single nucleotide polymorphism (SNP)‐array data
**Figure S11**. Neuroblastoma patient 8: Single nucleotide polymorphism (SNP)‐array data
**Figure S12**. Neuroblastoma patient 9: Single nucleotide polymorphism (SNP)‐array data
**Figure S13**. Wilms tumor patient 1: Single nucleotide polymorphism (SNP)‐array data
**Figure S14**. Wilms tumor patient 2: Single nucleotide polymorphism (SNP)‐array data
**Figure S15**. Wilms tumor patient 3: Single nucleotide polymorphism (SNP)‐array data
**Figure S16**. Wilms tumor patient 5: Single nucleotide polymorphism (SNP)‐array data
**Figure S17**. Wilms tumor patient 6: Single nucleotide polymorphism (SNP)‐array data
**Figure S18**. Wilms tumor patient 8: Single nucleotide polymorphism (SNP)‐array data
**Figure S19**. Wilms tumor patient 9: Single nucleotide polymorphism (SNP)‐array data
**Figure S20**. Gonadal tumor patient 1: Single nucleotide polymorphism (SNP)‐array data

**Figure S21.** Gonadal tumor patient 3: Single nucleotide polymorphism (SNP)‐array data
**Figure S22**. Neuroblastoma patient 1: targeted deep sequencing (TDS) data
**Figure S23**. Neuroblastoma patient 1: targeted deep sequencing (TDS) and single nucleotide polymorphism (SNP)‐array data
**Figure S24**. Neuroblastoma patient 2: targeted deep sequencing (TDS) data
**Figure S25**. Neuroblastoma patient 2: targeted deep sequencing (TDS) data and SNP array data—maximum parsimony (MP) tree
**Figure S26**. Neuroblastoma patient 2: targeted deep sequencing (TDS) data and SNP array data—maximum likelihood (ML) tree
**Figure S27**. Neuroblastoma patient 8: targeted deep sequencing (TDS) data—Maximum parsimony (MP) tree
**Figure S28**. Neuroblastoma patient 8: targeted deep sequencing (TDS) data—Maximum likelihood tree (ML)
**Figure S29**. Wilms tumor patient 1: targeted deep sequencing (TDS) data
**Figure S30**. Wilms tumor patient 1: targeted deep sequencing (TDS) and single nucleotide polymorphism (SNP)‐array data
**Figure S31**. Wilms tumor patient 2: targeted deep sequencing (TDS)
**Figure S32**. Wilms tumor patient 2: targeted deep sequencing (TDS) and single nucleotide polymorphism (SNP)‐array data
**Figure S33**. Wilms tumor patient 8: targeted deep sequencing (TDS) data
**Figure S34**. Wilms tumor patient 8: targeted deep sequencing (TDS) and single nucleotide polymorphism (SNP)‐array data
**Figure S35**. Wilms tumor patient 9: targeted deep sequencing (TDS) data
**Figure S36**. Wilms tumor patient 9: targeted deep sequencing (TDS) and single nucleotide polymorphism (SNP)‐array data
**Figure S37**. Gonadal tumor patient 1: targeted deep sequencing (TDS) data
**Figure S38**. Gonadal tumor patient 1: targeted deep sequencing (TDS) and single nucleotide polymorphism (SNP)‐array data
**Figure S39**. Overview of the metastasis patterns across patients NB1–NB9 and WT1
**Figure S40**. Overview of the metastasis patterns across patients WT2–WT9 and GT1 and GT3

**Figure S41.** Copy number heatmaps

**Figure S42.** Heatmap of all copy number alterations across all samples and patients
**Figure S43**. Heatmap of the sum of copy number alterations across all samples and patients
**Figure S44**. Heatmap of all metastasis unique copy number alterations across all metastasis samples
**Figure S45**. Heatmap of all metastasis unique single nucleotide variants across all metastasis samples.
**Figure S46**. Duplication signature in gonadal tumor patient GT1

**File S1.** Patient information, mode of spread, IGD calculations and all CNAs and SNVs for each sample in each patient

**File S2.** All output matrices from DEVOLUTION

**File S3.** Amplifications

**File S4.** Enrichment results

**File S5.** TDS panel information

**File S6.** CNAs shared between patients and unique for metastases

**File S7.** Analysis of the small gains in patient GT1. A. Small gains <5 Mbp in patient GT1 related to known variants in various cancer types based on the COSMIC database.

## Data Availability

Code availability: The code used for filtering of variants is available at zenodo (https://doi.org/10.5281/zenodo.16785110). Phylogenetic trees: All segment files used to create the phylogenetic trees can be found in the supplementary material, File S1. The freely available software DEVOLUTION was used to generate phylogenetic trees: https://github.com/NatalieKAndersson/DEVOLUTION. All overview files can be found in the supplementary material, File S2 and all phylogenetic trees can be seen in the supplementary material, Figures [Link], [Link]. Statistical analyses: All input data used to create the statistical analyses in supplementary material, Figure S3 can be found in the supplementary material, File S1C–E. SNP‐array OSCHP‐files: The files may be made available upon request. TAPS‐plots: All TAPS‐plots used to assess the allelic imbalance of each detected CNA can be obtained upon request. TDS: All SNVs detected across all samples can be seen in the supplementary material, File S1.
