# Supplementary material for: Tracing metastatic spread in pediatric solid tumors using copy number and targeted deep sequencing
Source: J Pathol. 2025 Sep 23;267(3):347–65. doi: 10.1002/path.6472 (PMC12531126; doi:10.1002/path.6472)
Supplement: Supplementary file 1 — Data S1. Supporting Information Cover page [file PATH-267-347-s008.docx]

**Tracing metastatic spread in pediatric solid tumors using copy number and targeted deep sequencing**

N Andersson *et al. J Pathol* <https://doi.org/10.1002/path.6472>

**Supplementary Figures S1–S20 (provided as separate Word file)**

**Supplementary Figures S21–S40 (provided as separate Word file)**

**Supplementary Figure S41 (provided as separate Word file)**

**Supplementary Figures S42–S46 (provided as separate Word file)**

**Supplementary Data Files S1–S7 (provided as separate Excel files)**
