# Supplementary material for: Tracing metastatic spread in pediatric solid tumors using copy number and targeted deep sequencing
Source: J Pathol. 2025 Sep 23;267(3):347–65. doi: 10.1002/path.6472 (PMC12531126; doi:10.1002/path.6472)
Supplement: Supplementary file 2 — Figure S1. Workflow for somatic mutational analysis Figure S2. Workflow for copy number analysis Figure S3. Dotplots of the number of copy number alterations and the index of genomic diversity across samples and patients Figure S4. Private and shared genetic alterations in each anatomical location Figure S5. Neuroblastoma patient 1: Single nucleotide polymorphism (SNP)‐array data Figure S6. Neuroblastoma patient 2: Single nucleotide polymorphism (SNP)‐array data Figure S7. Neuroblastoma patient 3: Single nucleotide polymorphism (SNP)‐array data Figure S8. Neuroblastoma patient 5: Single nucleotide polymorphism (SNP)‐array data Figure S9. Neuroblastoma patient 6: Single nucleotide polymorphism (SNP)‐array data Figure S10. Neuroblastoma patient 7: Single nucleotide polymorphism (SNP)‐array data Figure S11. Neuroblastoma patient 8: Single nucleotide polymorphism (SNP)‐array data Figure S12. Neuroblastoma patient 9: Single nucleotide polymorphism (SNP)‐array data Figure S13. Wilms tumor patient 1: Single nucleotide polymorphism (SNP)‐array data Figure S14. Wilms tumor patient 2: Single nucleotide polymorphism (SNP)‐array data Figure S15. Wilms tumor patient 3: Single nucleotide polymorphism (SNP)‐array data Figure S16. Wilms tumor patient 5: Single nucleotide polymorphism (SNP)‐array data Figure S17. Wilms tumor patient 6: Single nucleotide polymorphism (SNP)‐array data Figure S18. Wilms tumor patient 8: Single nucleotide polymorphism (SNP)‐array data Figure S19. Wilms tumor patient 9: Single nucleotide polymorphism (SNP)‐array data Figure S20. Gonadal tumor patient 1: Single nucleotide polymorphism (SNP)‐array data [file PATH-267-347-s012.docx]

**Tracing metastatic spread in pediatric solid tumors using copy number and targeted deep sequencing**

N Andersson *et al. J Pathol* <https://doi.org/10.1002/path.6472>

**Supplementary Figures S1–S20**

**
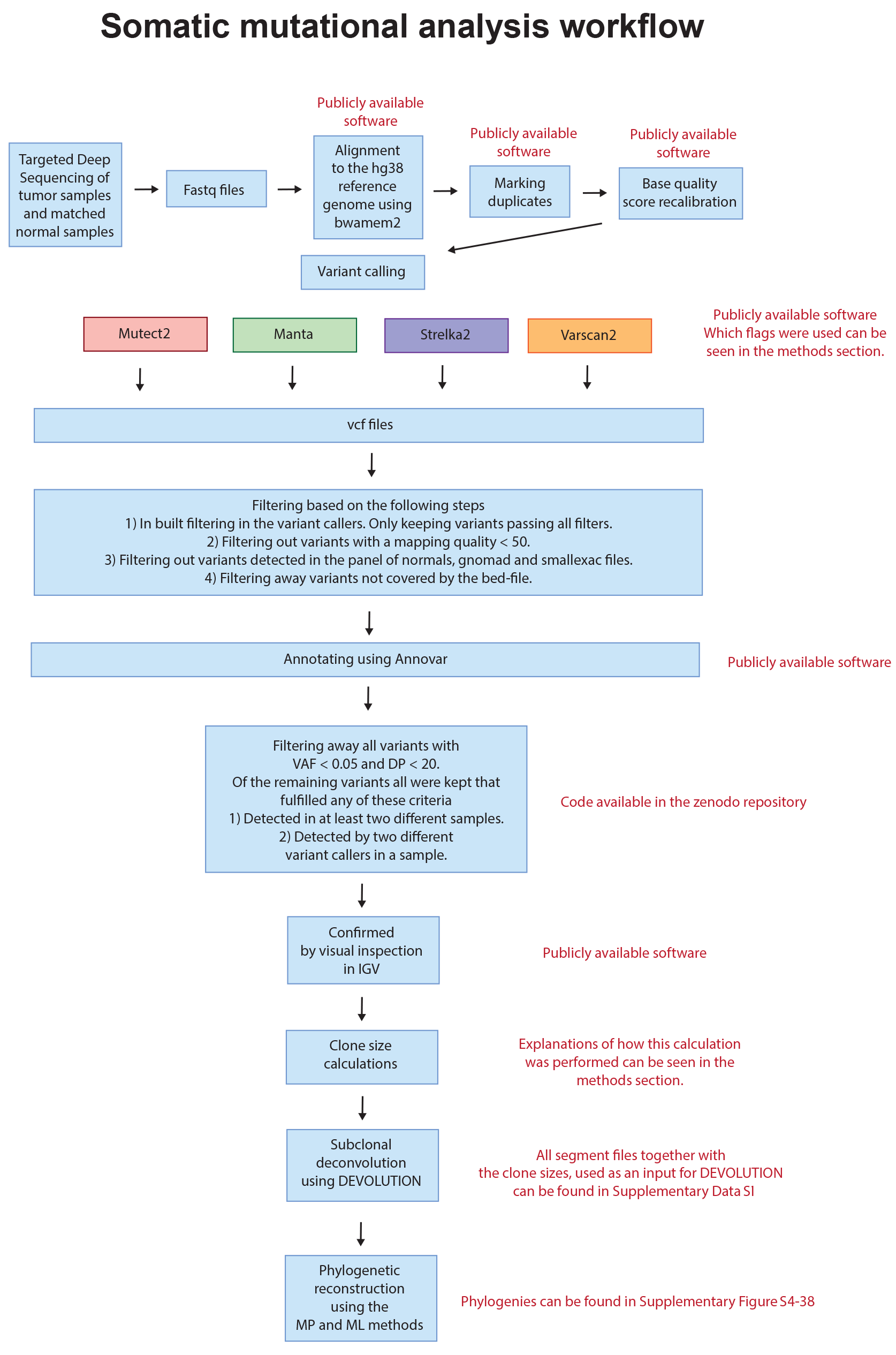
**

**Figure S1. Workflow for somatic mutational analysis.** Overview of the workflow performed for somatic mutational analysis. The red text indicates where the software or data can be found.

#####
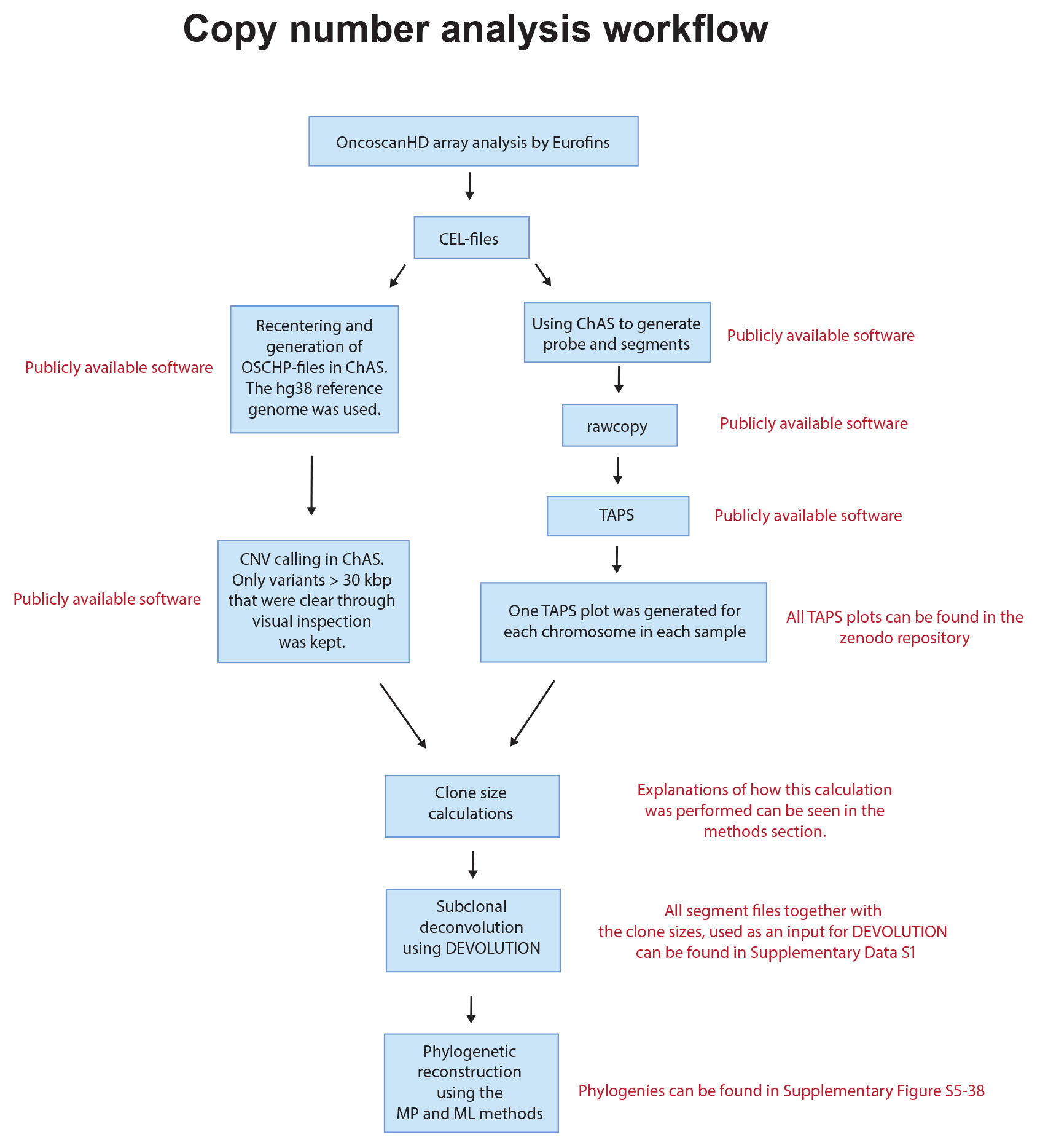


##### Figure S2. Workflow for copy number analysis. Overview of the workflow performed for copy number analysis. The red text indicates where the software or data can be found.

#####
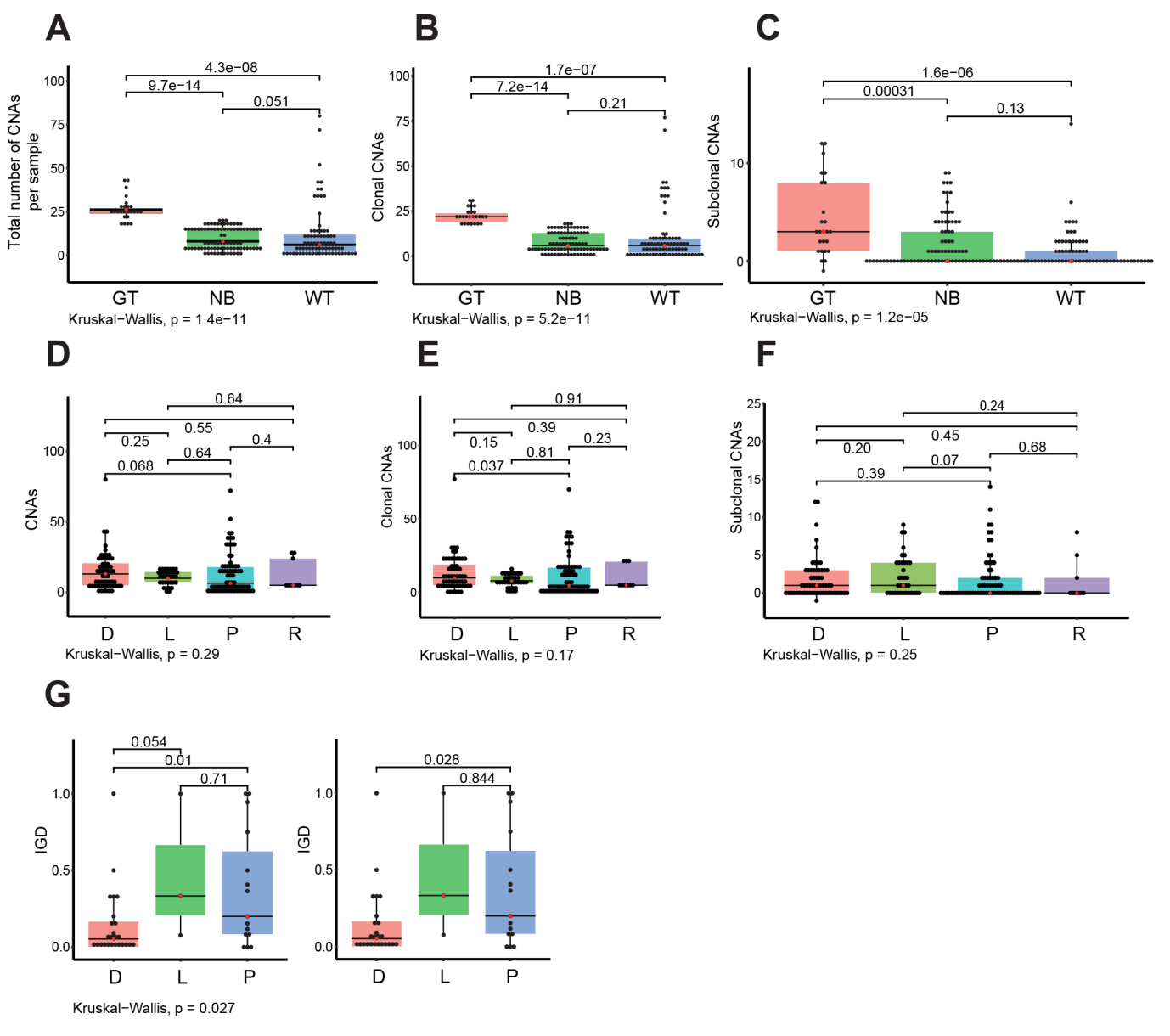


##### Figure S3. Dotplots of the number of copy number alterations and the index of genomic diversity across samples and patients. (A) Total number of CNAs in each sample, separated by tumor type. (B) Total number of clonal CNAs in each sample, separated by tumor type. (C) Total number of subclonal CNAs in each sample, separated by tumor type. (D) Total number of CNAs in each sample, separated by site where P is the primary tumor, L is locoregional lymph nodes, and D is distant sites. (E) Total number of clonal CNAs in each sample, separated by site. (F) Total number of subclonal CNAs in each sample, separated by site. (G) The index of genomic diversity (IGD) for each sample type in each patient, separated by site. Statistical analysis was performed using the Wilcoxon rank sum test (Mann–Whitney *U*-test). The Kruskal–Wallis test was used to assess whether the compared groups come from the same distribution or not. (H) A paired *t*-test between the IGD of the primary tumor (P) and distant metastases (D) and lymph node metastases (L). GT = Gonadal tumor, NB = Neuroblastoma, WT = Wilms tumor, D = Distant, L = Locoregional lymph node, P = Primary, R = Relapse.

#####
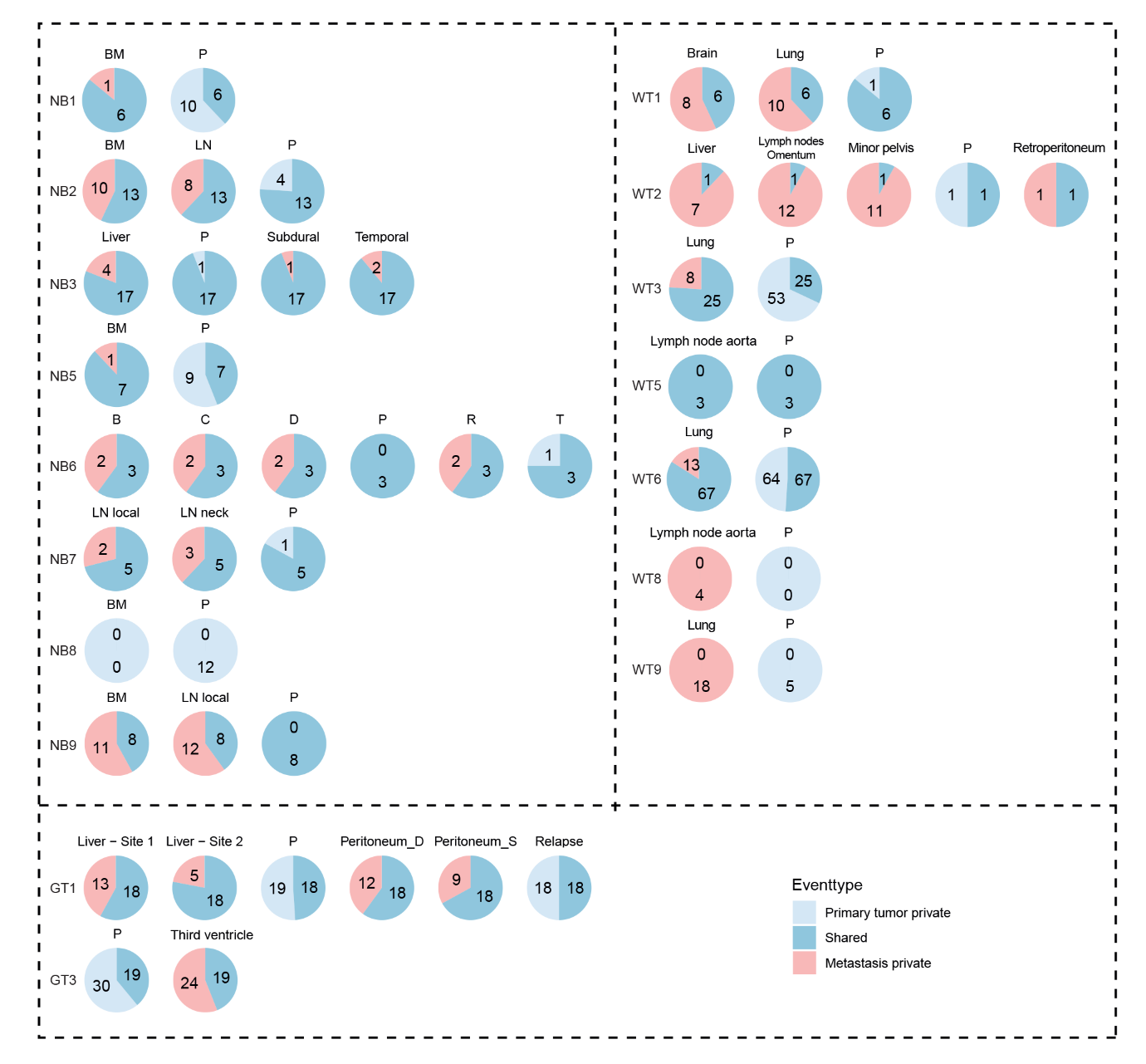


##### Figure S4. Private and shared genetic alterations in each anatomical location. Each row corresponds to a patient. For each patient there is one pie chart for the primary tumor and one pie chart for each metastasis. The pie chart for the primary tumor (P) illustrates the proportion of genetic alterations that are found in both the primary tumor and at least one metastasis (Shared) and the proportion of genetic alterations that are only found in the primary tumor (Primary tumor private). There is one pie chart for each metastatic site. These illustrate the proportion of genetic alterations that are not found in the primary tumor (Metastasis private) and the proportion of genetic alterations that are shared with the primary tumor (Shared). The numbers in the pie charts are the absolute number of genetic alterations. NB = Neuroblastoma, WT = Wilms tumor, GT = Gonadal tumor. The samples are the primary tumor (P), bone marrow (BM), lung (L), lymph nodes (N), subdural metastasis (S), temporal metastasis (T), the cortex (C), the dura mater (D), the brain (B), relapse (R), pituitary gland (Pi), liver (H), peritoneum close to the diaphragm (peri_D), peritoneum close to the spleen (peri_S), the retroperitoneum (Rp) and minor pelvis (Mp).

#####
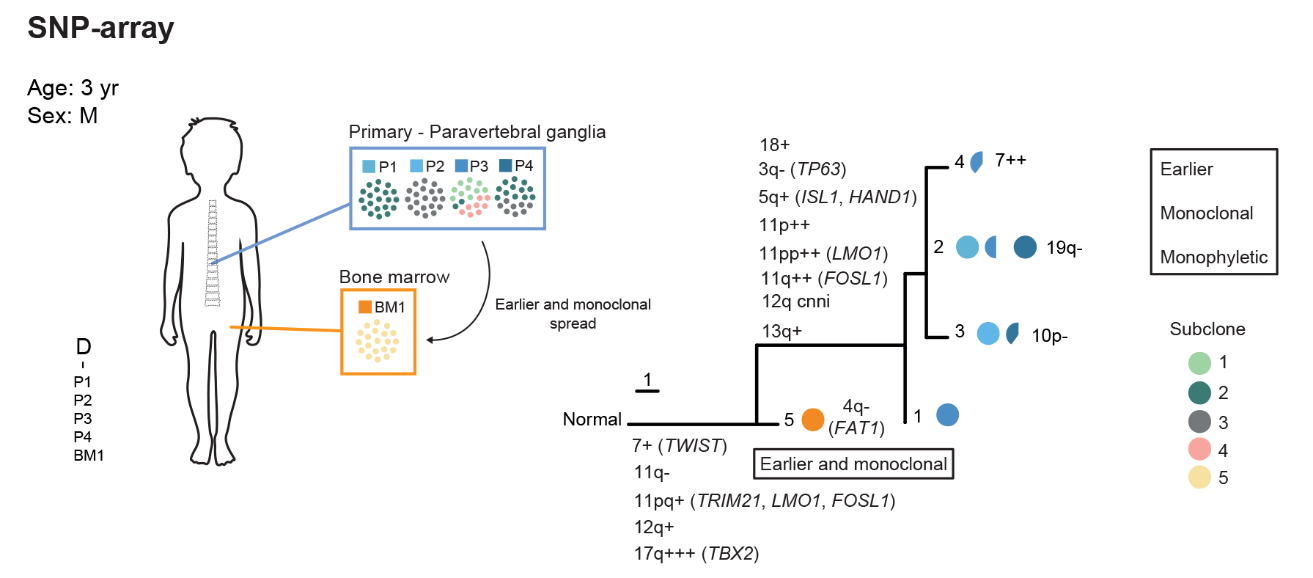


##### Figure S5. Neuroblastoma patient 1: Single-nucleotide polymorphism (SNP)-array data. The patient presented with a primary tumor in the paravertebral ganglia next to the spine (P1–P4) and a bone marrow metastasis (BM1) at diagnosis. In the phylogenetic tree, earlier and monoclonal spread to the bone marrow can be seen.

**
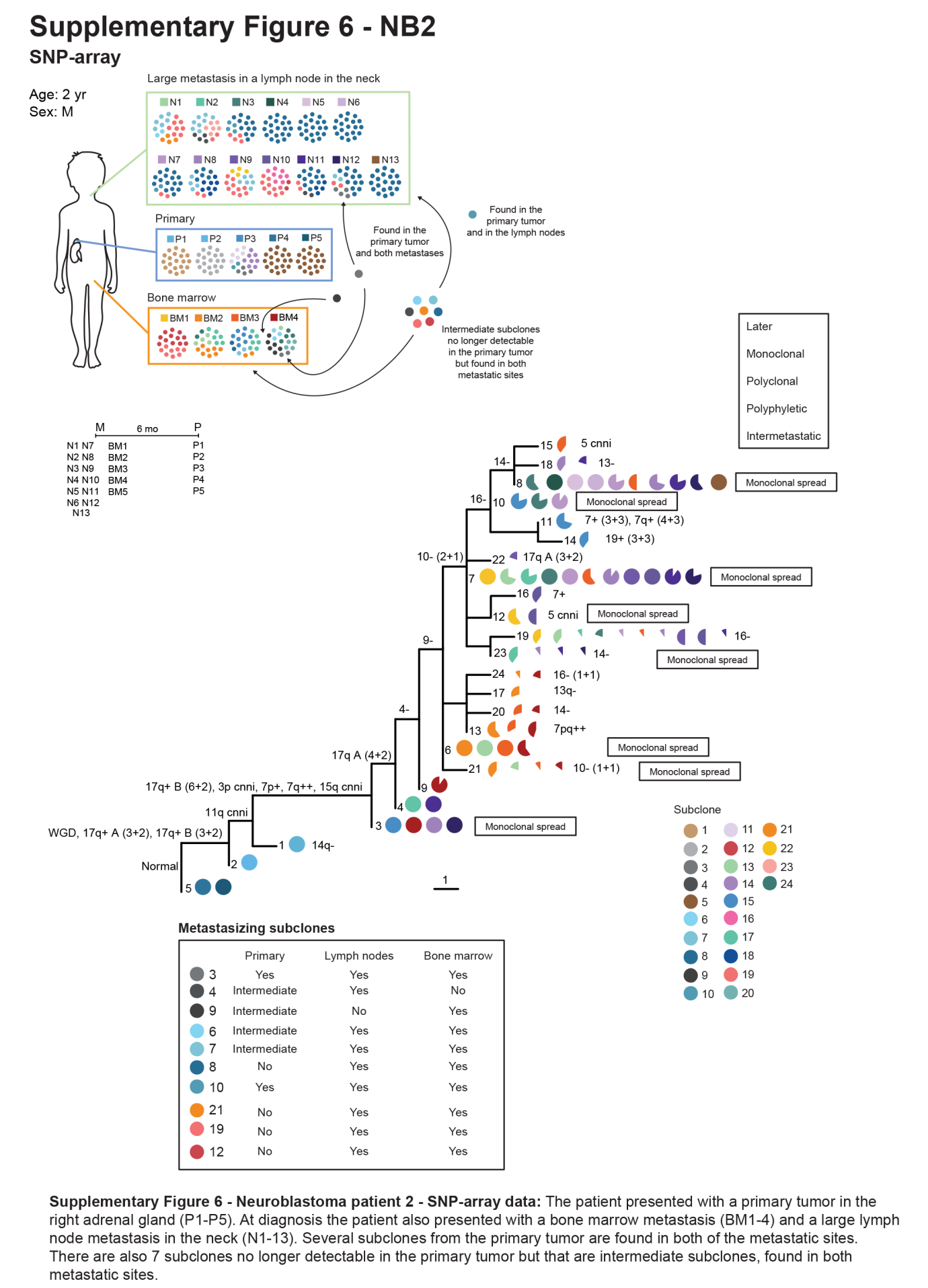
**

**Figure S6. Neuroblastoma patient 2: SNP-array data.** The patient presented with a primary tumor in the right adrenal gland (P1–P5). At diagnosis, the patient also presented with a bone marrow metastasis (BM1–4) and a large lymph node metastasis in the neck (N1–13). Several subclones from the primary tumor are found in both of the metastatic sites.

**
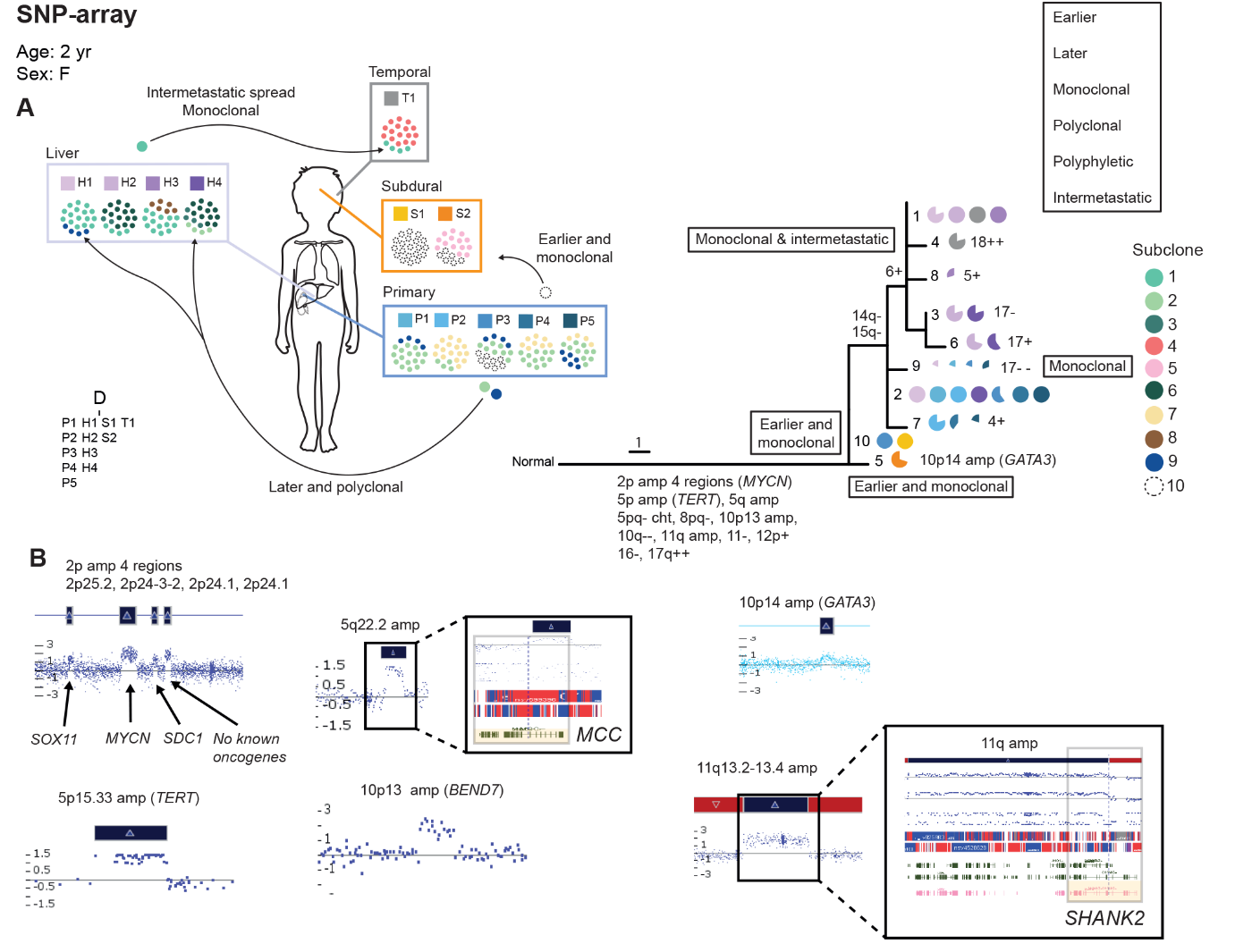
**

**Figure S7. Neuroblastoma patient 3: Single-nucleotide polymorphism (SNP)-array data.** The patient presented with a primary tumor in the right adrenal gland (P1–P5). At diagnosis, the patient also had a liver metastasis (H1–H4), a metastasis temporally (extracranially), and a metastasis subdurally intracranially (S1, S2). All samples have both an amplification of *MYCN* and *TERT*. In the phylogeny there is an earlier and monoclonal metastatic spread to the subdural space, with the metastasis having a small 10p amplification covering *GATA3*. There is also a later and polyclonal spread to the liver (subclone 2 and 9). Interestingly, subclone 1 is found both in the liver and, temporally, suggesting monoclonal intermetastatic spread. (B) Amplifications identified across samples. The log2 ratio is visualized on the *y*-axis and the genomic position on the *x*-axis.


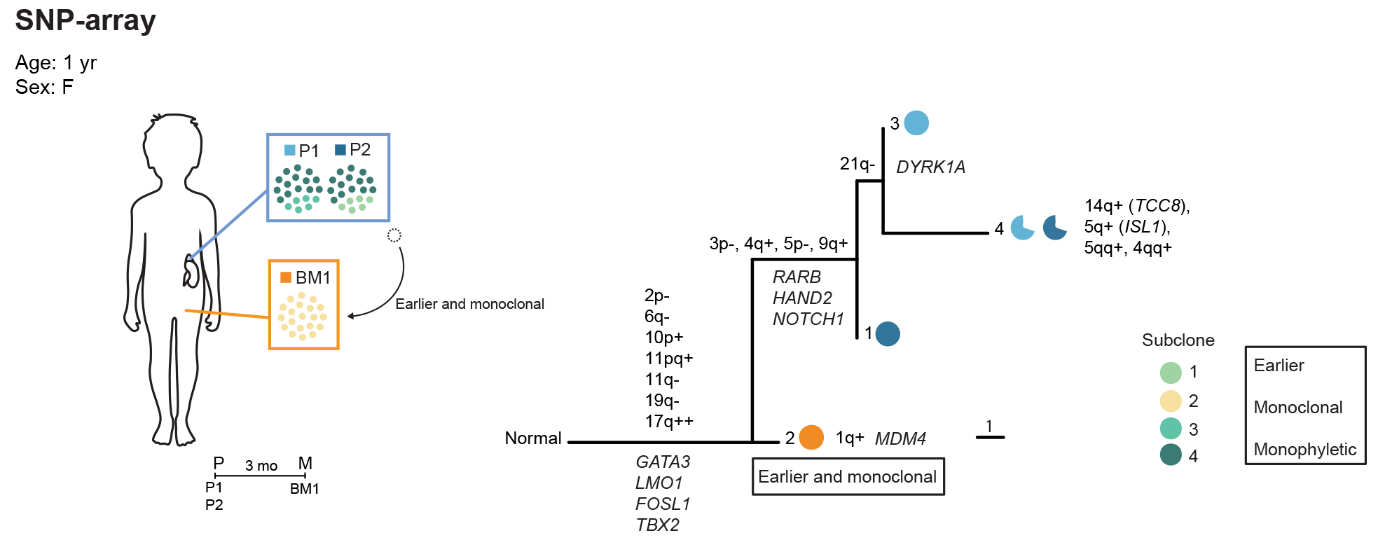


**Figure S8.** **Neuroblastoma patient 5: Single-nucleotide polymorphism (SNP)-array data.** The patient presented with a primary tumor in the left adrenal gland (P1, P2). After 3 months the patient also displayed a bone marrow metastasis (BM1). This arose early in the evolutionary history of the tumor and was monoclonal.


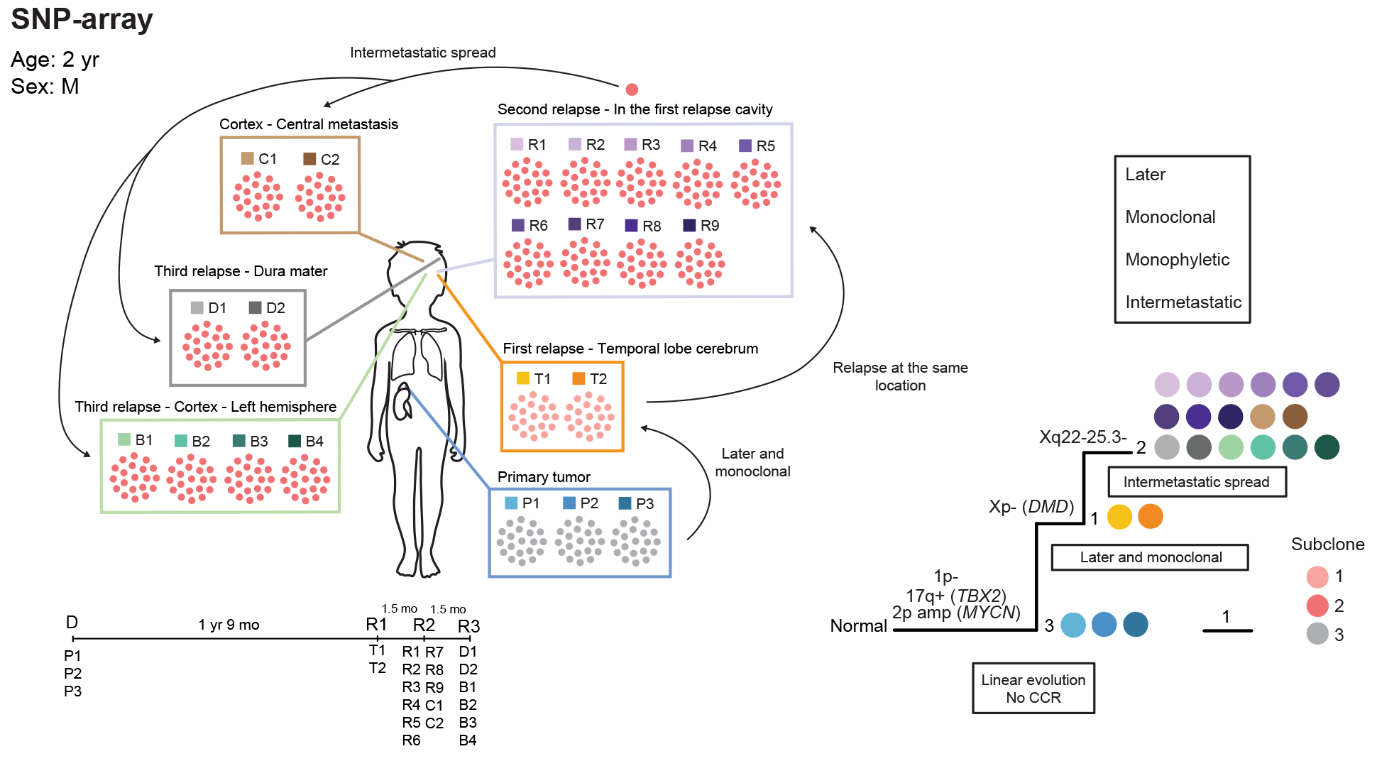


**Figure S9. Neuroblastoma patient 6: Single-nucleotide polymorphism (SNP)-array data.** The patient presented with a primary tumor in the right adrenal gland (P1–P3). The patient had a relapse after 1 year and 9 months, presenting in the temporal lobe in the brain (T1, T2), and a second relapse in the same cavity after surgery (R1–R9) as well as a central metastasis in the cortex of the brain (C1, C2) after 1.5 months. After a further1.5 months, the patient presented with a third relapse in the dura mater (D1, D2) and in the cortex in the left hemisphere of the brain (B1–B4). All samples had a 1p−, 17q+, and an *MYCN* amplification, all correlated with worse prognosis. Besides this, the metastases were homogenous.

**
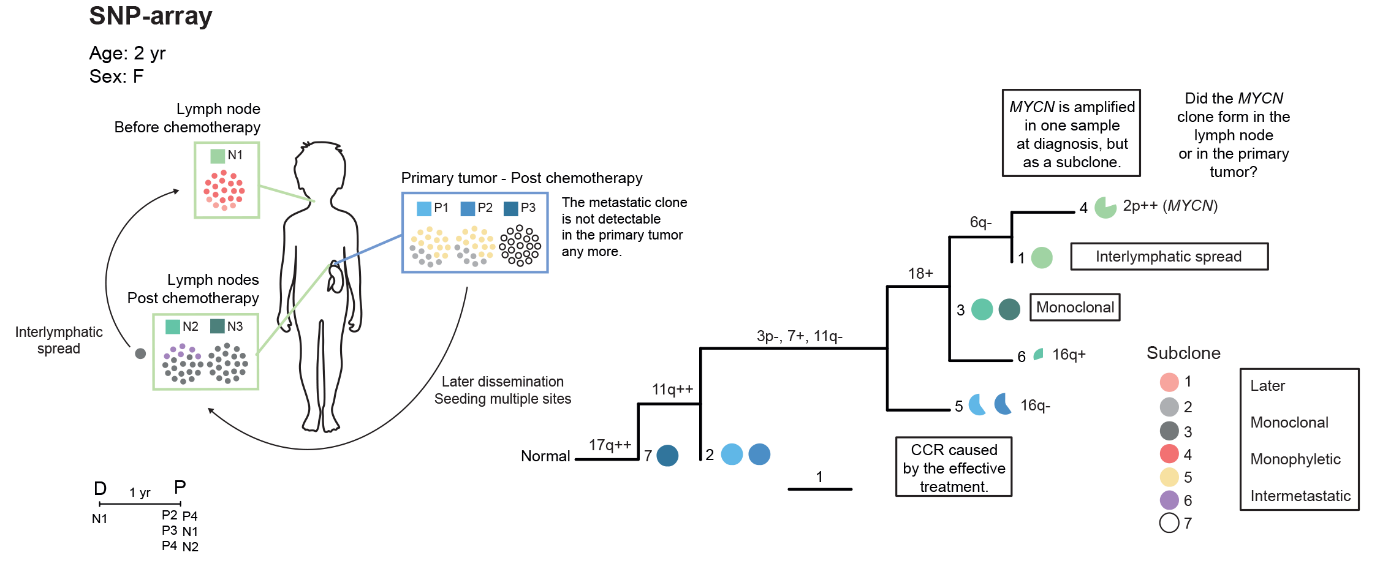
**

**Figure S10. Neuroblastoma patient 7: Single-nucleotide polymorphism (SNP)-array data.** The patient presented with a lymph node metastasis in the neck (N1). A primary tumor was identified in the left adrenal gland. The patient was treated with chemotherapy. After treatment, the primary tumor was surgically removed (P1–P3). Lymph node metastases locoregionally was also identified (N2, N3). Interestingly, the lymph node metastasis in the neck, identified at diagnosis had an *MYCN* amplification, and was not identified in the primary tumor. In the phylogeny it is clear that the metastases arose late in the evolutionary history of the tumor and there is a monoclonal spread from the locoregional lymph nodes to the lymph node in the neck. Most likely the *MYCN* amplification here arose locally.

**
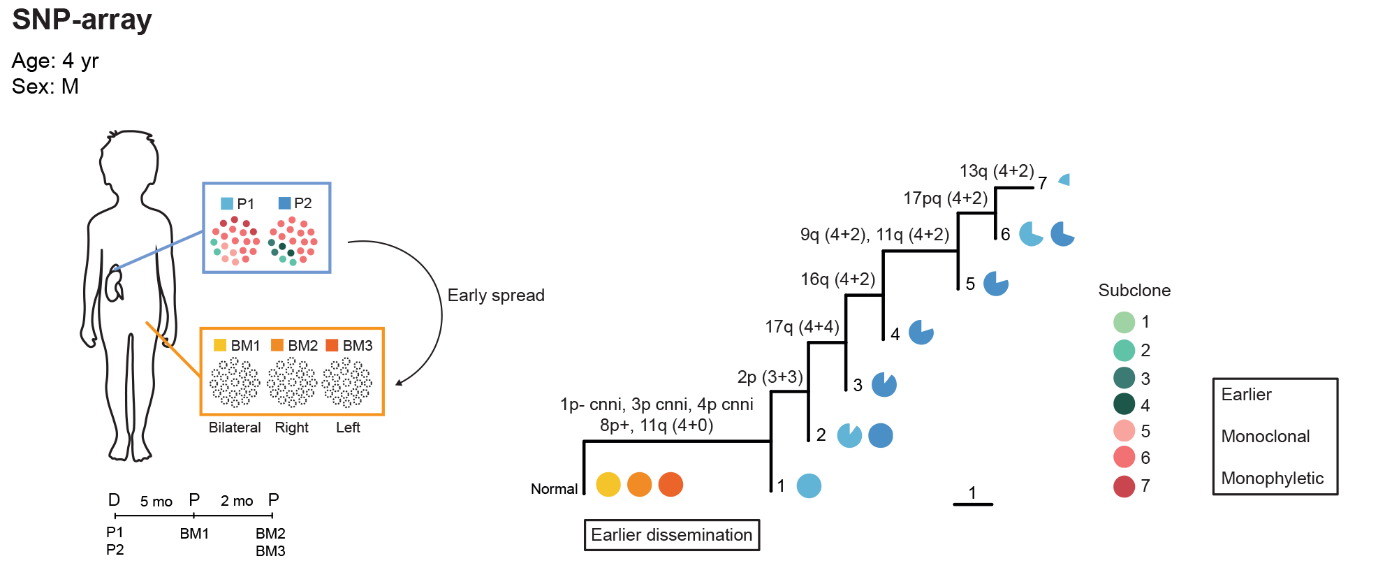
**

**Figure S11. Neuroblastoma patient 8: Single-nucleotide polymorphism (SNP)-array data.** The patient presented with a primary tumor in the right adrenal gland (P1, P2). After 5 months the patient also displayed a bone marrow metastasis (BM1) and after 2 months, it was still detectable (BM2, BM3). The bone marrow metastasis arose early in the evolutionary history of the tumor and was monoclonal.

**
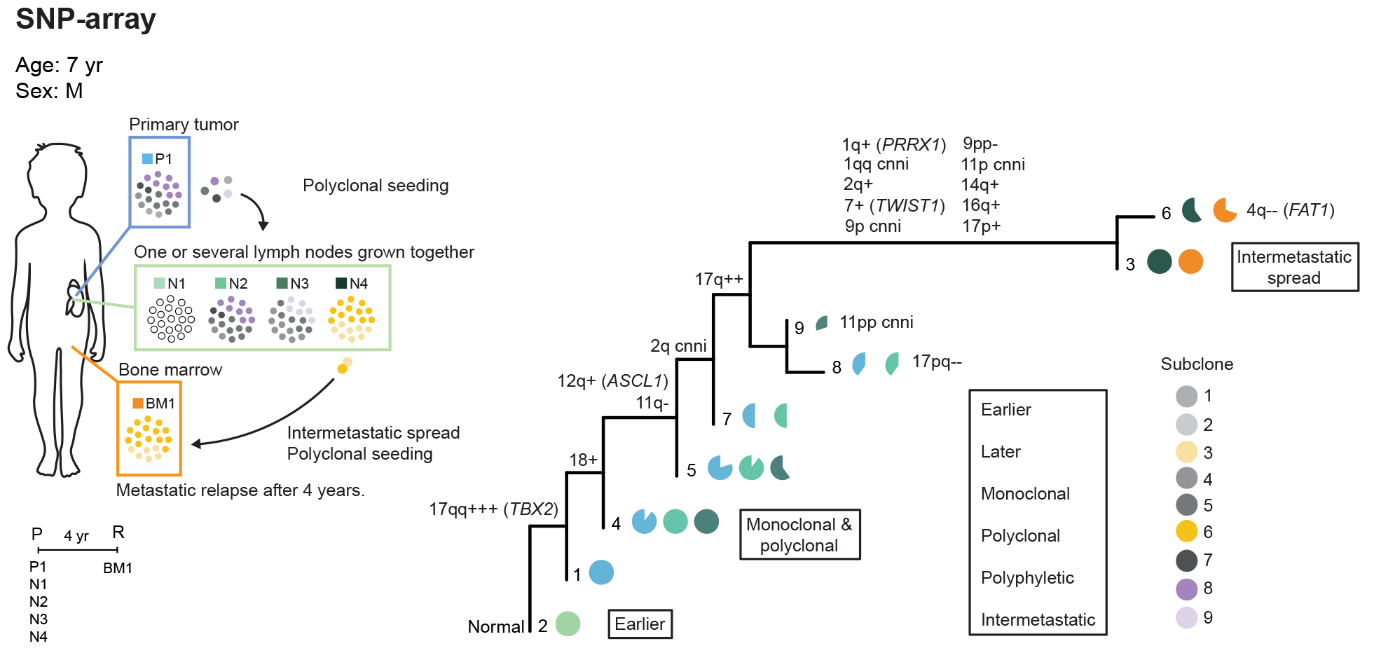
**

**Figure S12. Neuroblastoma patient 9: Single-nucleotide polymorphism (SNP)-array data.** The patient presented with a primary tumor in the left adrenal gland (P1). At diagnosis, there was also a lymph node conglomerate containing cancer cells (N1–N4). Interestingly several different subclones metastasized to the lymph nodes (subclone 2, 4, 5, 7, and 8). There are signs of early, late, monoclonal, and polyphyletic spread. One of the lymph node samples (N4) contained two subclones (subclones 3 and 6) having several additional genetic alterations, not found in the other sampled areas in the primary tumor and lymph node metastases. Strikingly, after 4 years the patient got a metastatic relapse in the bone marrow (BM1). presenting with the exact same two subclones (subclones 3 and 6) found in sample N4, suggesting that these subclones had entered a dormant state.

**
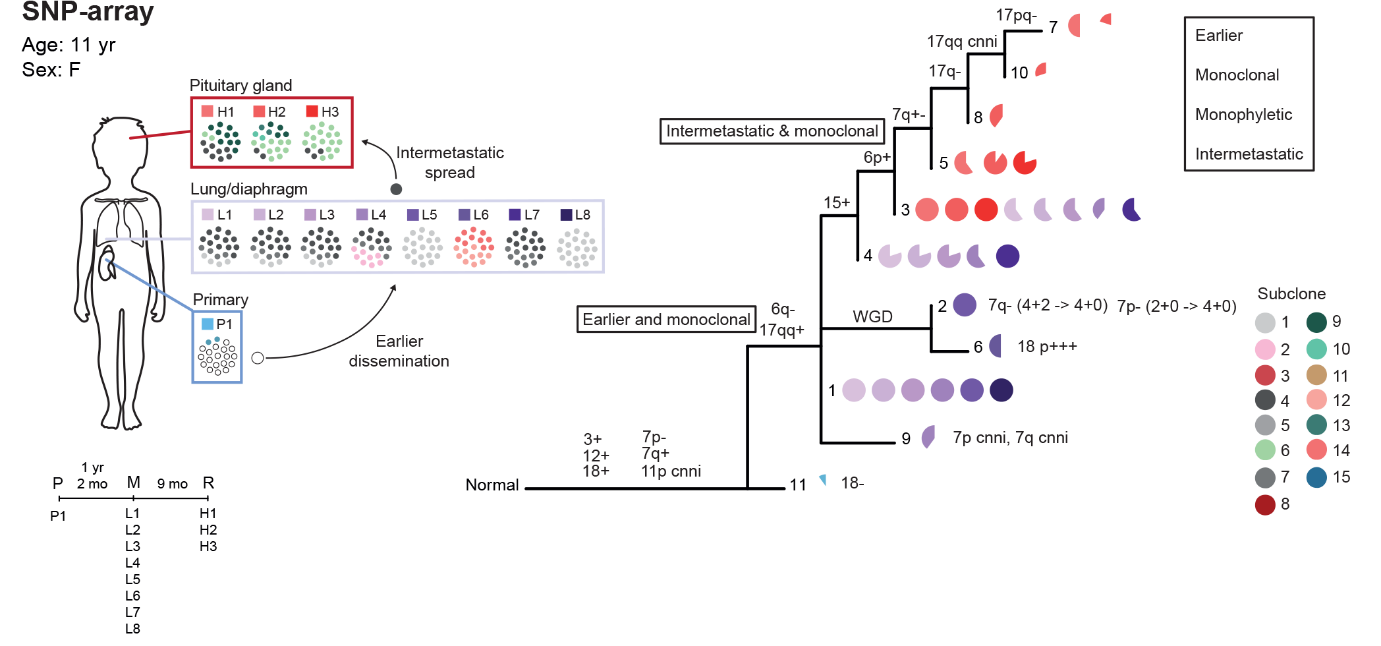
**

**Figure S13. Wilms tumor patient 1: Single-nucleotide polymorphism (SNP)-array data.** The patient presented with a primary tumor in the right kidney (P1). It only contained two different subclones, one having the stem aberrations and one having a loss of chromosome 18. After 1 year and 2 months the patient presented with a metastasis located in the lung, close to the diaphragm (L1–L8), having six different subclones. One of these were later identified in a brain metastasis to the pituitary gland (H1–H3), detected 9 months later, indicating intermetastatic spread from the lung to the brain.

**
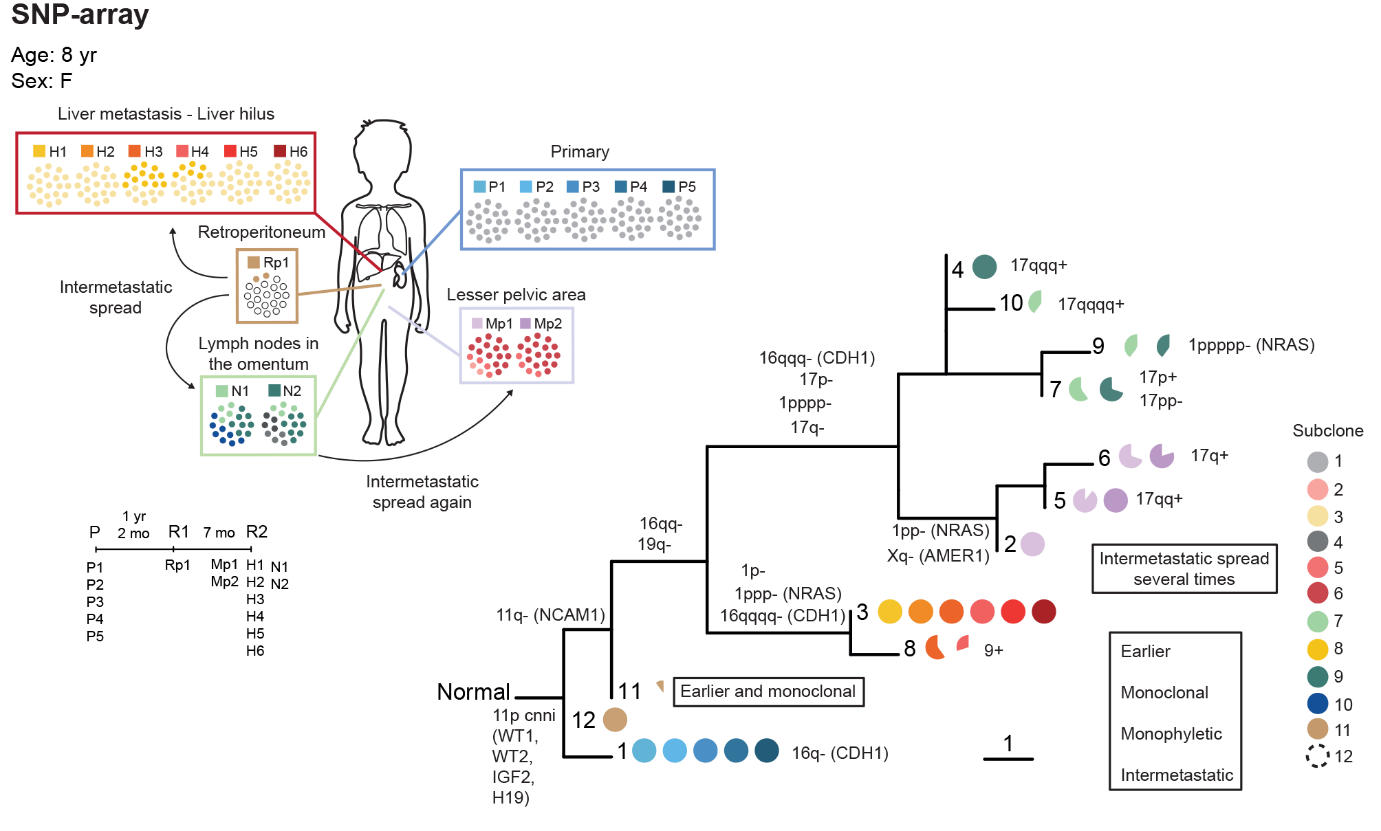
**

**Figure S14. Wilms tumor patient 2: Single-nucleotide polymorphism (SNP)-array data.** The patient presented with a primary tumor in the left kidney (P1–5). It was completely homogenous, only containing one single subclone. After 1 year and 2 months a metastasis was identified in the retroperitoneum (Rp1), which had spread early and monoclonally, according to the phylogenetic tree. It diverged before the subclone detected in all the primary tumor samples had formed. Strikingly, both the liver metastasis (H1–H6), metastasis in the minor pelvis (Mp1, Mp2), and lymph nodes in the omentum (N1, N2) are descendent to one minor subclone in Rp1 (subclone 11). There is also intermetastatic spread between the lymph nodes in the omentum and the lesser pelvic area, suggesting that intermetastatic spread can occur stepwise.

**
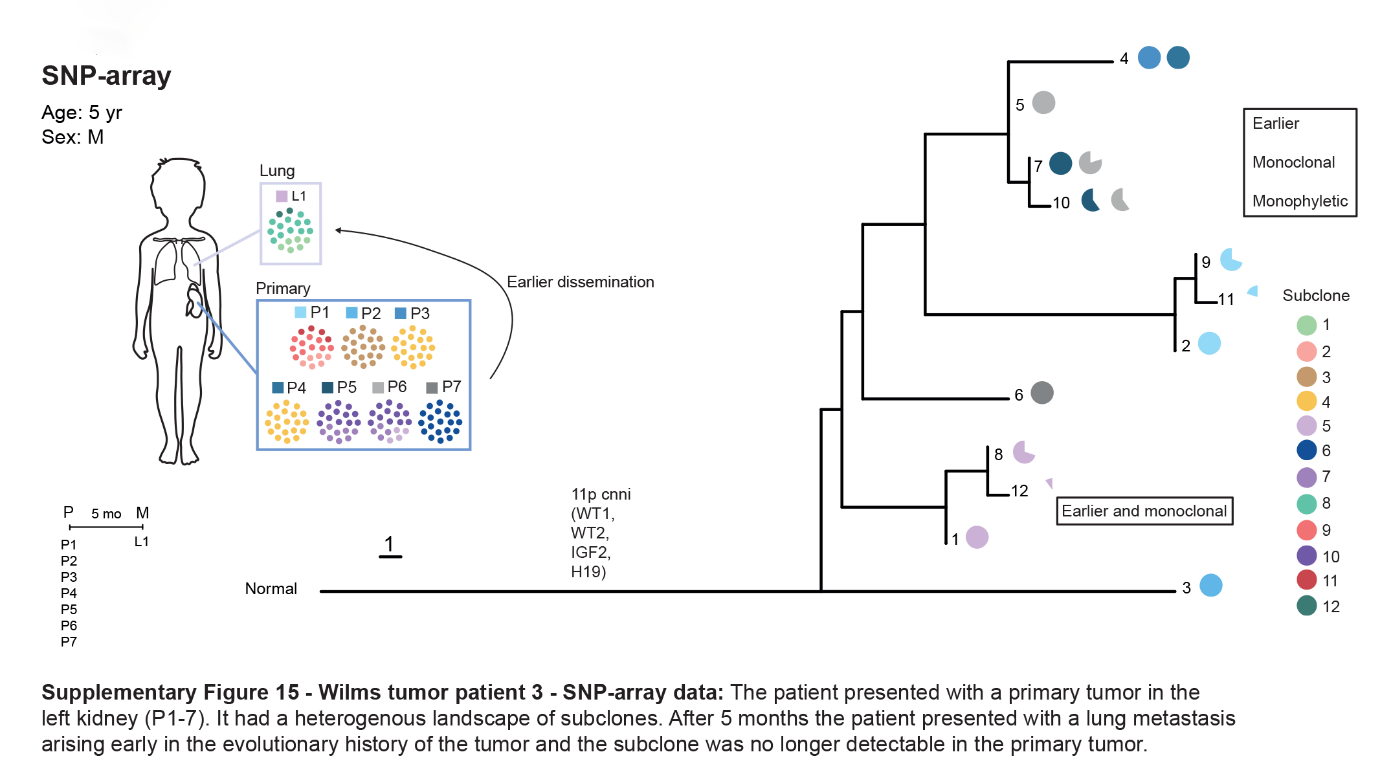
**

**Figure S15. Wilms tumor patient 3: Single-nucleotide polymorphism (SNP)-array data.** The patient presented with a primary tumor in the left kidney (P1–P7). It had a heterogenous landscape of subclones. After 5 months the patient presented with a lung metastasis arising early in the evolutionary history of the tumor and the subclone was no longer detectable in the primary tumor.

**
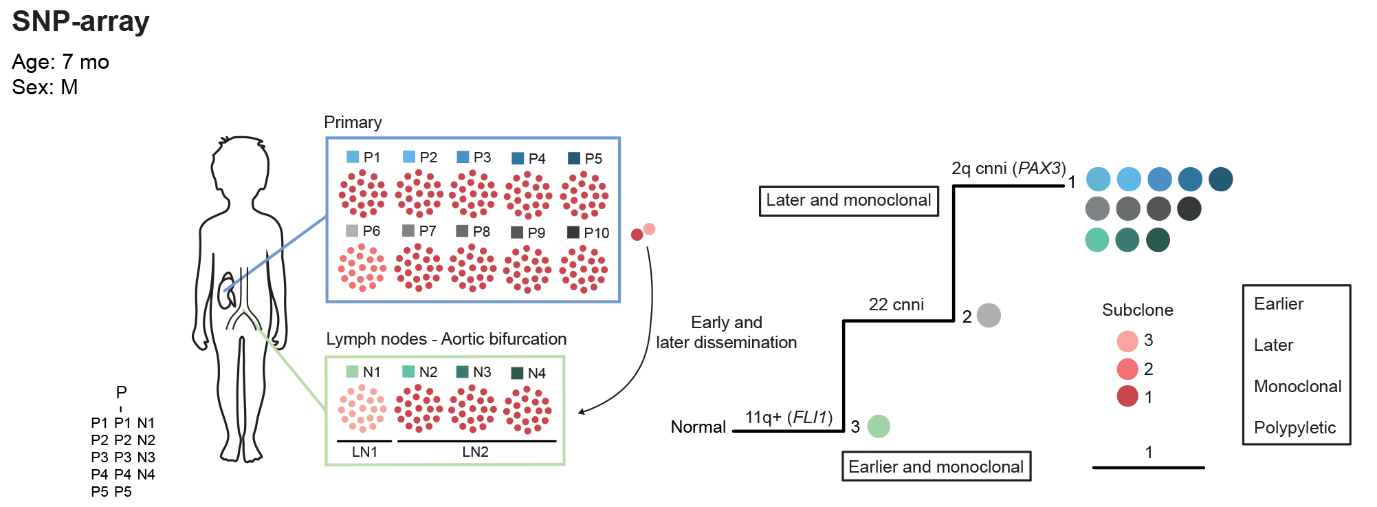
**

**Figure S16. Wilms tumor patient 5: Single-nucleotide polymorphism (SNP)-array data.** The patient presented with a primary tumor in the right kidney (P1–P10). The primary tumor merely contains two different subclones, and very few genetic aberrations. At diagnosis, two lymph node metastases (N1 and N2–N4) were identified in the aortic bifurcation. N1 diverged early and N2–N4 later in the evolutionary history of the tumor.

**
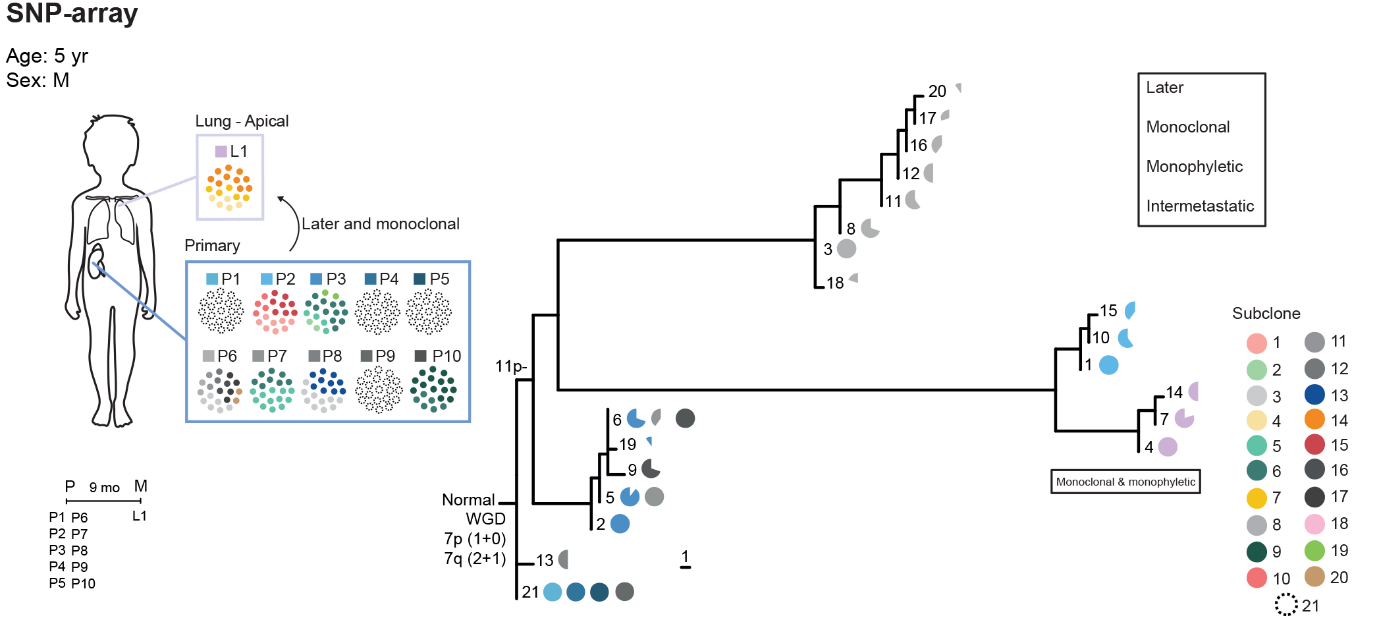
**

**Figure S17. Wilms tumor patient 6: Single-nucleotide polymorphism (SNP)-array data.** The patient presented with a primary tumor in the right kidney (P1–P10). It had a heterogenous landscape of subclones. After 9 months the patient presented with a lung metastasis (L1) arising late, but the subclone was not detectable in the primary tumor any more.

**
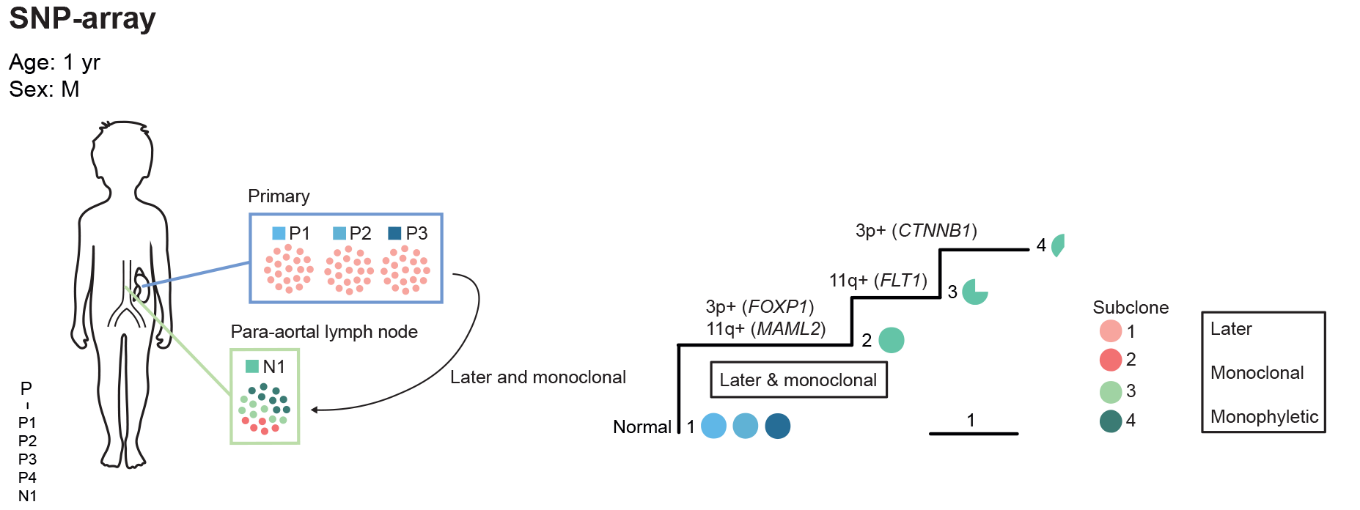
**

**Figure S18. Wilms tumor patient 8: Single-nucleotide polymorphism (SNP)-array data.** The patient presented with a primary tumor in the left kidney (P1–P3). It had a subclonal landscape with only a single subclone having no CNAs. The patient also had a lymph node metastasis para-aortally with three different subclones (N1), suggesting a late and monoclonal spread.

**
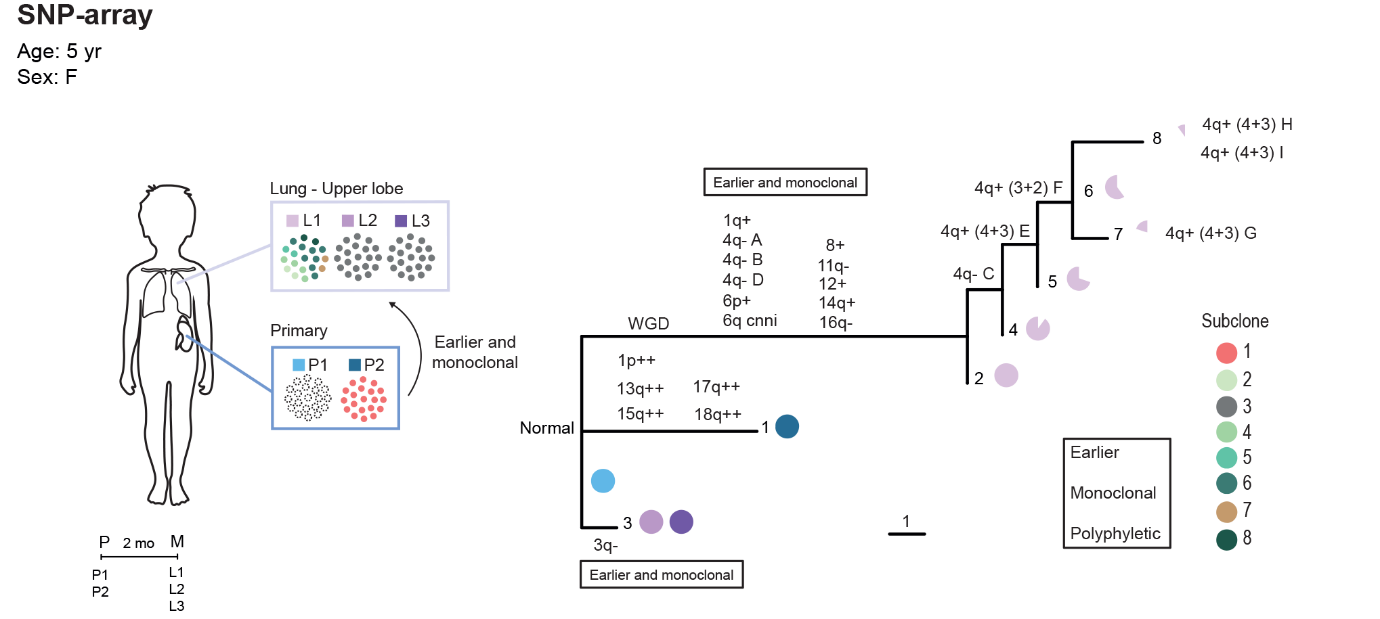
**

**Figure S19. Wilms tumor patient 9: Single-nucleotide polymorphism (SNP)-array data.** The patient presented with a primary tumor in the left kidney (P1, P2). It had a subclonal landscape with only two different subclones. After 2 months the patient presented with a lung metastasis. This had a diverse genomic landscape. Two samples were homogenous, containing subclone 3, while L3 had undergone a whole genome duplication (WGD) and had several additional genetic alterations.

**
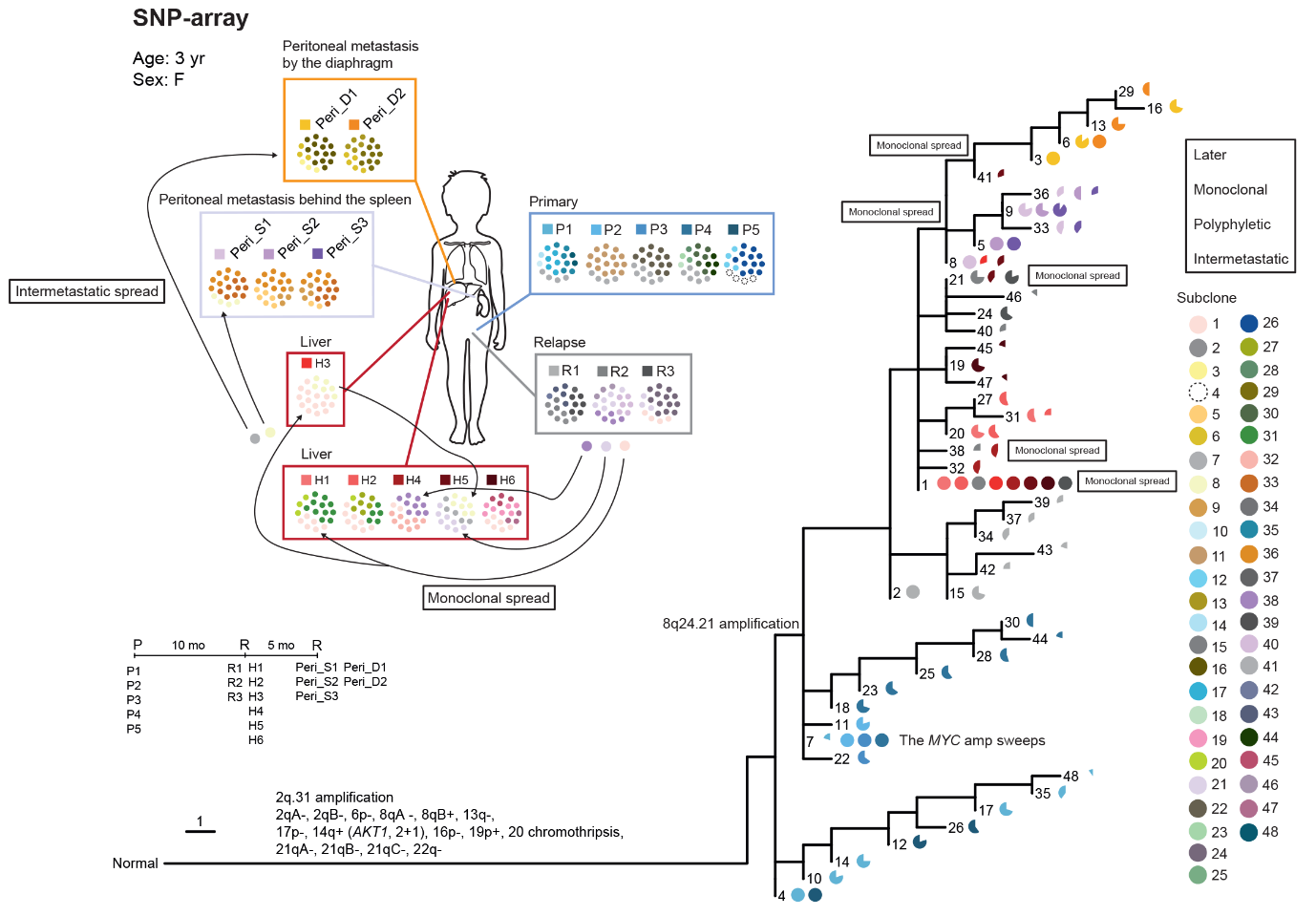
**

**S20. Gonadal tumor patient 1: Single-nucleotide polymorphism (SNP)-array data.** The patient had a juvenile granulosa cell tumor with the primary tumor located in the left ovary (P1–5). Area P1 harbors an *MYC* amplification, which sweeps in area P2–4, while area P5 lacks it. After 10 months the patient presented with a relapse in the left ovarium. All cells in the relapse (R1–3) and all metastases have the *MYC* amplification. Three different subclones in the relapse (subclones 1, 38, and 21) spread to two different liver metastases (H3 and H1, H2, H4–H6). Two subclones in the liver metastases spread to the peritoneum behind the spleen (Peri_S1–S3) and the peritoneum close to the diaphragm (Peri_D1, D2), via intermetastatic spread. There is also intermetastatic spread between the two liver metastases.
