# Supplementary material for: Tracing metastatic spread in pediatric solid tumors using copy number and targeted deep sequencing
Source: J Pathol. 2025 Sep 23;267(3):347–65. doi: 10.1002/path.6472 (PMC12531126; doi:10.1002/path.6472)
Supplement: Supplementary file 3 — Figure S21. Gonadal tumor patient 3: Single nucleotide polymorphism (SNP)‐array data Figure S22. Neuroblastoma patient 1: targeted deep sequencing (TDS) data Figure S23. Neuroblastoma patient 1: targeted deep sequencing (TDS) and single nucleotide polymorphism (SNP)‐array data Figure S24. Neuroblastoma patient 2: targeted deep sequencing (TDS) data Figure S25. Neuroblastoma patient 2: targeted deep sequencing (TDS) data and SNP array data—maximum parsimony (MP) tree Figure S26. Neuroblastoma patient 2: targeted deep sequencing (TDS) data and SNP array data—maximum likelihood (ML) tree Figure S27. Neuroblastoma patient 8: targeted deep sequencing (TDS) data—Maximum parsimony (MP) tree Figure S28. Neuroblastoma patient 8: targeted deep sequencing (TDS) data—Maximum likelihood tree (ML) Figure S29. Wilms tumor patient 1: targeted deep sequencing (TDS) data Figure S30. Wilms tumor patient 1: targeted deep sequencing (TDS) and single nucleotide polymorphism (SNP)‐array data Figure S31. Wilms tumor patient 2: targeted deep sequencing (TDS) Figure S32. Wilms tumor patient 2: targeted deep sequencing (TDS) and single nucleotide polymorphism (SNP)‐array data Figure S33. Wilms tumor patient 8: targeted deep sequencing (TDS) data Figure S34. Wilms tumor patient 8: targeted deep sequencing (TDS) and single nucleotide polymorphism (SNP)‐array data Figure S35. Wilms tumor patient 9: targeted deep sequencing (TDS) data Figure S36. Wilms tumor patient 9: targeted deep sequencing (TDS) and single nucleotide polymorphism (SNP)‐array data Figure S37. Gonadal tumor patient 1: targeted deep sequencing (TDS) data Figure S38. Gonadal tumor patient 1: targeted deep sequencing (TDS) and single nucleotide polymorphism (SNP)‐array data Figure S39. Overview of the metastasis patterns across patients NB1–NB9 and WT1 Figure S40. Overview of the metastasis patterns across patients WT2–WT9 and GT1 and GT3 [file PATH-267-347-s001.docx]

**Tracing metastatic spread in pediatric solid tumors using copy number and targeted deep sequencing**

N Andersson *et al. J Pathol* <https://doi.org/10.1002/path.6472>

**Supplementary Figures S21–S40**

**
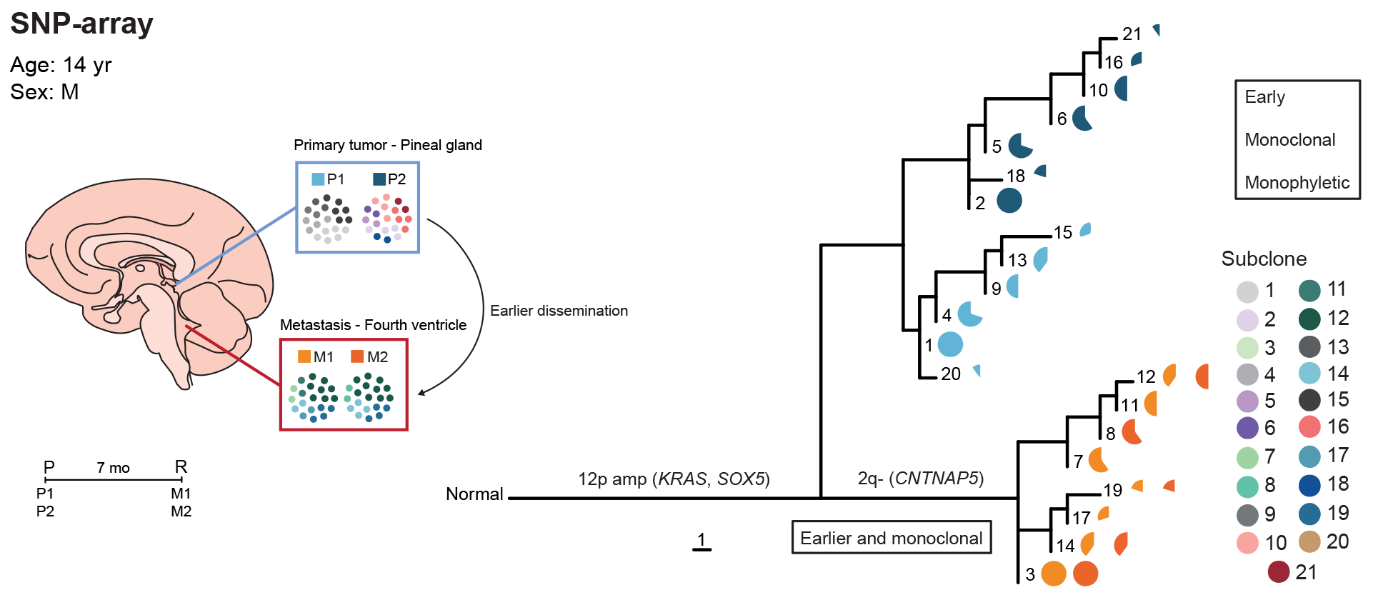
**

**Figure S21. Gonadal tumor patient 3: Single nucleotide polymorphism (SNP)-array data.** The patient presented with a primary teratoma mixed with an embryonal carcinoma located in the pineal gland in the brain (P1, P2). The samples were very genetically diverse. After 7 months the patient presented with a metastatic relapse in the fourth ventricle in the brain (M1, M2). The metastasis arose via an evolutionarily early and monoclonal spread. It might already have been present at diagnosis, but too small to be detectable.

**
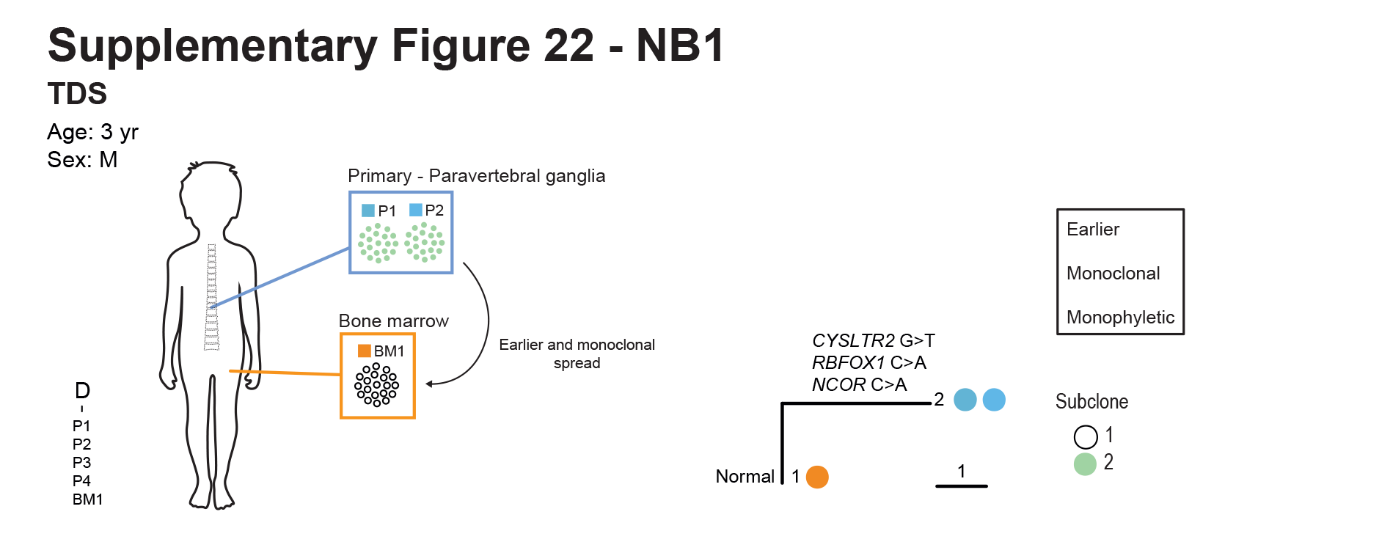
**

**Figure S22. Neuroblastoma patient 1: targeted deep sequencing (TDS) data.** The patient presented with a primary tumor in the paravertebral ganglia next to the spine (P1, P2) and a bone marrow metastasis (BM1) at diagnosis. In the phylogenetic tree an early and monoclonal spread to the bone marrow can be seen.

**
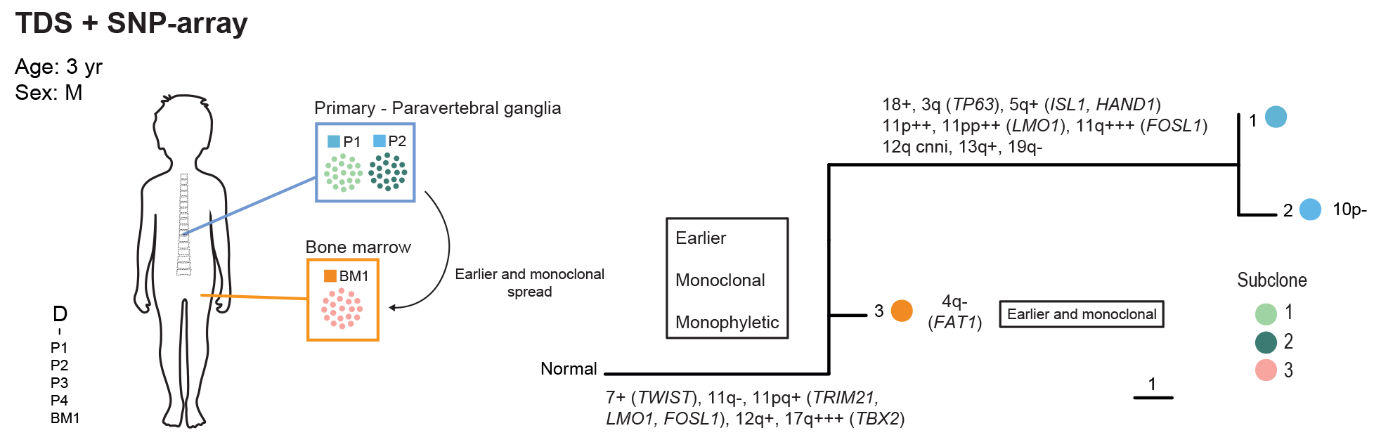
**

**Figure S23. Neuroblastoma patient 1: targeted deep sequencing (TDS) and single nucleotide polymorphism (SNP)-array data.** The patient presented with a primary tumor in the paravertebral ganglia next to the spine (P1, P2) and a bone marrow metastasis (BM1) at diagnosis. In the phylogenetic tree an early and monoclonal spread to the bone marrow can be seen.

**
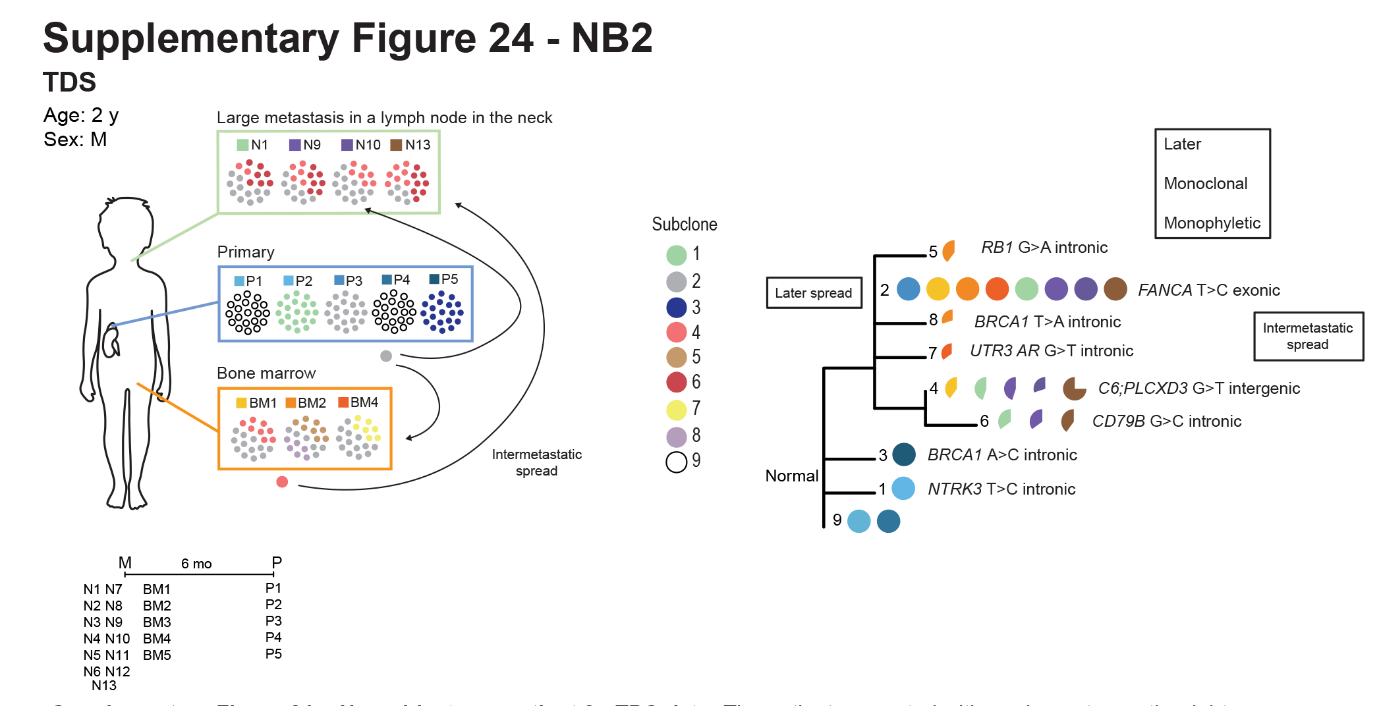
**

**Figure S24. Neuroblastoma patient 2: targeted deep sequencing (TDS) data.** The patient presented with a primary tumor the right adrenal gland (P1–P5). At diagnosis there was a large metastasis in the lymph nodes in the neck (N1, N9, N10 and N13) and a bone marrow metastasis (BM1, BM2 and BM4). The primary tumor was sampled after chemotherapy. There was late metastatic spread to both the lymph nodes and the bone marrow (subclone 2). Either the primary tumor disseminated to both separately, or there might have been an intermetastatic spread between the sites. Subclone 4 has spread from the bone marrow to the lymph nodes in the neck, suggesting intermetastatic spread.

**
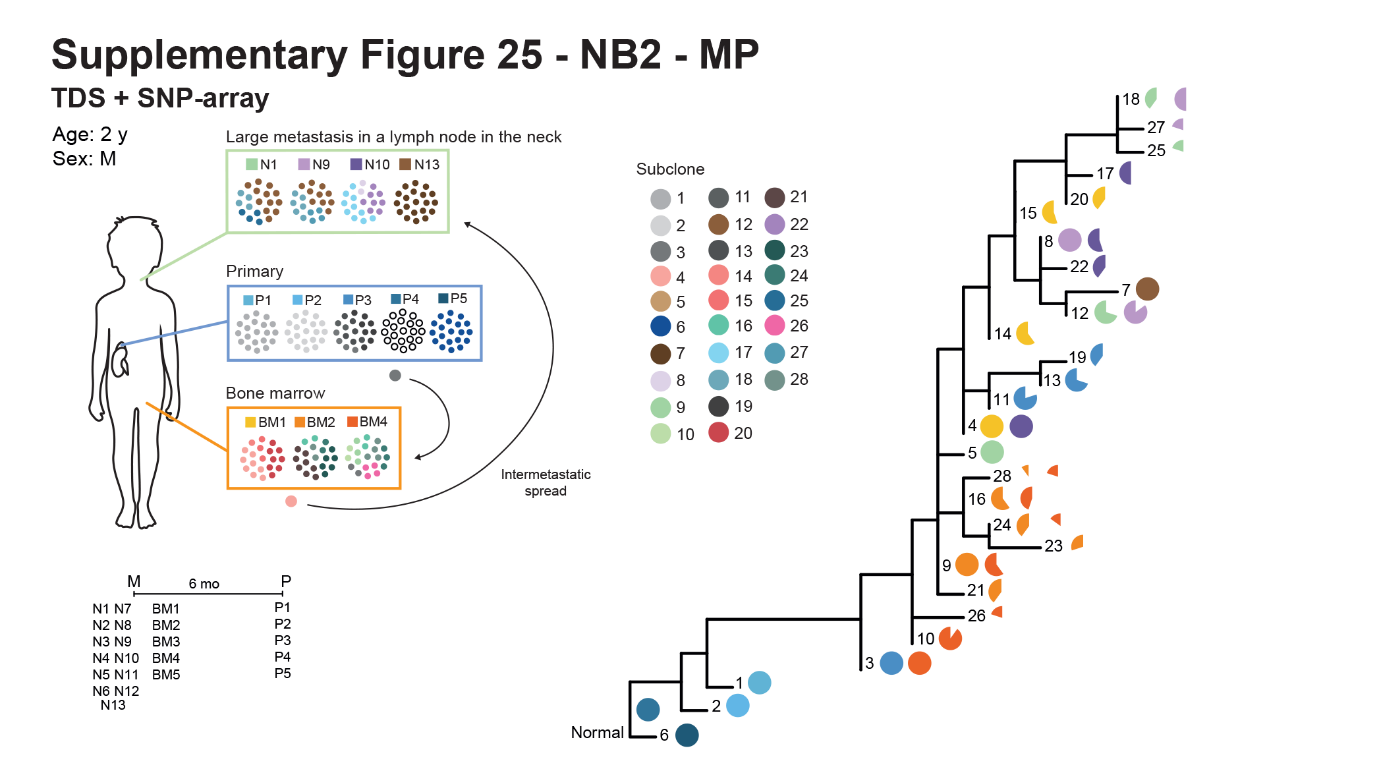
**

**Figure S25. Neuroblastoma patient 2: targeted deep sequencing (TDS) and SNP-array data: maximum parsimony (MP) tree.** The patient presented with a primary tumor the right adrenal gland (P1–P5). At diagnosis there was a large metastasis in the lymph nodes in the neck (N1, N9, N10 and N13) and a bone marrow metastasis (BM1, BM2 and BM4). The primary tumor was sampled after chemotherapy. Several subclones from the primary tumor are found in both of the metastatic sites. There are also several subclones no longer detectable in the primary tumor but that are intermediate subclones, found in both metastatic sites.

**
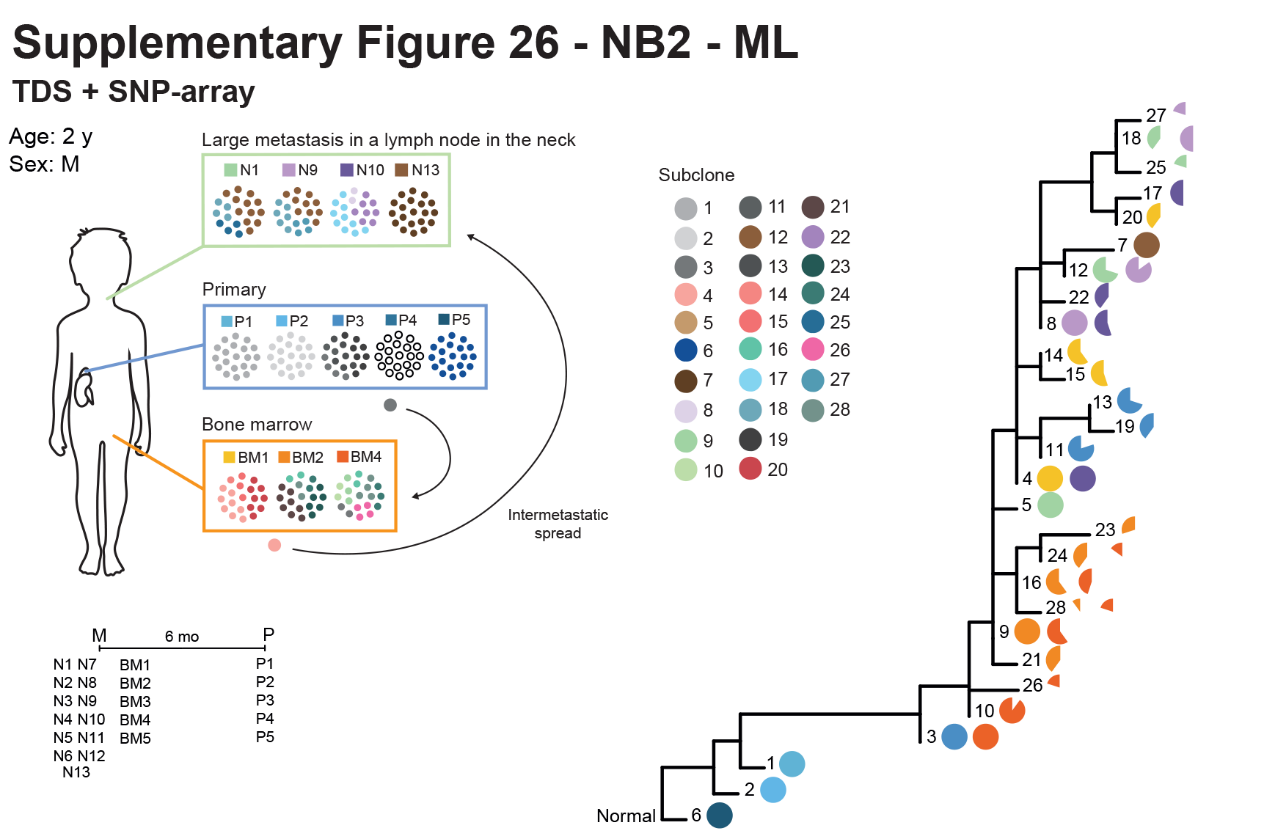
**

**Figure S26. Neuroblastoma patient 2: targeted deep sequencing (TDS) and SNP-array data - maximum likelihood (ML) tree.** The patient presented with a primary tumor on the right adrenal gland (P1–P5). At diagnosis there was a large metastasis in the lymph nodes in the neck (N1, N9, N10 and N13) and a bone marrow metastasis (BM1, BM2 and BM4). The primary tumor was sampled after chemotherapy. Several subclones from the primary tumor are found in both of the metastatic sites. There are also several subclones no longer detectable in the primary tumor but that are intermediate subclones, found in both metastatic sites.


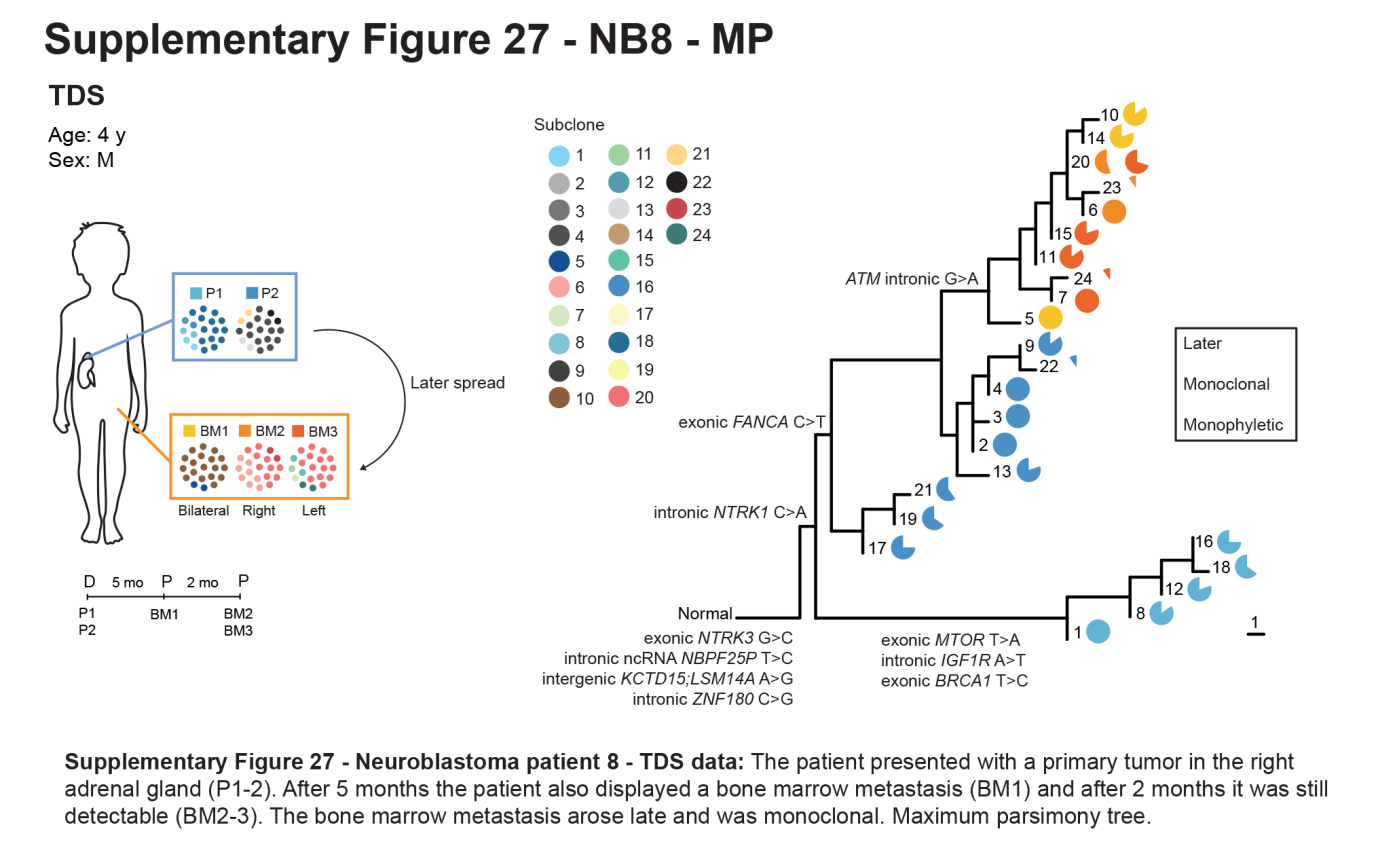


**Figure S27. Neuroblastoma patient 8: targeted deep sequencing (TDS) data – Maximum parsimony (MP) tree.** The patient presented with a primary tumor in the right adrenal gland (P1, P2). After 5 months the patient also displayed a bone marrow metastasis (BM1) and after 2 months it was still detectable (BM2, BM3). The bone marrow metastasis arose late and was monoclonal. Maximum parsimony tree.

**
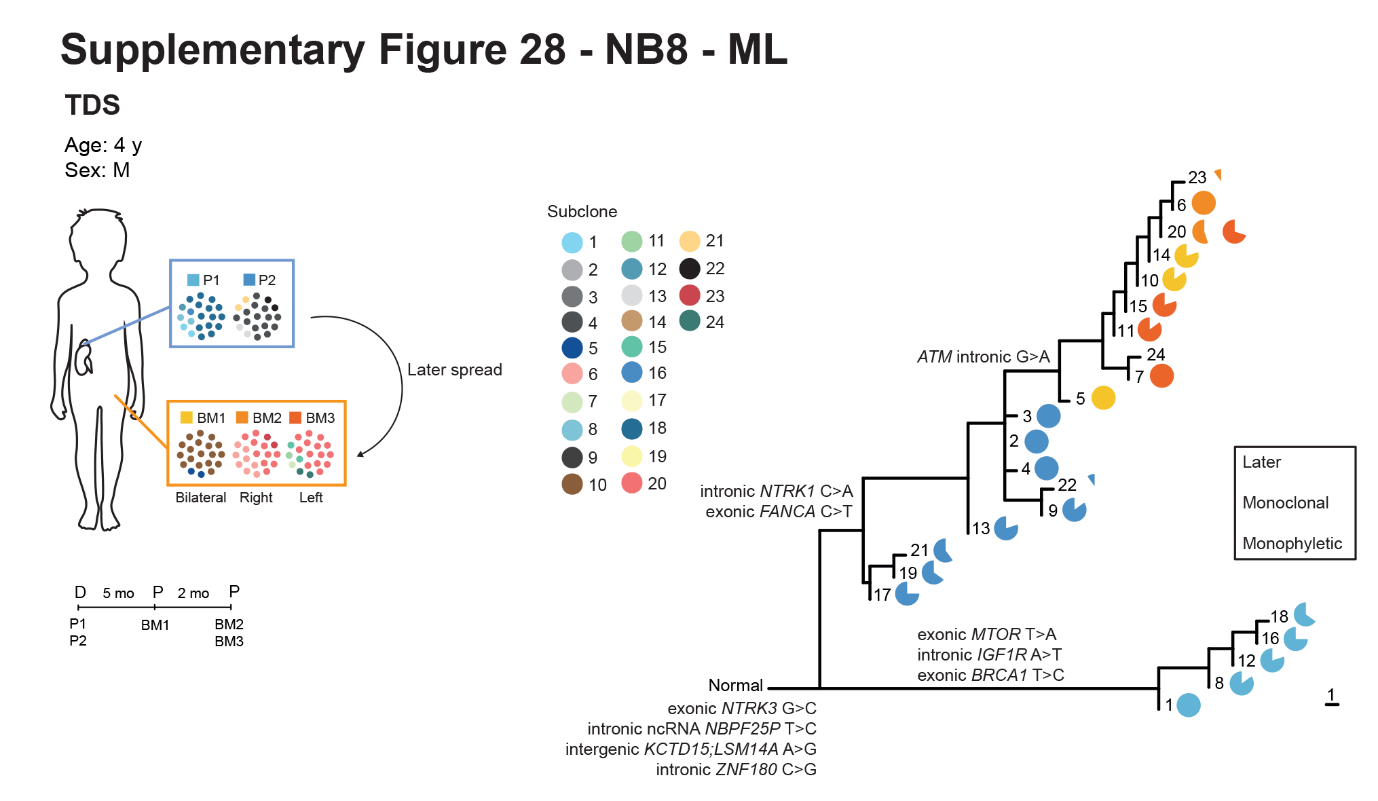
**

**Figure S28. Neuroblastoma patient 8: targeted deep sequencing (TDS) data – Maximum likelihood (ML) tree.** The patient presented with a primary tumor in the right adrenal gland (P1, P2). After 5 months the patient also displayed a bone marrow metastasis (BM1) and after 2 months it was still detectable (BM2, BM3). The bone marrow metastasis arose late and was monoclonal. Maximum likelihood tree.

**
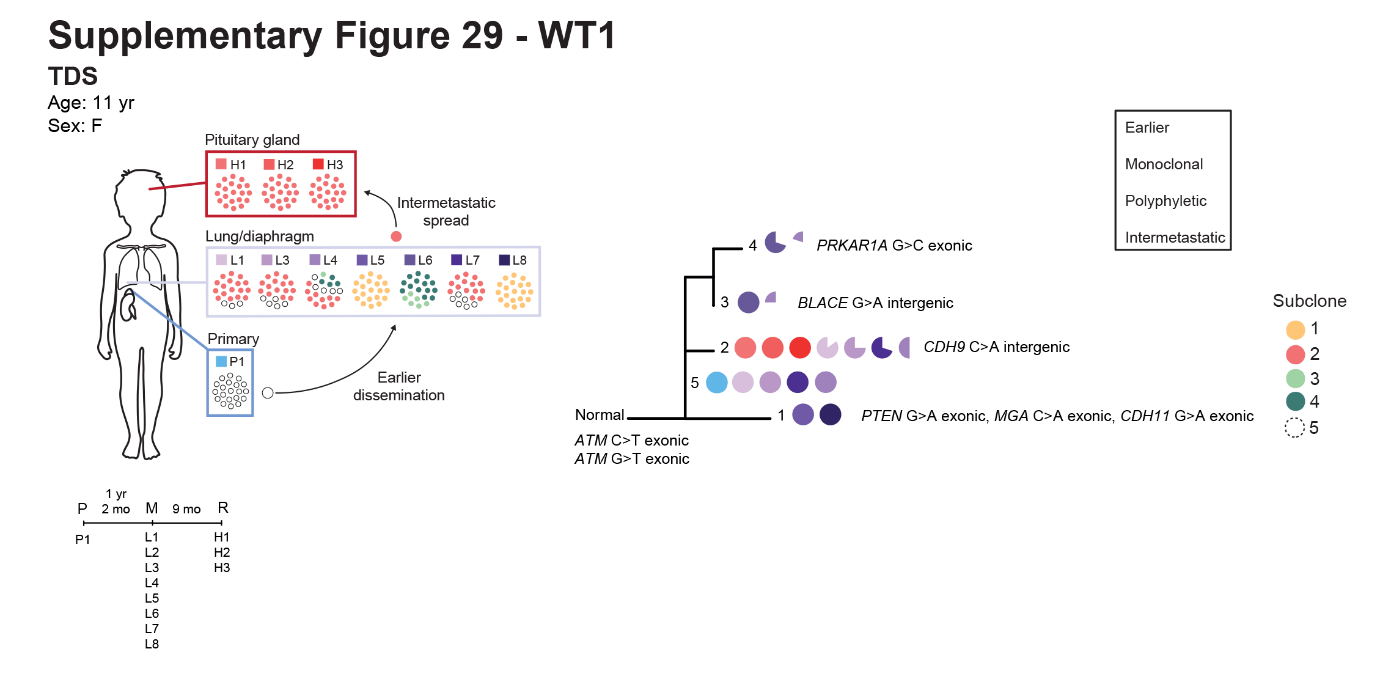
**

**Figure S29. Wilms tumor patient 1: targeted deep sequencing (TDS) data.** The patient presented with a primary tumor in the right kidney (P1). It only contained a single subclone. After 1 year and 2 months the patient presented with a metastasis located in the lung, close to the diaphragm (L1–L8), having five different subclones. One of these were later identified in a brain metastasis to the pituitary gland (H1–H3), detected 9 months later, indicating intermetastatic spread from the lung to the brain.

**
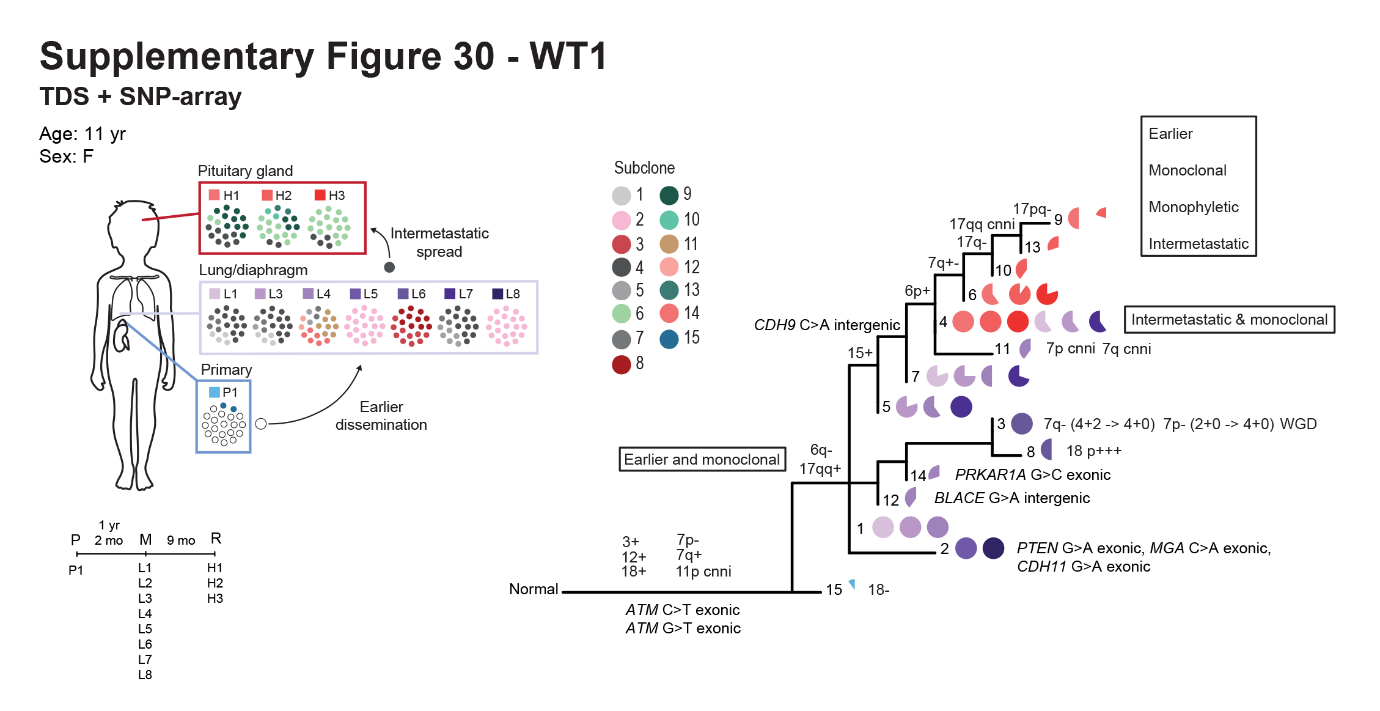
**

**Figure S30. Wilms tumor patient 1: targeted deep sequencing (TDS) and single nucleotide polymorphism (SNP)-array data.** The patient presented with a primary tumor in the right kidney (P1). It only contained two subclones. After 1 year and 2 months the patient presented with a metastasis located in the lung, close to the diaphragm (L1–L8), having five different subclones. One of these were later identified in a brain metastasis to the pituitary gland (H1–H3), detected 9 months later, indicating intermetastatic spread from the lung to the brain.

**
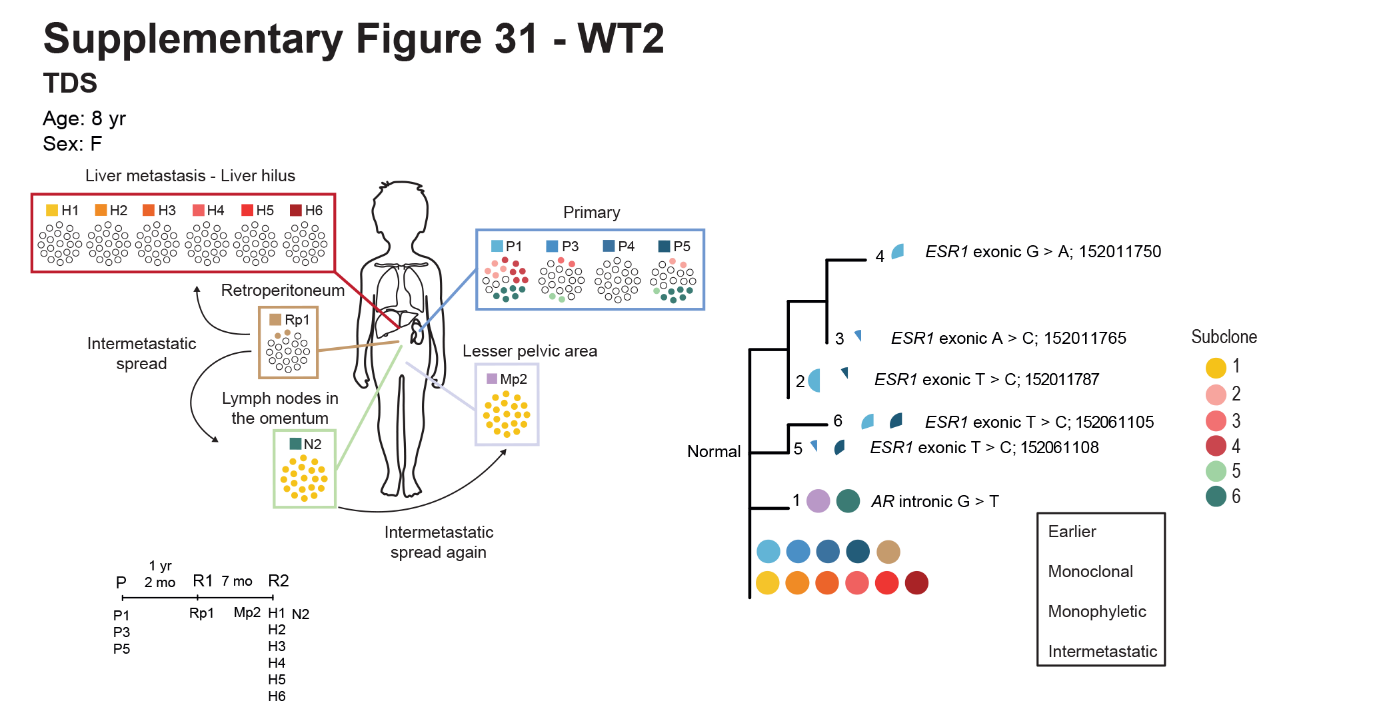
**

**Figure S31. Wilms tumor patient 2: targeted deep sequencing (TDS) data.** The patient presented with a primary tumor in the left kidney (P1, P3, P5). After 1 year and 2 months a metastasis was identified in the retroperitoneum. (Rp1). There was no SNVs detected in this metastasis nor the liver metastasis (H1-6). One mutation was shared between a lymph node in the omentum and the metastasis in the minor pelvis, suggesting intermetastatic spread.

**
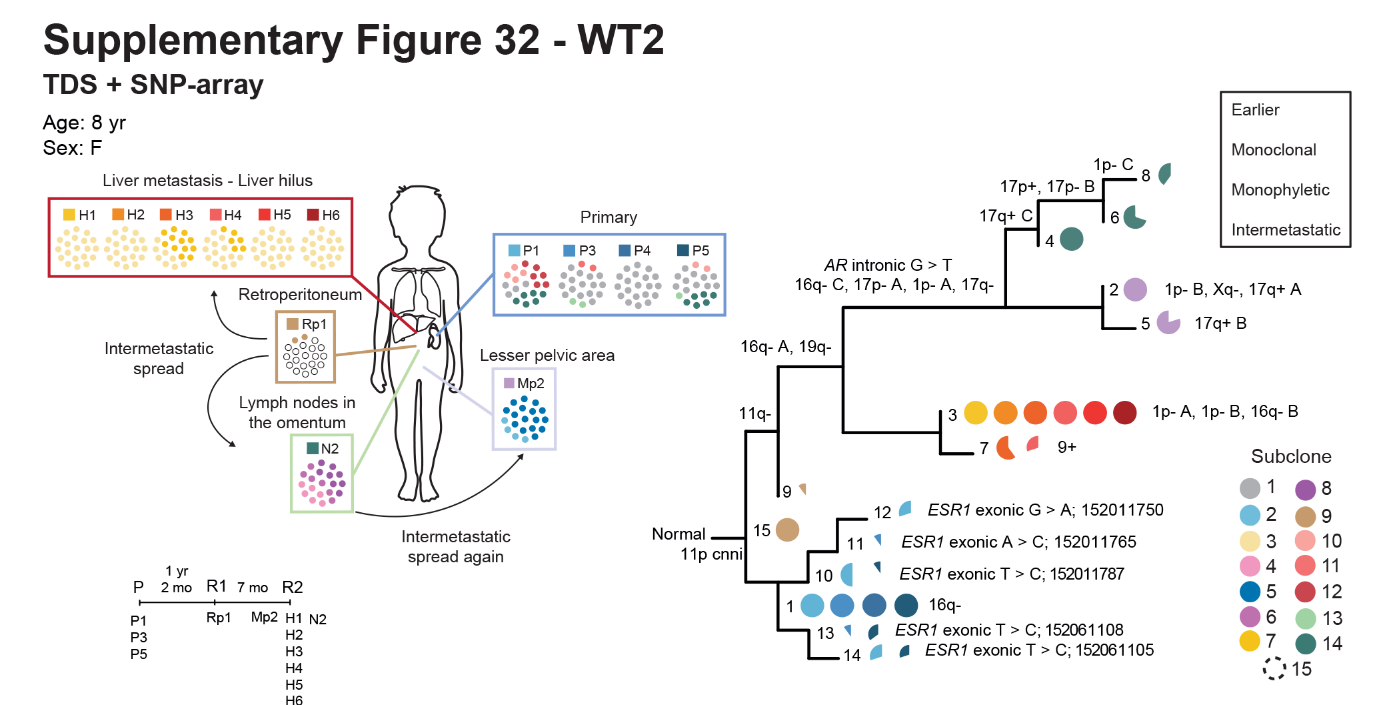
**

**Figure S32. Wilms tumor patient 2: targeted deep sequencing (TDS) data and single nucleotide polymorphism (SNP)-array data.** The patient presented with a primary tumor in the left kidney (P1, P3, P5). After 1 year and 2 months a metastasis was identified in the retroperitoneum (Rp1), which had spread early and monoclonally. Strikingly, both the liver metastasis (H1–H6), metastasis in the minor pelvis (Mp2) and lymph nodes in the omentum (N2) are descendent to one minor subclone in Rp1 (subclone 11). There is also intermetastatic spread between the lymph nodes in the omentum and the lesser pelvic area, suggesting that intermetastatic spread can occur in a stepwise manner.

**
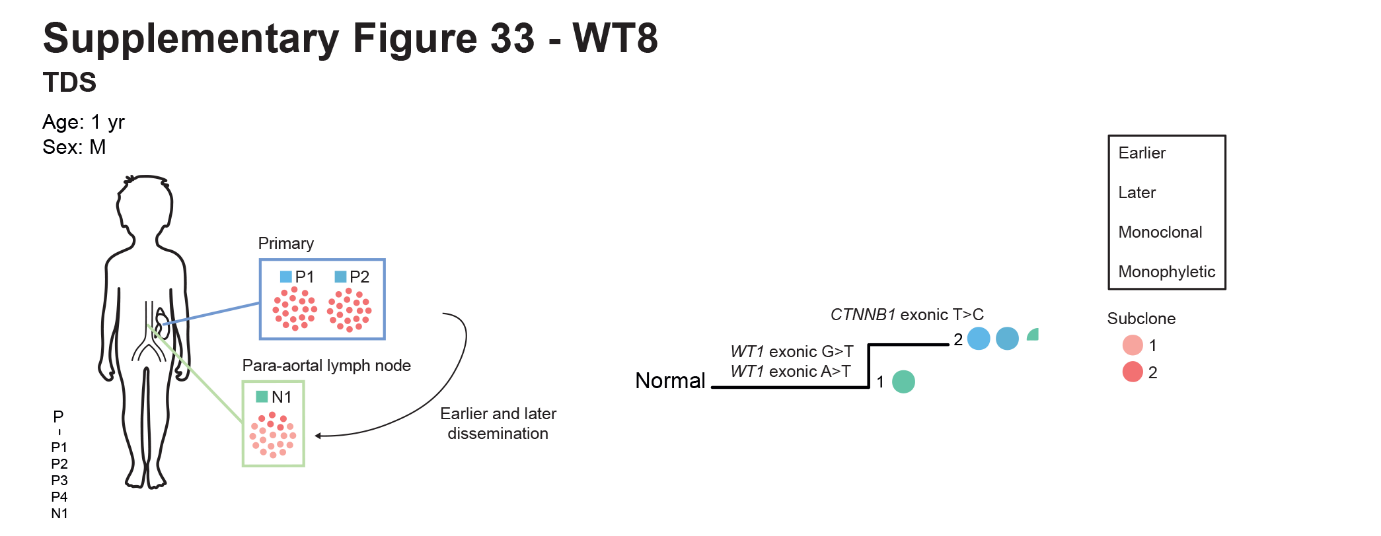
**

**Figure S33. Wilms tumor patient 8: targeted deep sequencing (TDS) data.** The patient presented with a primary tumor in the left kidney (P1, P2). The patient also had a lymph node metastasis para-aortally (N1) both subclone 1 and subclone 2 are found in the metastasis, indicating both evolutionarily early and late metastatic spread.

**
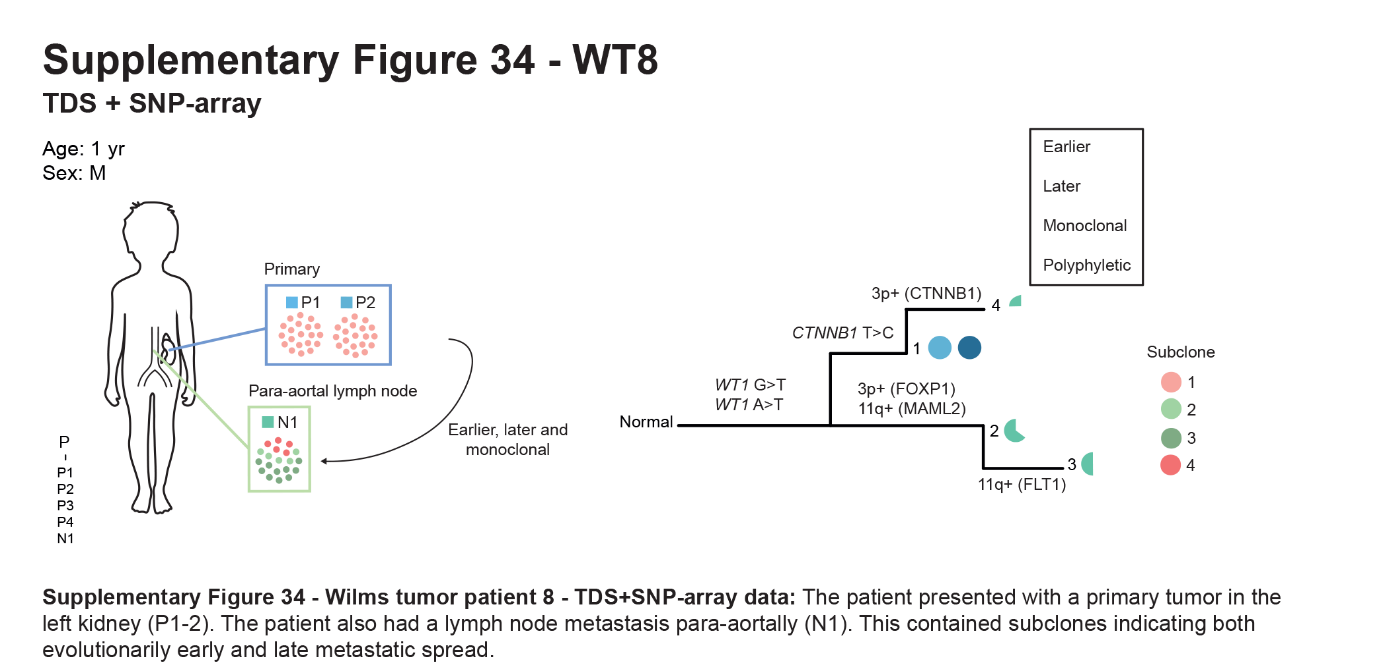
**

**Figure S34. Wilms tumor patient 8: targeted deep sequencing (TDS) and single nucleotide polymorphism (SNP)-array data.** The patient presented with a primary tumor in the left kidney (P1, P2). The patient also had a lymph node metastasis para-aortally (N1). This contained subclones indicating both evolutionarily early and late metastatic spread.

**
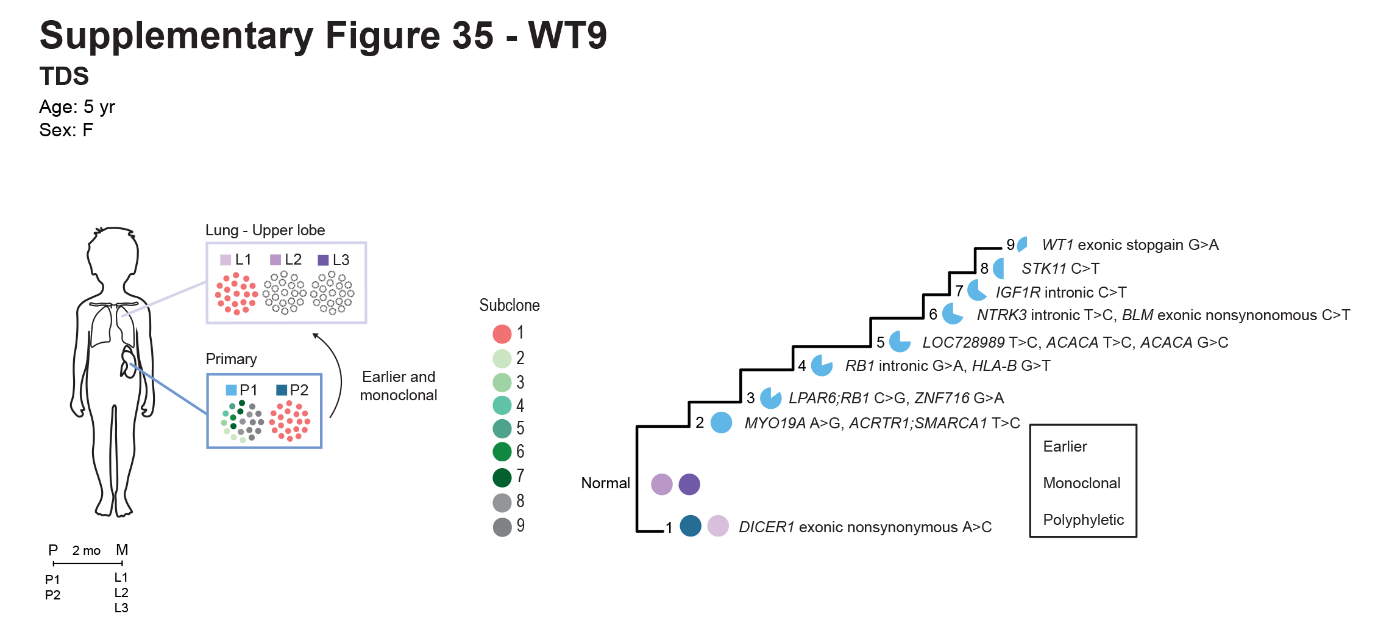
**

**Figure S35. Wilms tumor patient 9: targeted deep sequencing (TDS) data.** The patient presented with a primary tumor in the left kidney (P1, P2). It had a subclonal landscape with only two different subclones. After 2 months the patient presented with a lung metastasis. One of the primary tumor samples share subclone 1 with one of the samples from the lung metastasis.

**
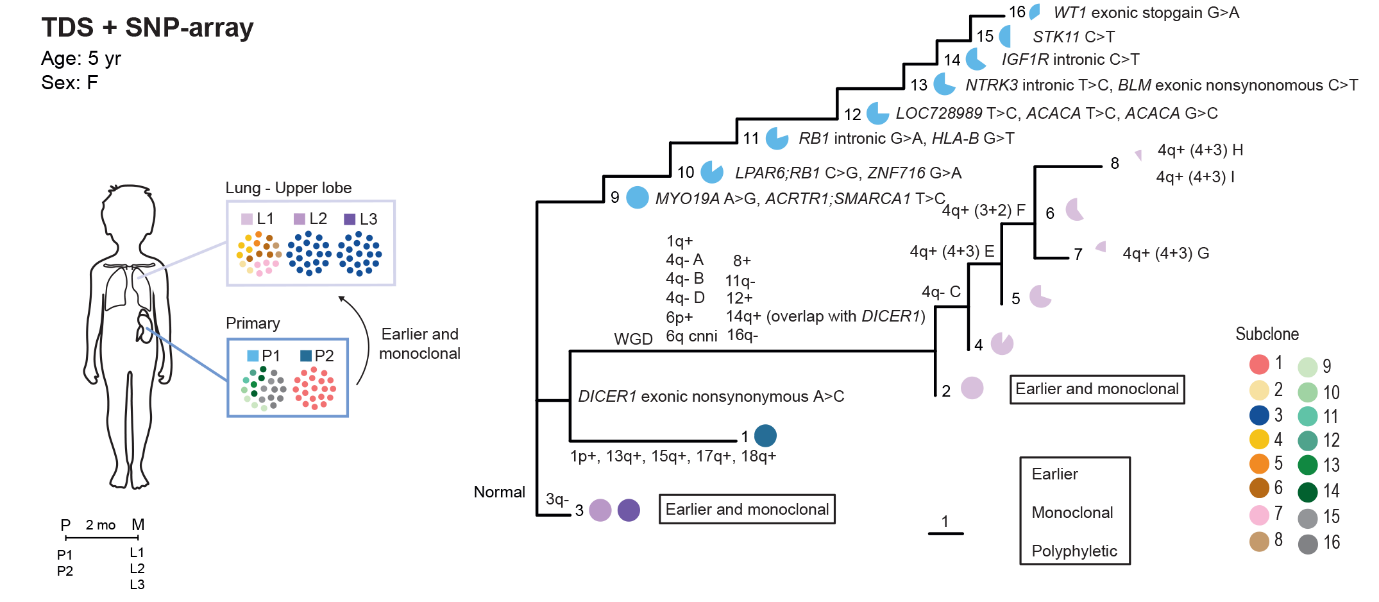
**

**Figure S36. Wilms tumor patient 9: targeted deep sequencing (TDS) and single nucleotide polymorphism (SNP)-array data.** The patient presented with a primary tumor in the left kidney (P1, P2). It had a subclonal landscape with only two different subclones. After 2 months the patient presented with a lung metastasis. One of the primary tumor samples share subclone 1 with one of the samples from the lung metastasis.

**
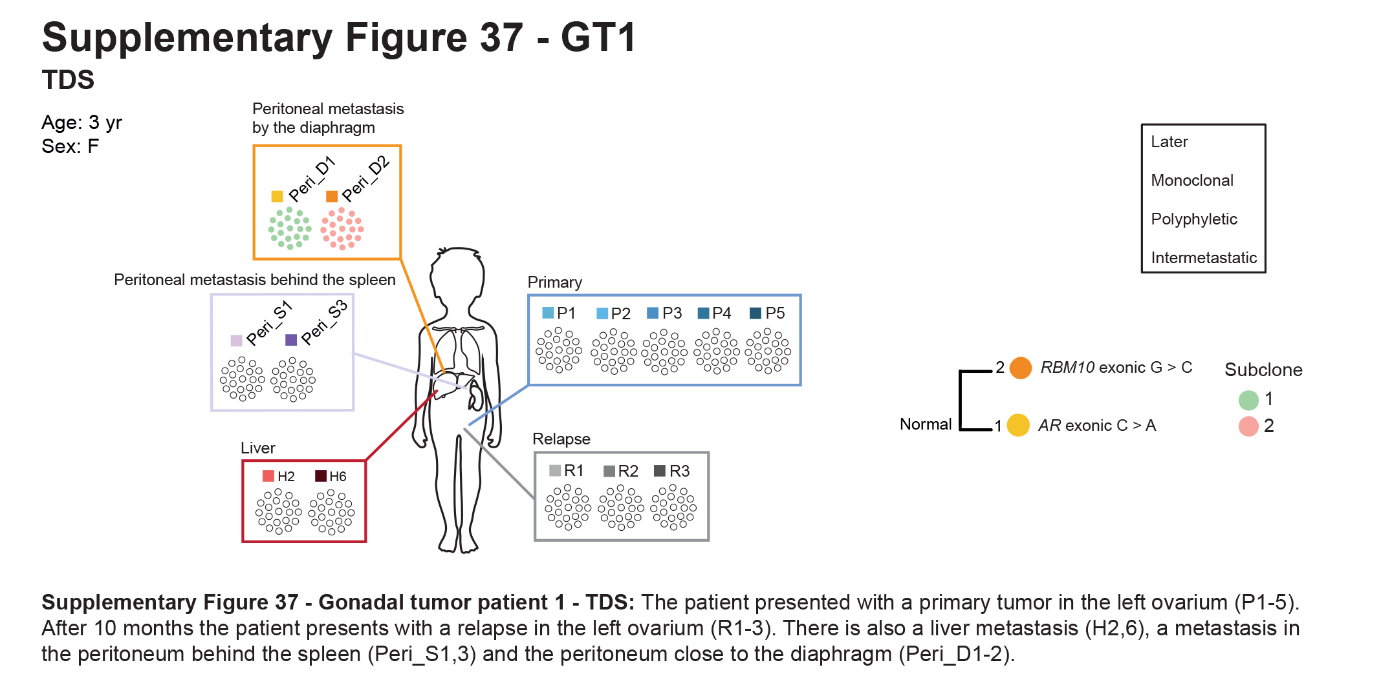
**

**Figure S37. Gonadal tumor patient 1: targeted deep sequencing (TDS) data.** The patient presented with a primary tumor in the left ovarium (P1–P5). After 10 months the patient presents with a relapse in the left ovarium (R1–R3). There is also a liver metastasis (H2, H6), a metastasis in the peritoneum behind the spleen (Peri_S1, S3) and the peritoneum close to the diaphragm (Peri_D1, D2).

**
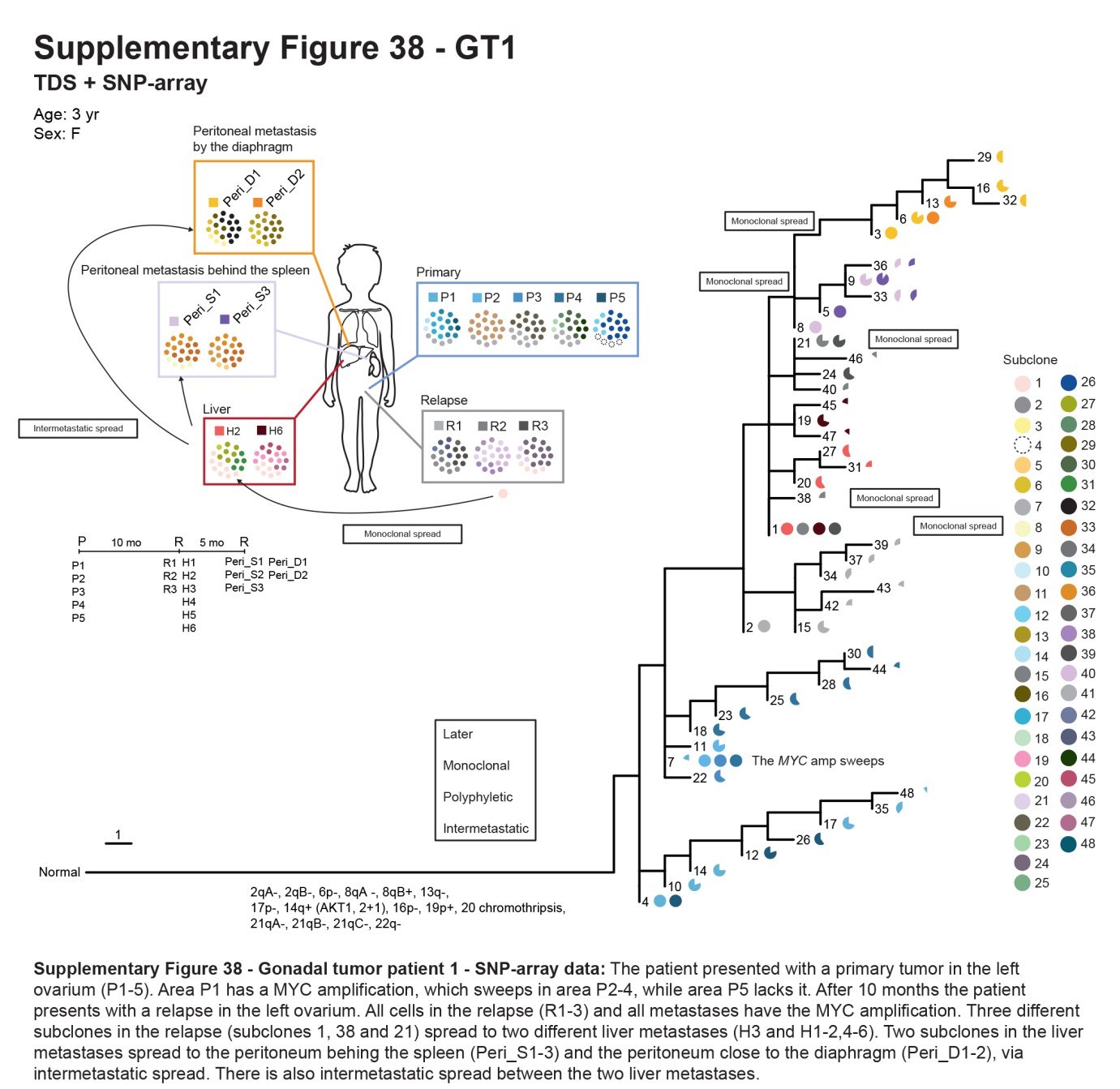
**

**Figure S38. Gonadal tumor patient 1: Targeted deep sequencing (TDS) and single nucleotide polymorphism (SNP)-array data.** The patient presented with a primary tumor in the left ovarium (P1–P5). Area P1 has a MYC amplification, which sweeps in area P2–P4, while area P5 lacks it. After 10 months the patient presents with a relapse in the left ovarium. All cells in the relapse (R1–R3) and all metastases have the MYC amplification. Three different subclones in the relapse (subclones 1, 38 and 21) spread to two different liver metastases (H3, and H1, H2, H4–H6). Two subclones in the liver metastases spread to the peritoneum behind the spleen (Peri_S1–S3) and the peritoneum close to the diaphragm (Peri_D1, D2), via intermetastatic spread. There is also intermetastatic spread between the two liver metastases.

**
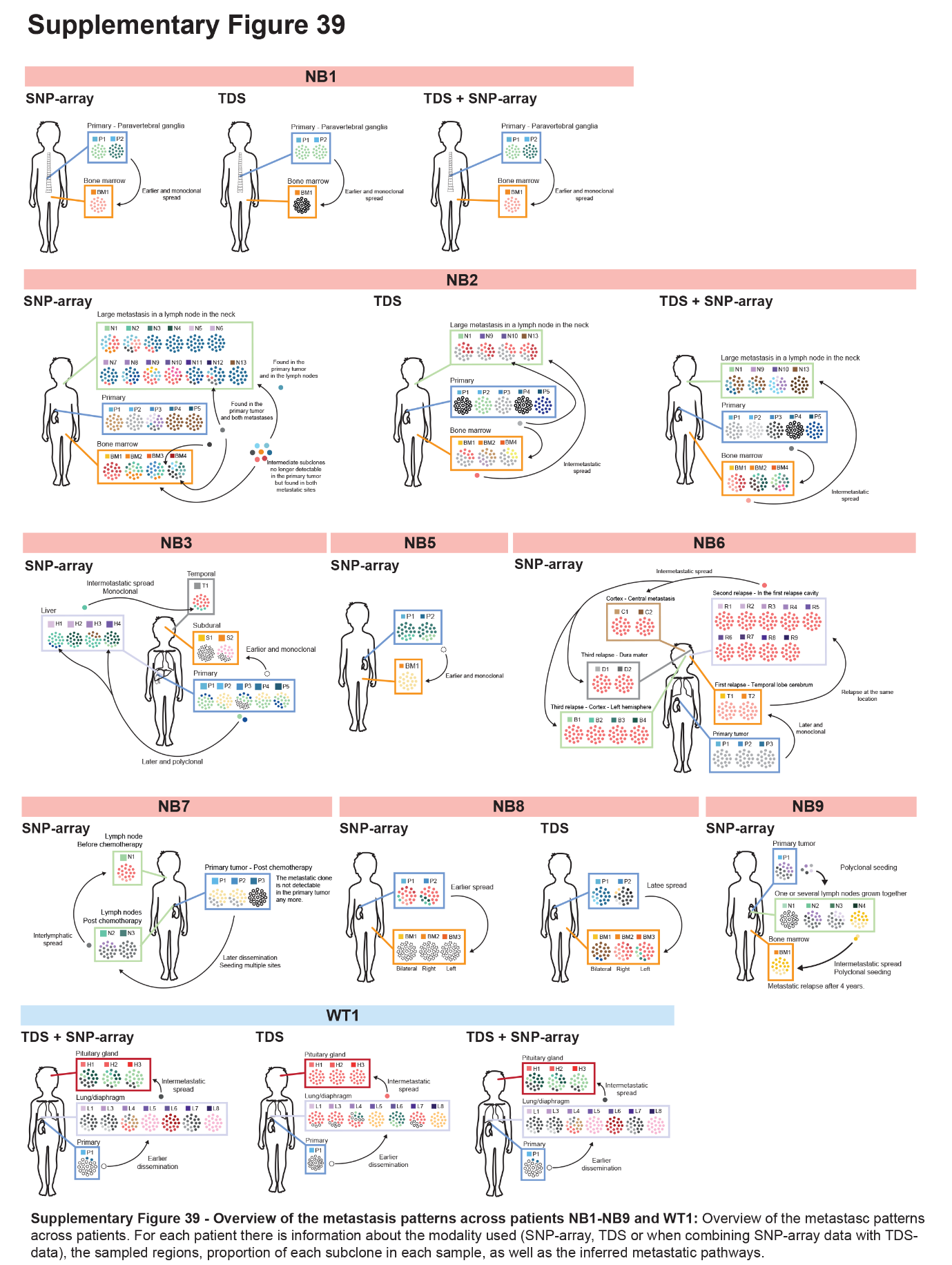
**

**Figure S39. Overview of the metastasis patterns across patients NB1–NB9 and WT1.** Overview of the metastasis patterns across patients. For each patient there is information about the modality used (single nucleotide polymorphism (SNP)-array, targeted deep sequencing (TDS) or when combining SNP-array data with TDS- data), the sampled regions, proportion of each subclone in each sample, as well as the inferred metastatic pathways.

**
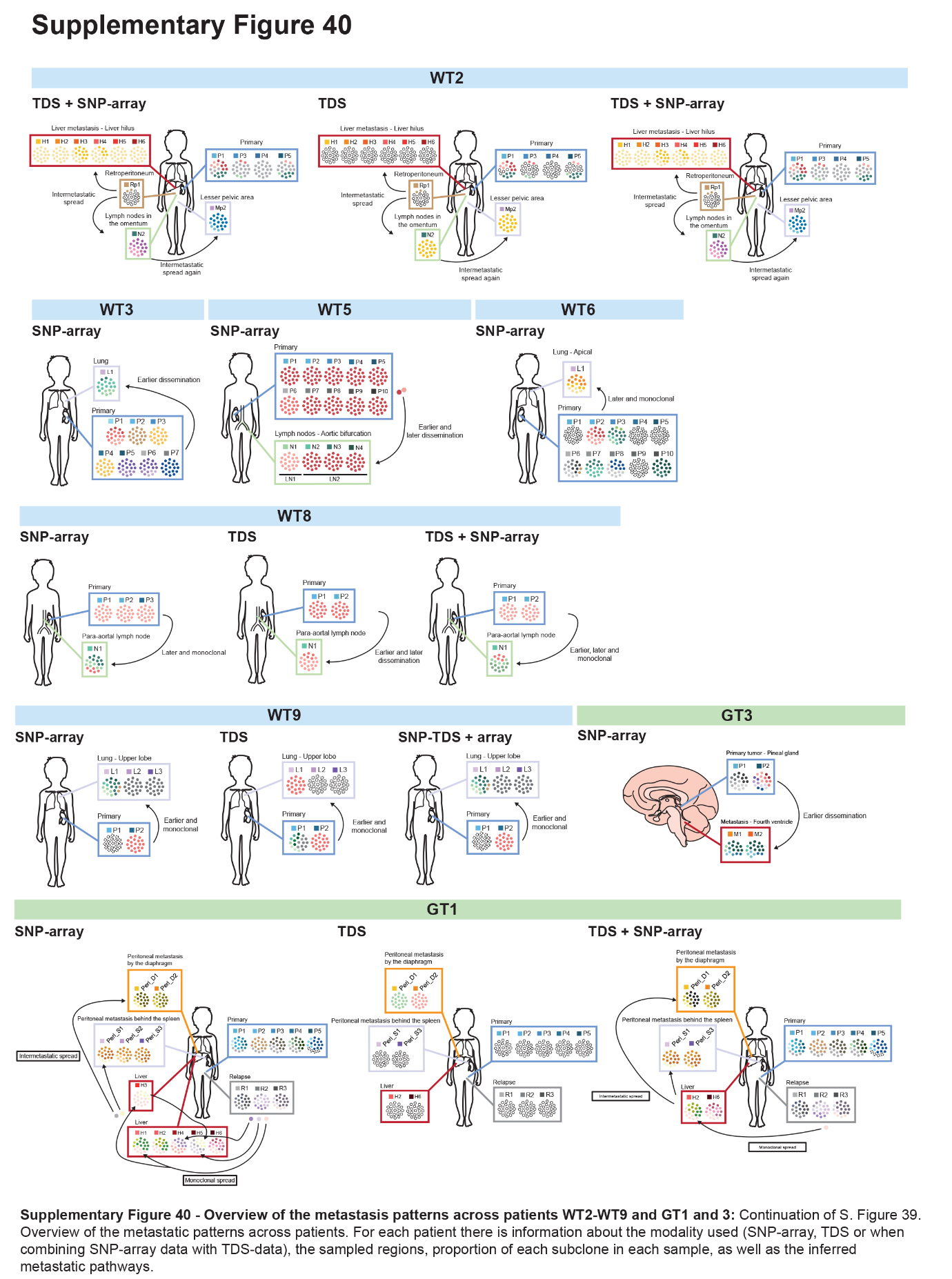
**

**Figure S40. Overview of the metastasis patterns across patients WT2–WT9 and GT1 and GT3.** Continuation of Figure S39. Overview of the metastatic patterns across patients. For each patient there is information about the modality used (SNP-array, targeted deep sequencing (TDS) or when combining SNP-array data with TDS-data), the sampled regions, proportion of each subclone in each sample, as well as the inferred metastatic pathways.
