# Supplementary material for: Tracing metastatic spread in pediatric solid tumors using copy number and targeted deep sequencing
Source: J Pathol. 2025 Sep 23;267(3):347–65. doi: 10.1002/path.6472 (PMC12531126; doi:10.1002/path.6472)
Supplement: Supplementary file 4 — Figure S41. Copy number heatmaps [file PATH-267-347-s010.docx]

**Tracing metastatic spread in pediatric solid tumors using copy number and targeted deep sequencing**

N Andersson *et al. J Pathol* <https://doi.org/10.1002/path.6472>

**Supplementary Figure S41**

**
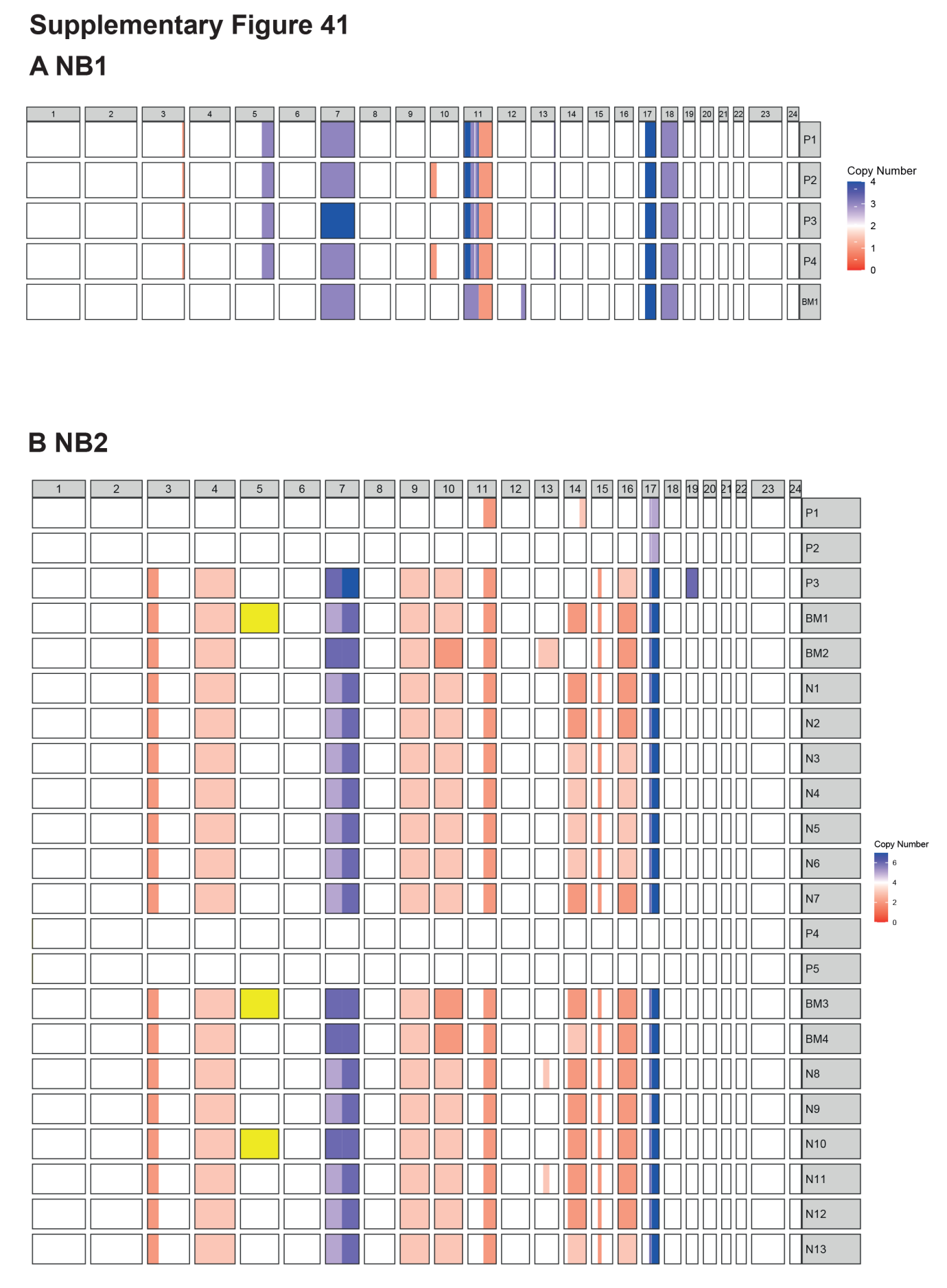
**

**
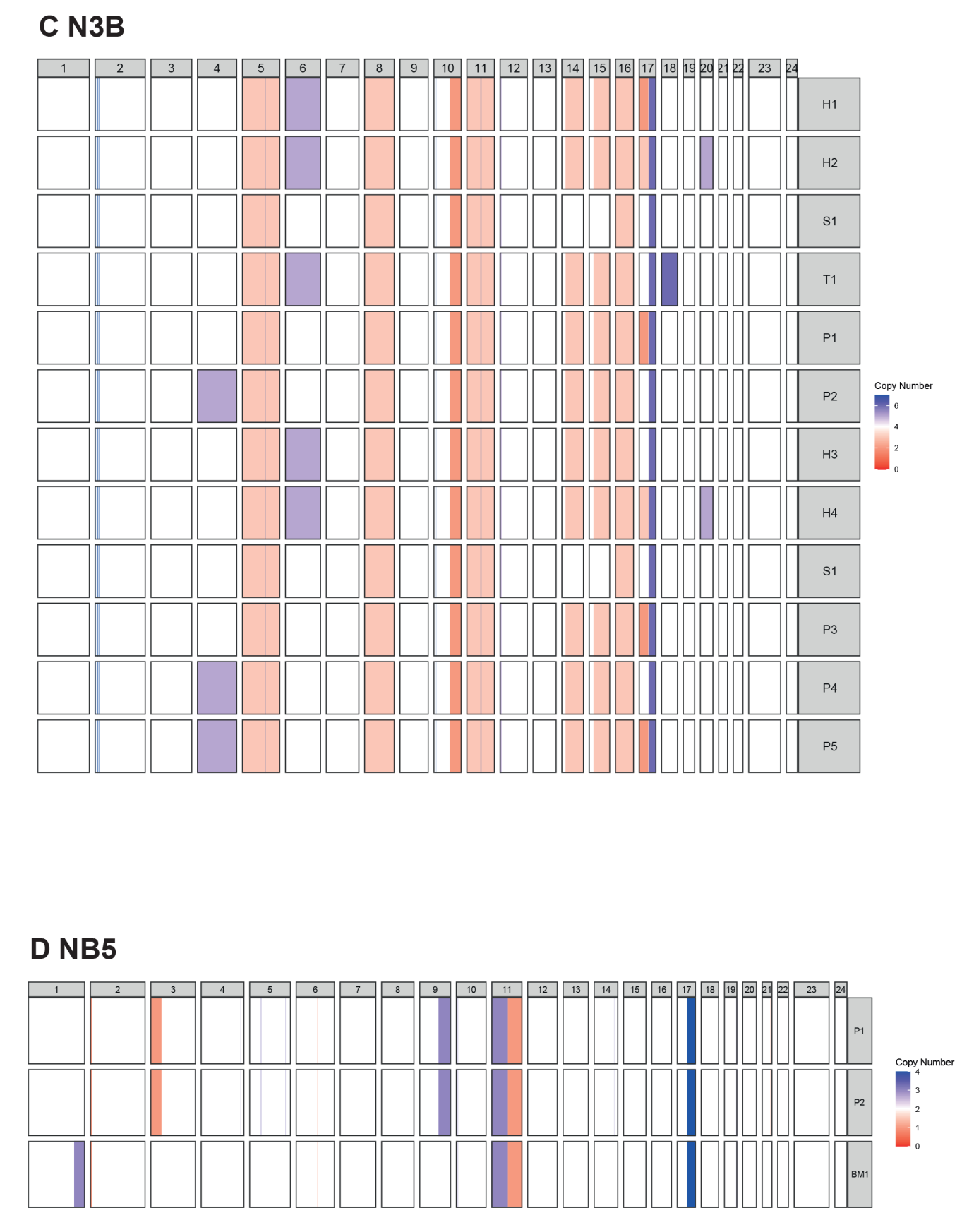
**

**
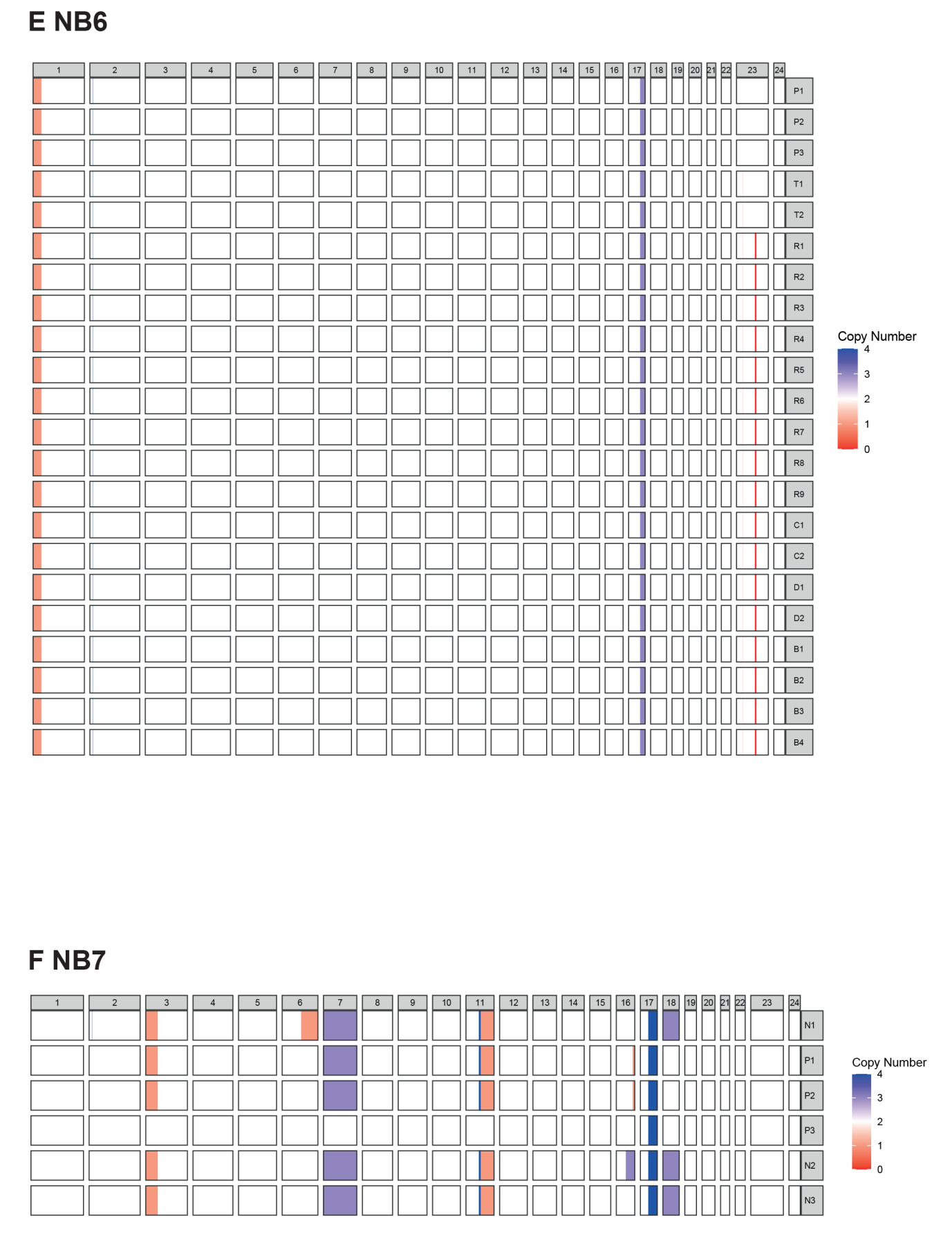
**

**
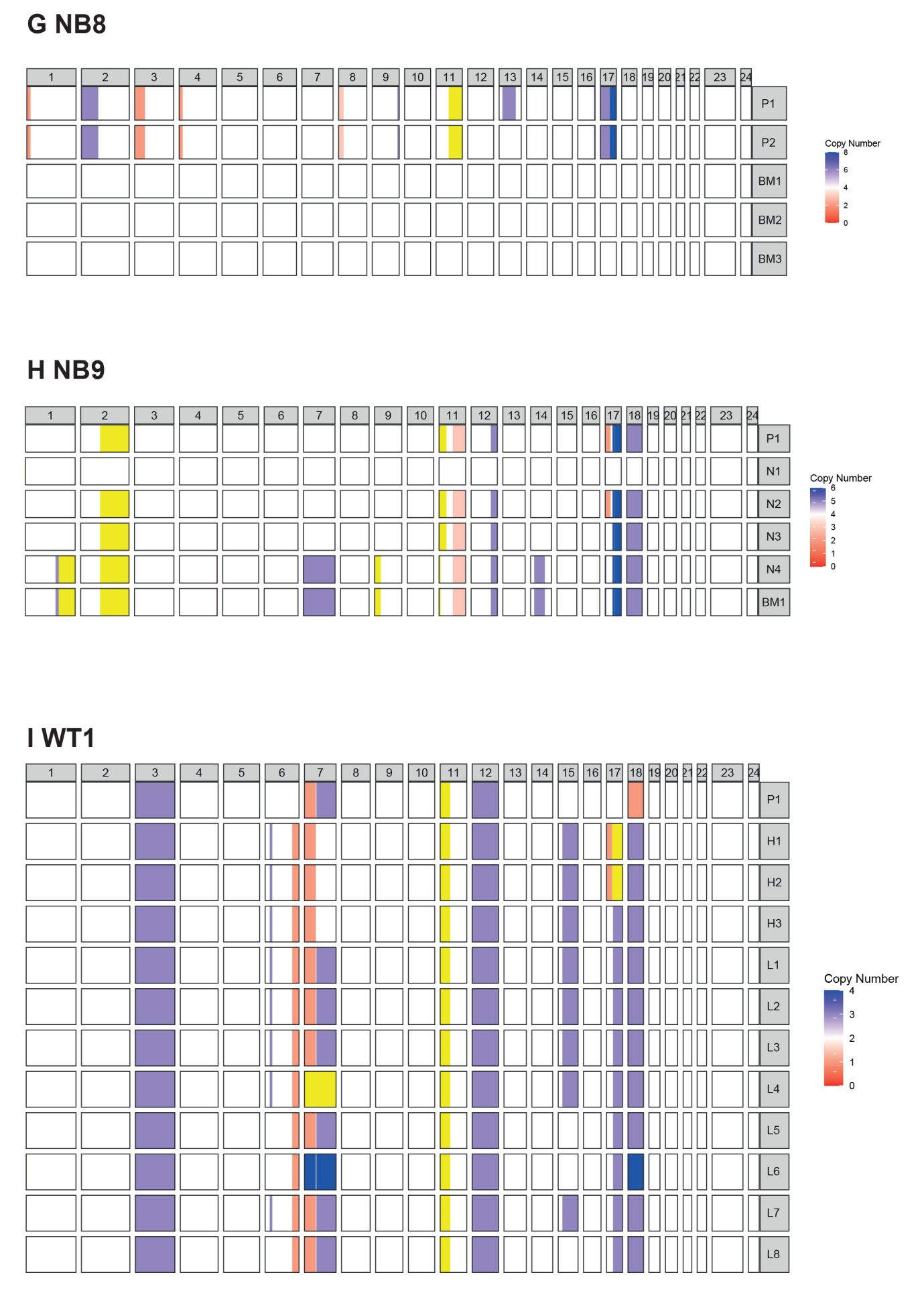
**

**
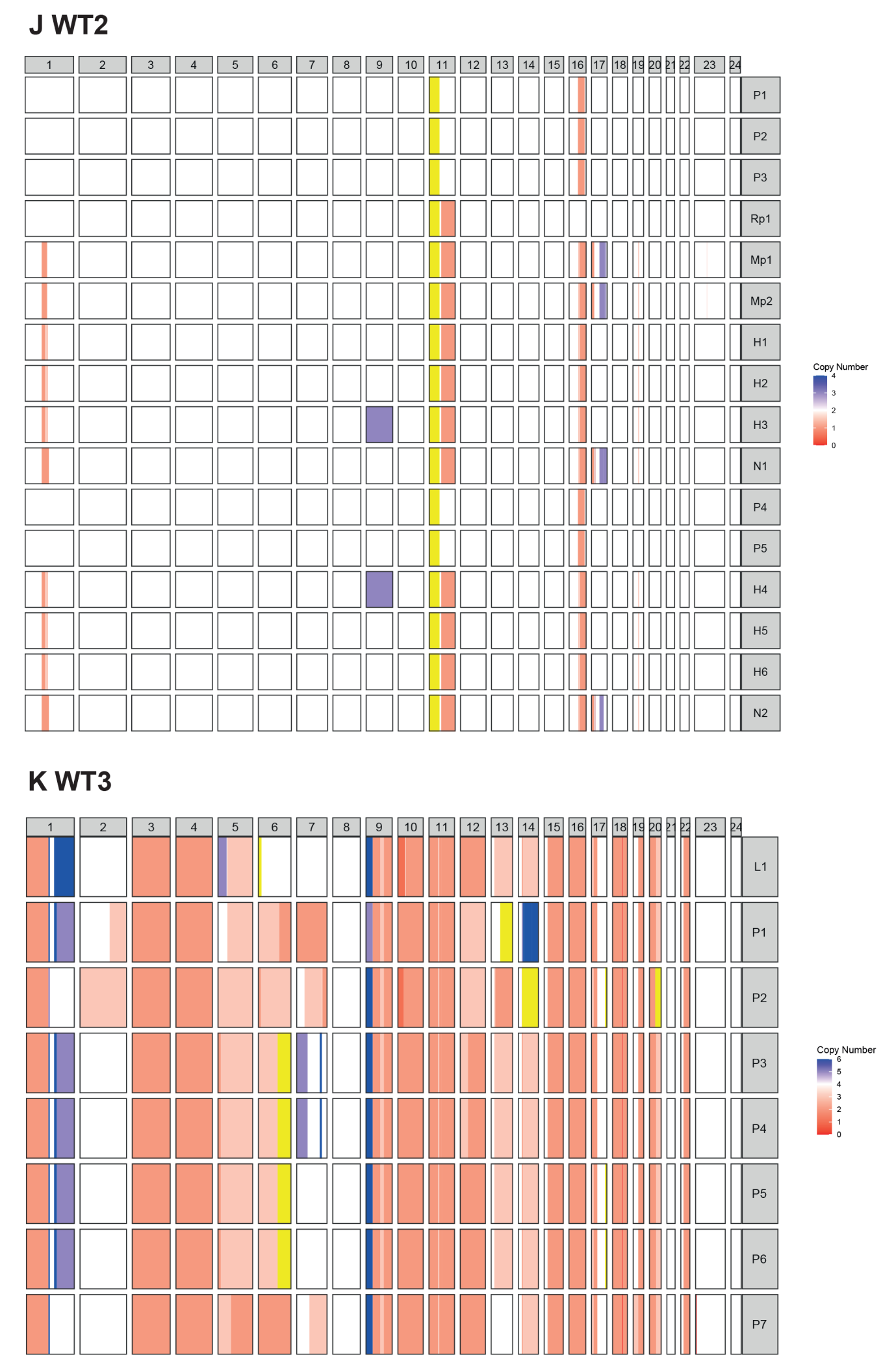
**

**
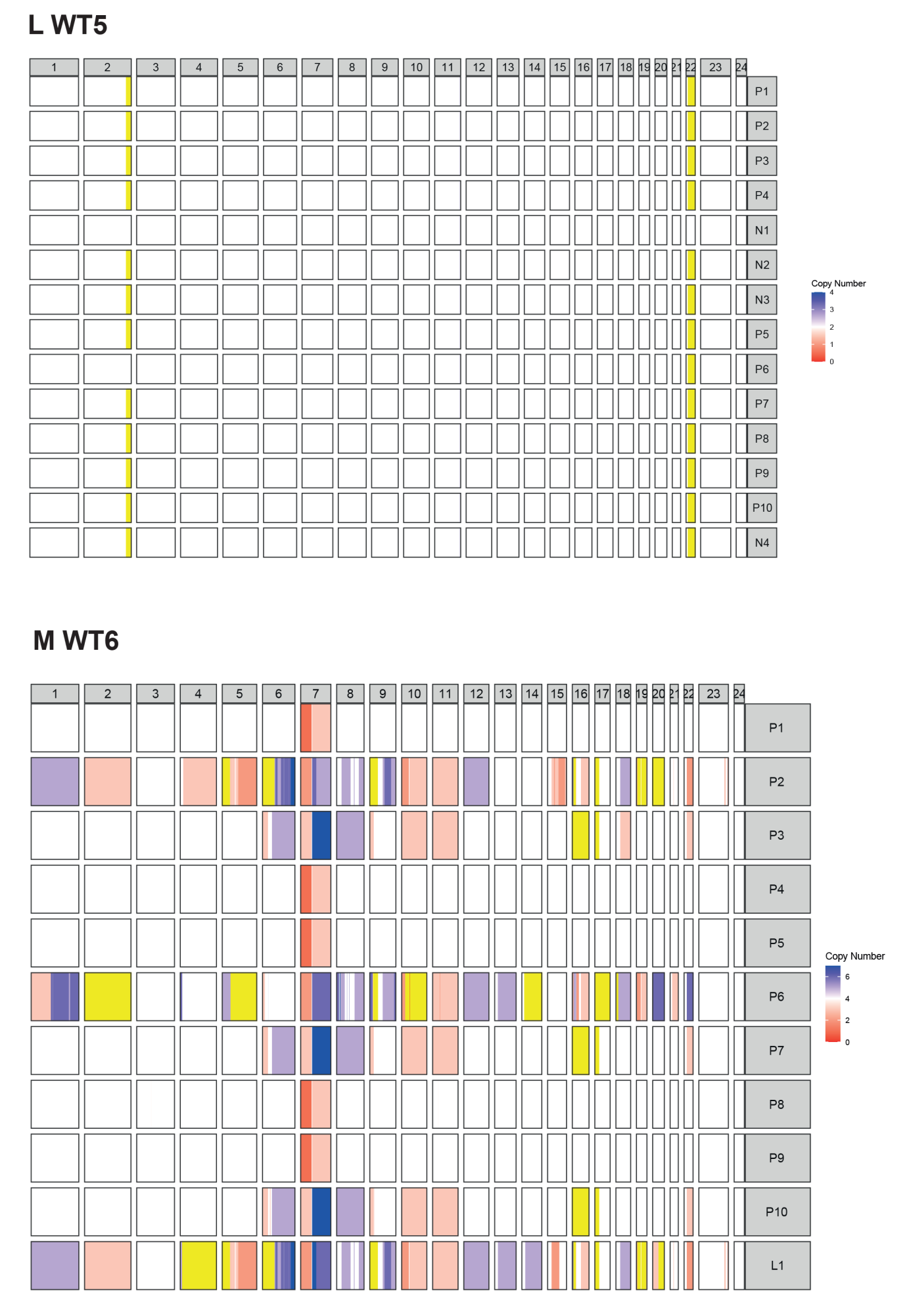
**

**
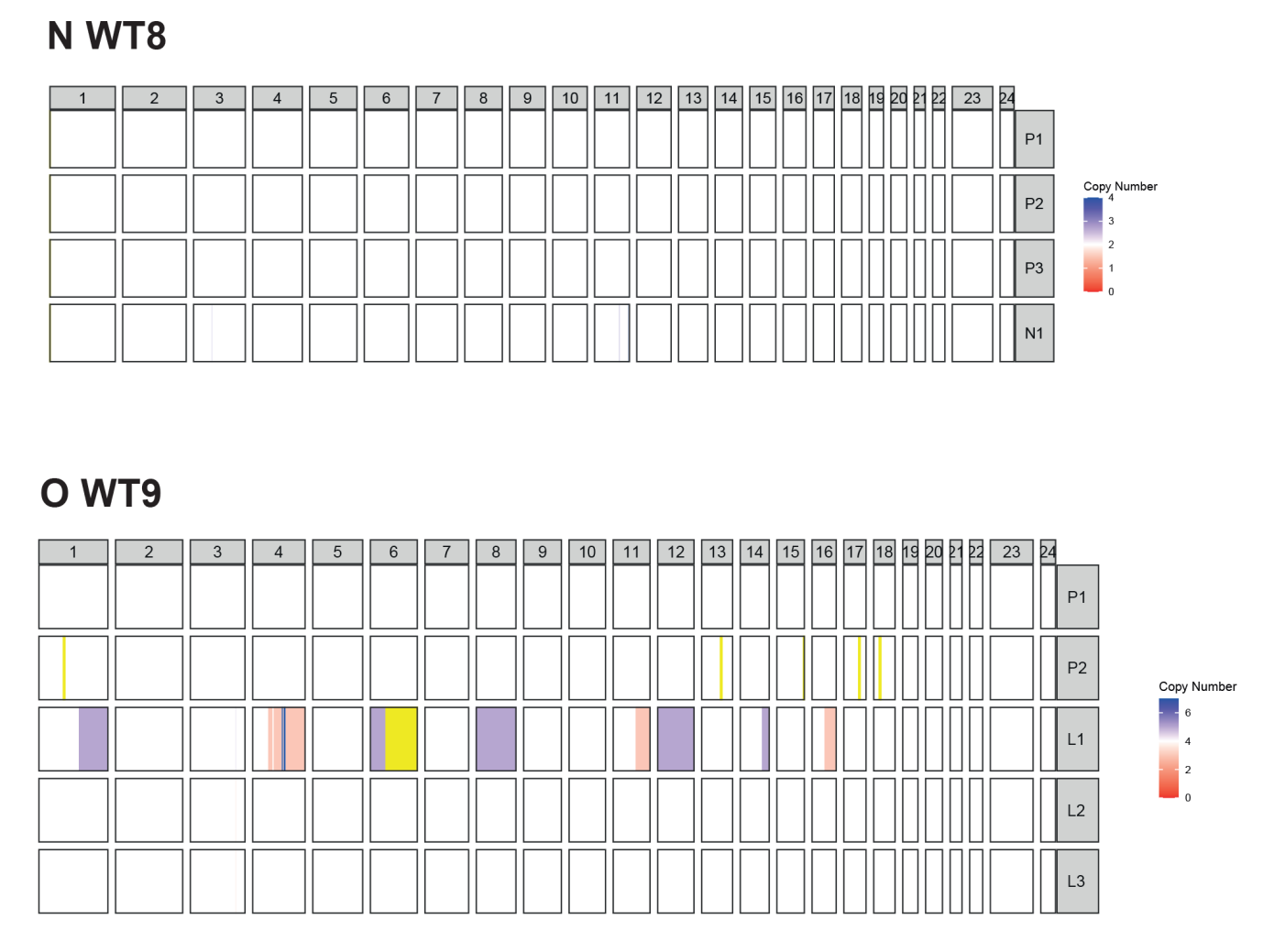
**

**
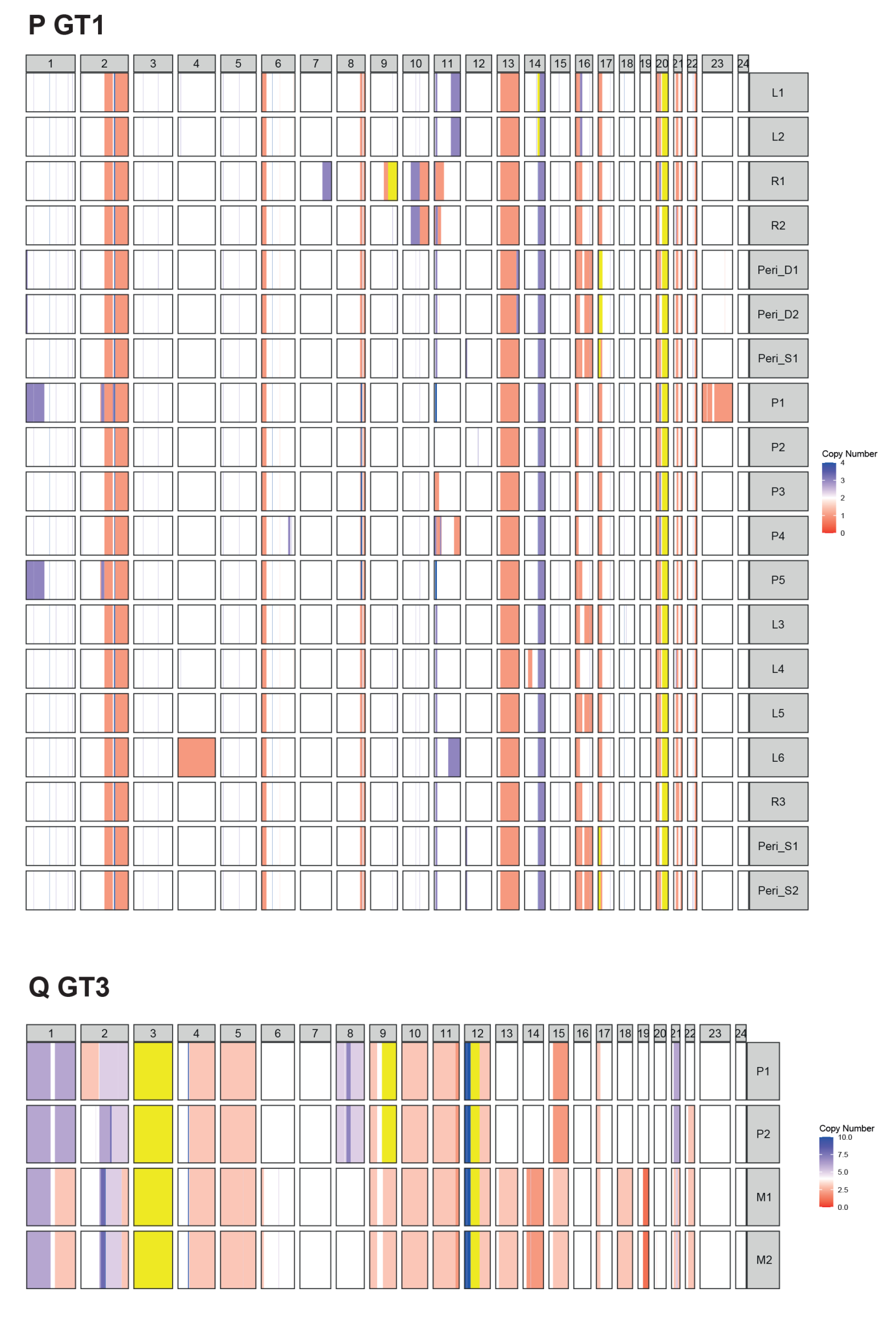
**

**Figure S41. Copy number heatmaps**. (A–Q) Heatmaps illustrating the copy number alterations identified in each sample from each patient. The patient name is found above each heatmap. Each column represents a chromosome. In the column headers, the chromosome is indicated. Each row represents a sample, whose name is found in the rightmost gray box. A detailed description of the precise anatomic location of each sample can be seen in the corresponding patient’s metastatic trajectory plot (supplementary material, Figures S5–S38). To the right of each figure a legend is found that indicates which color in the heatmap represents which copy number. Red indicates a copy number loss and blue a gain of that particular chromosomal segment. Yellow suggests a copy number neutral imbalance such as 2+0 for a diploid tumor, or 4+0 or 3+1 for a tetraploid tumor.
