# Supplementary material for: Tracing metastatic spread in pediatric solid tumors using copy number and targeted deep sequencing
Source: J Pathol. 2025 Sep 23;267(3):347–65. doi: 10.1002/path.6472 (PMC12531126; doi:10.1002/path.6472)
Supplement: Supplementary file 5 — Figure S42. Heatmap of all copy number alterations across all samples and patients Figure S43. Heatmap of the sum of copy number alterations across all samples and patients Figure S44. Heatmap of all metastasis unique copy number alterations across all metastasis samples Figure S45. Heatmap of all metastasis unique single nucleotide variants across all metastasis samples. Figure S46. Duplication signature in gonadal tumor patient GT1 [file PATH-267-347-s003.docx]

**Tracing metastatic spread in pediatric solid tumors using copy number and targeted deep sequencing**

N Andersson *et al. J Pathol* <https://doi.org/10.1002/path.6472>

**Supplementary Figures S42–S46**

**
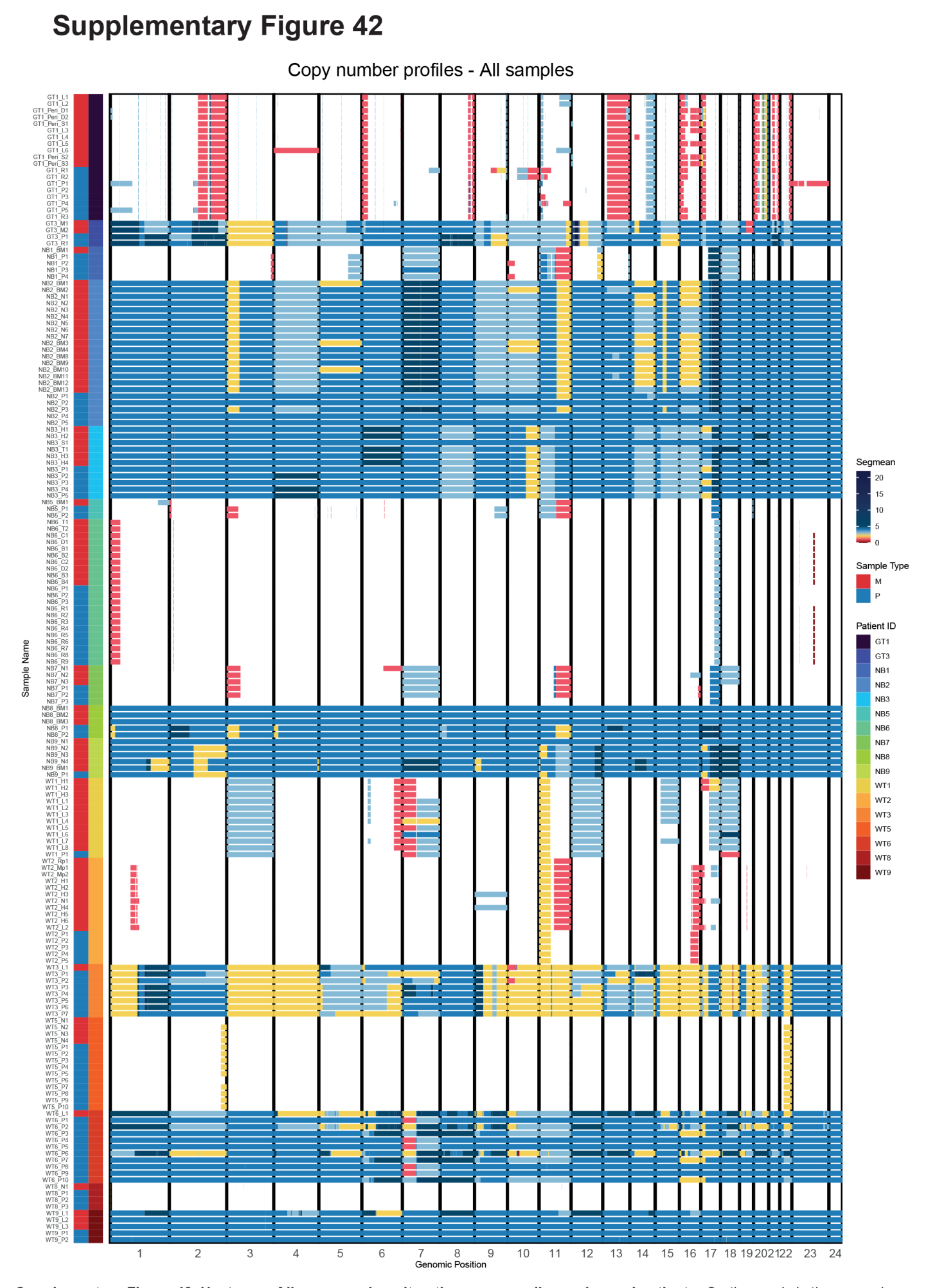
**

**Figure S42. Heatmap of all copy number alterations across all samples and patients.** On the *x*-axis is the genomic position for all 24 chromosomes. On the *y*-axis are all samples across patients along with annotations of which patient each sample belongs to and whether it is a primary of metastasis sample. The elements of the heatmap indicates the copy number status for each genomic position, with blue colors indicating gains, red losses and yellow a copy number neutral imbalance.

**
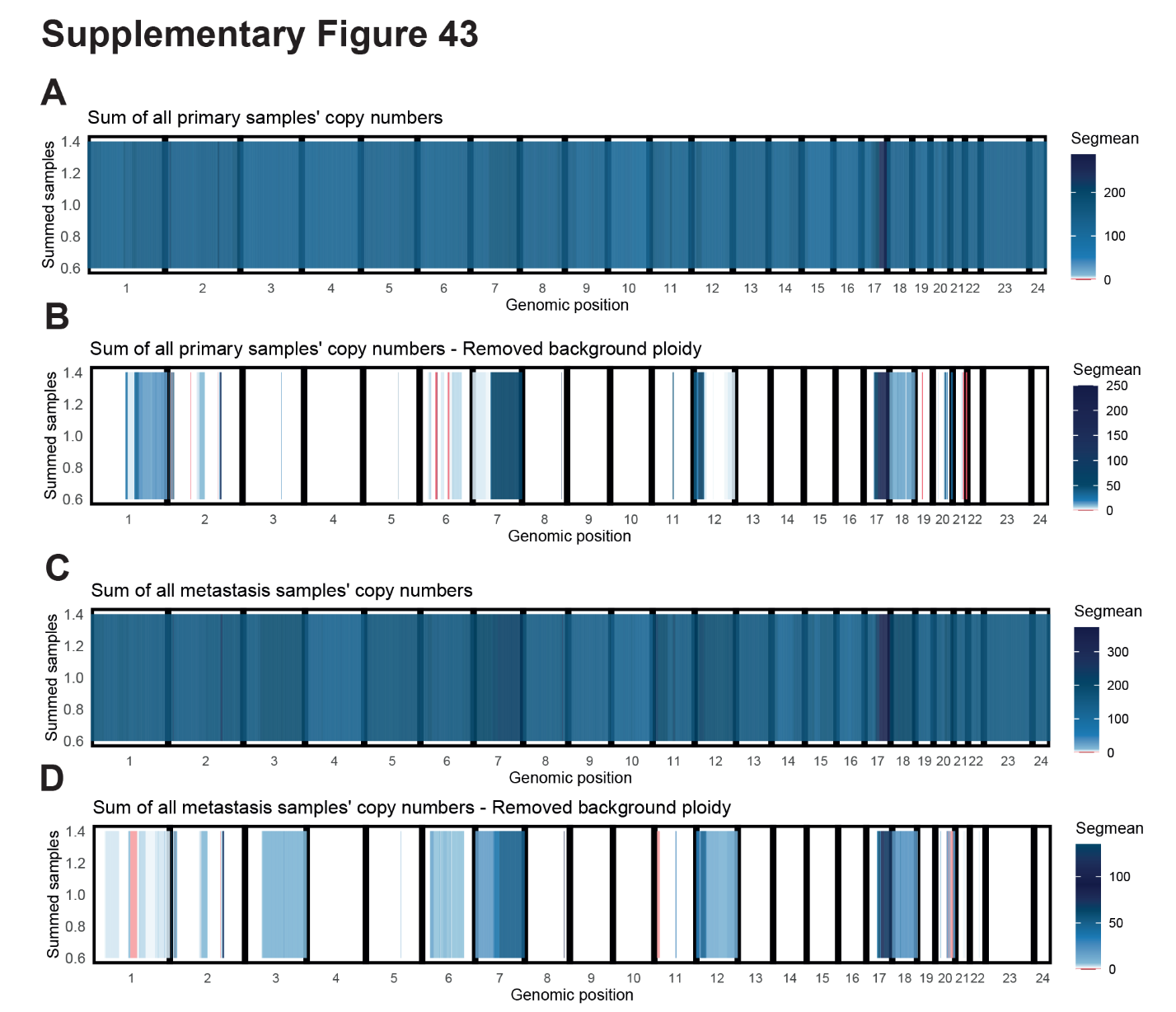
Figure S43. Heatmap of the sum of copy number alterations across all samples and patients.** On the *x*-axis is the genomic position for all 24 chromosomes. The elements of the heatmap indicates the copy number status for each genomic position, with blue colors indicating gains, red losses and yellow a copy number neutral imbalance. (A) Sum of all primary tumor samples’ copy numbers across the genome. (B) The same heatmap as in panel (A) but the background ploidy of each sample has been removed. (C) Sum of all metastasis samples’ copy numbers across the genome. (D) The same heatmap as in panel (C) but the background ploidy of each sample has been removed.

**
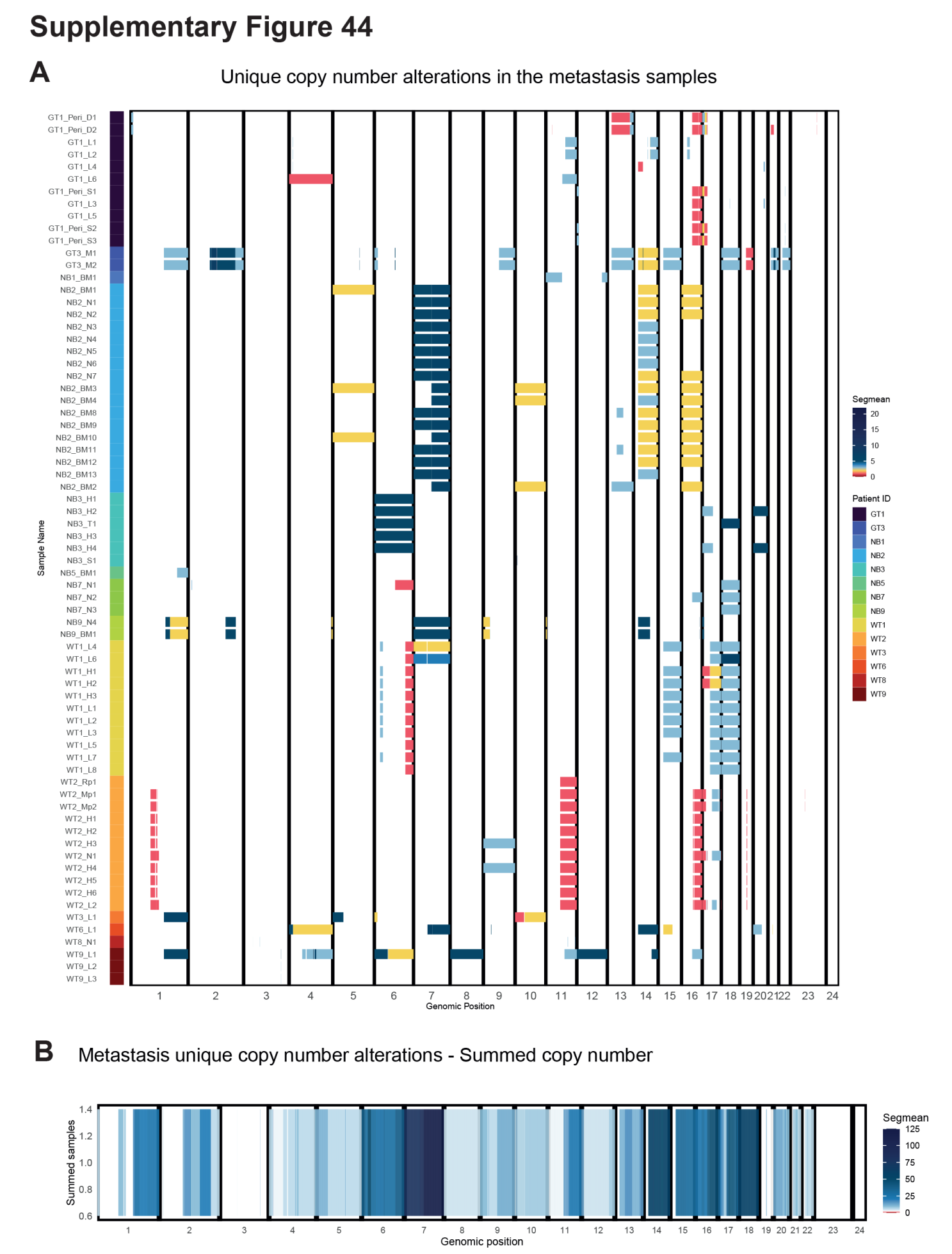
**

**Figure S44. Heatmap of all metastasis unique copy number alterations across all metastasis samples and patients. (**A) On the *x*-axis is the genomic position for all 24 chromosomes. On the *y*-axis are all samples across patients along with annotations of which patient each sample belongs to and whether it is a primary of metastasis sample. The elements of the heatmap indicates the copy number status for each genomic position, with blue colors indicating gains, red losses and yellow a copy number neutral imbalance. (B) The sum of tall metastasis specific copy number alterations for each genomic position across all metastasis samples.

**
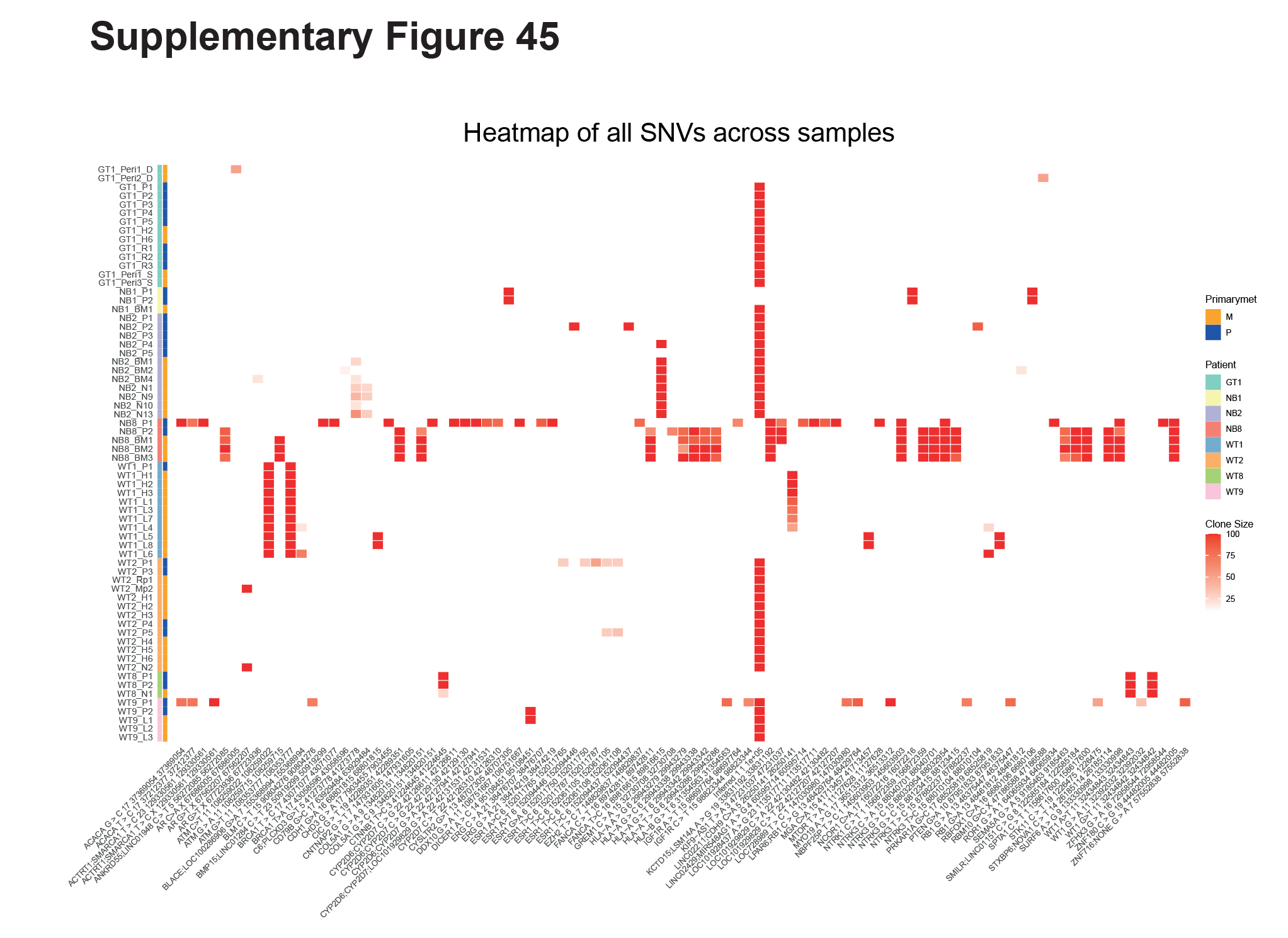
**

**Figure S45. Heatmap of all metastasis unique single nucleotide variants (SNVs) across all metastasis samples and patients.** On the *x*-axis are all single nucleotide variants identified across all analyzed samples. On the *y*-axis are all analyzed samples along with an indication of which patient each sample belong and whether it is a primary tumor or metastasis sample. The heatmap elements are the clone sizes.

**
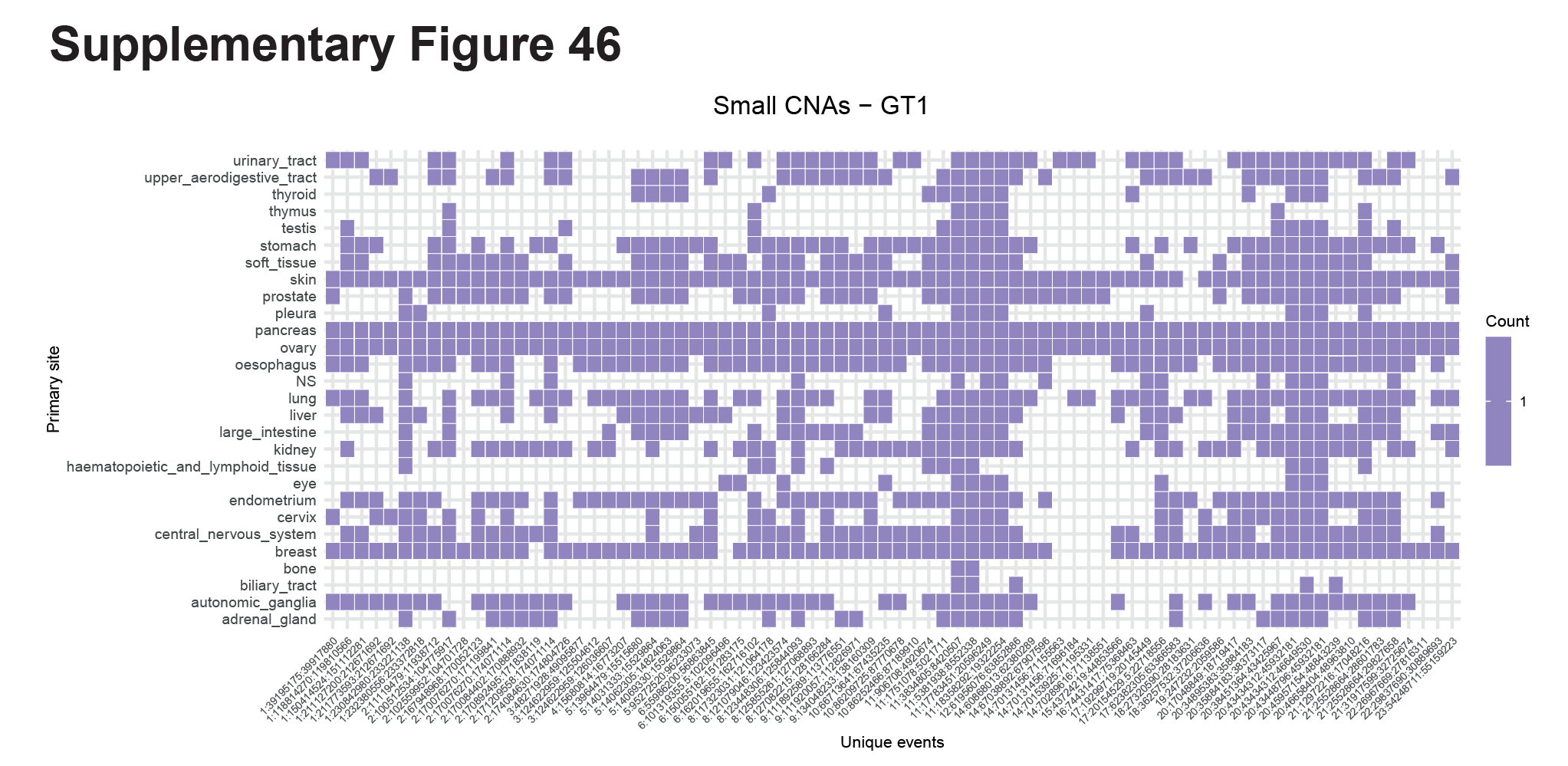
**

**Figure S46. Duplication signature in gonadal tumor patient GT1.** On the *x*-axis each small CNA detected across samples are denoted. On the *y*-axis are primary tumor sites. Each element in the heatmap indicates whether a CNA in that region has been shown to be related to tumors in that particular site, according to the COSMIC data base.
